# Regioselective addition/annulation of ferrocenyl thioamides with 1,3-diynes *via* a sulfur-transfer rearrangement to construct extended π-conjugated ferrocenes with luminescent properties†

**DOI:** 10.1039/d0sc04597g

**Published:** 2020-09-16

**Authors:** Lipeng Yan, Jingbo Lan, Hu Cheng, Yihang Li, Mangang Zhang, Di Wu, Jingsong You

**Affiliations:** Key Laboratory of Green Chemistry and Technology of Ministry of Education, College of Chemistry, Sichuan University 29 Wangjiang Road Chengdu 610064 People's Republic of China jingbolan@scu.edu.cn jsyou@scu.edu.cn

## Abstract

Herein a regioselective addition/annulation strategy of ferrocenyl (Fc) thioamides with alkynes to construct thienylferrocene (ThienylFc) structures, involving a rhodium-catalyzed C–H activation, an unusual C2-selective addition of 1,3-diyne, and an unexpected intramolecular sulfur-transfer rearrangement process is described. In this protocol, thioamide not only serves as a directing group to activate the *ortho*-C–H bond of the ferrocene, but also as a sulfur source to form the thiophene ring. The resulting carboxylic ester group after sulfur transfer can act as a linkage to construct extended π-conjugated ferrocenes (OCTFc) with luminescent properties. ThienylFc displays effective fluorescence quenching due to the photoinduced electron transfer (PET) from the Fc unit to the excited luminophore, which turns out to be a promising type of redox molecular switch. OCTFc exhibit relatively strong emission owing to their intramolecular charge transfer (ICT) characteristics. The ring-fused strategy is herein employed for the first time to construct luminescent materials based on ferrocenes, which provides inspiration for the development of novel organic optoelectronic materials, such as electroluminescent materials based on ferrocenes.

## Abstract 2

Regioselective addition/annulation of ferrocenyl thioamides with 1,3-diynes has been developed to construct extended π-conjugated ferrocenes with luminescent properties.

## Graphical abstract

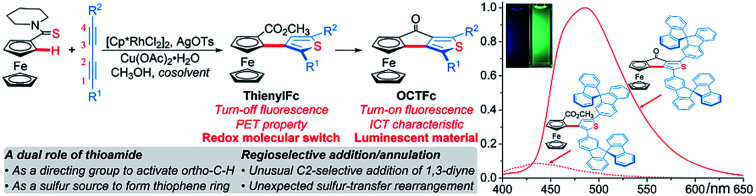

## Introduction

Ferrocene (Fc) is an important building block for versatile ligands, medicines and optoelectronic materials because of its unique sandwich structure, strong π-donating ability, and chemical and thermal stabilities, as well as good reversibility in one-electron oxidation.^1–3^ Since the first synthesis of ferrocene was reported in 1951,^4^ its functionalization and relevant application have drawn increasing attention.^1–3^ In principle, ferrocene can serve as an electron donor (D) to construct luminescent materials with a D–A (A, electron acceptor) structure. However, D–A molecules containing an Fc unit usually show quenched fluorescence due to a photoinduced electron transfer (PET) process from the ferrocene to the excited electron acceptor. Ferrocene can be oxidized to an electron-deficient ferricenium cation (Fc^+^), which terminates the PET process, thus leading to an enhanced emission. Based on this mechanism, various redox-fluorescence switches derived from ferrocene have been developed recently.^5–7^

**Scheme 1:**
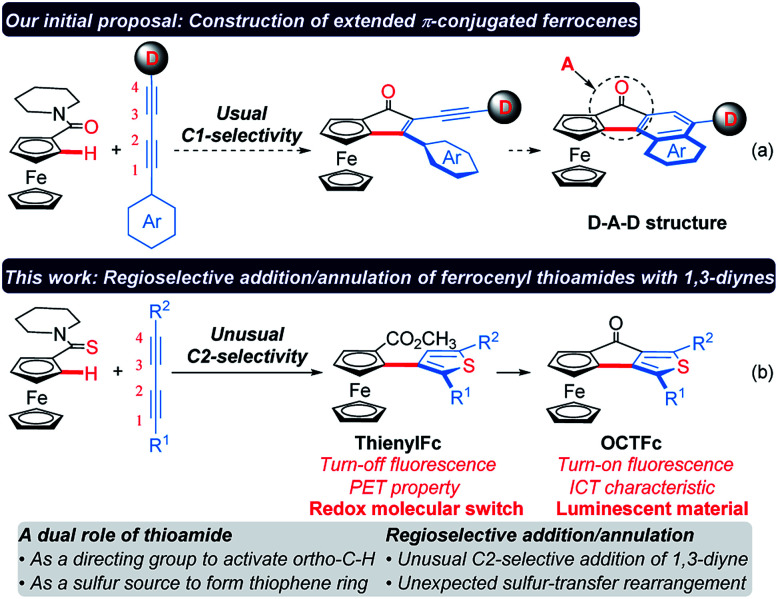
Design and synthesis of extended π-conjugated ferrocenes. D: electron donor. A: electron acceptor. PET: photoinduced electron transfer. ICT: intramolecular charge transfer.

Although the negative charge of the cyclopentadienyl (Cp) ligands endows ferrocene with electron-rich aromatic characteristics, many ferrocene derivatives are unavailable through direct electrophilic reactions, such as nitration, chlorination or bromination, because the ferrocene nucleus may be oxidized and/or destructed by nitrate ions, chlorine or bromine.^8–11^ Thus, Friedel–Crafts acylation and lithiation were proved to be the most used methods for the derivatization of ferrocene.^12–17^ However, it remains challenging to prepare 1,2-disubstituted ferrocenes because they usually require highly reactive organolithium reagents as strong bases to accomplish the *ortho*-metalation of mono-substituted ferrocenes,^14–16^ and thus inevitably suffers from poor functional group compatibility. Recently, chelating-assisted C–H bond functionalization has made significant advances,^18–25^ which provide new opportunities for the rapid construction of 1,2-disubstituted ferrocenes.^26–36^ 1,3-Diynes are an important class of organic architectures and have been widely used in the construction of various five-membered heteroaromatic rings *via* the reaction with heteroatoms.^37–40^ However, it is still a challenging task to control the regioselectivity in the migratory insertion of 1,3-diyne into the carbon–metal bond.^41^ Herein, we present a regioselective addition/annulation sequence of ferrocenyl thioamides with alkynes to construct thienylferrocene (ThienylFc) structures, which includes a rhodium-catalyzed C–H activation, an unusual C2-selective addition of 1,3-diyne, and an unexpected intramolecular sulfur-transfer rearrangement (Scheme 1b). In this protocol, thioamide not only serves as a directing group to activate the *ortho*-C–H bond of the ferrocene, but also as a sulfur source to provide the indispensable heteroatom for the formation of the thiophene ring. In addition, these resulting ThienylFc structures can be conveniently transformed into extended π-conjugated ferrocenes (4-oxocyclopentathiophene-fused ferrocenes, OCTFc) with luminescent properties *via* an intramolecular Friedel–Crafts reaction.

## Results and discussion

**Scheme 2:**
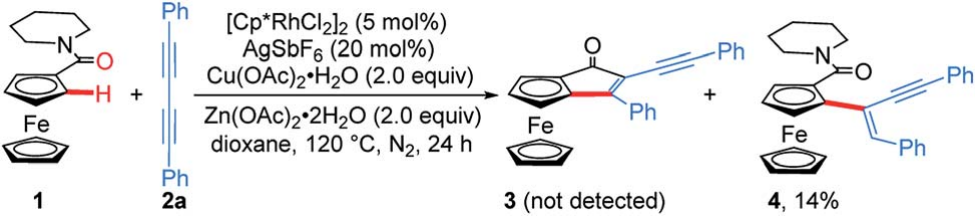
Rh-catalyzed *ortho*-C–H activation/addition of ferrocenyl amide **1** with 1,3-diyne **2a**.

**Fig. 1:**
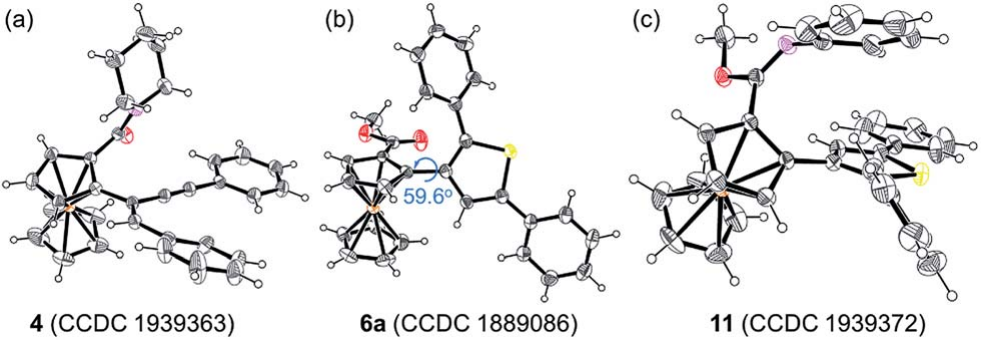
ORTEP diagrams of (a) **4**, (b) **6a,** and (c) **11**. Displacement ellipsoids are drawn at the 30% probability level.

Considering that the migratory insertion of 1,3-diyne into the carbon–metal bond usually occurs at the C1-position,^41^ we initially proposed to construct extended π-conjugated ferrocenes *via* sequential addition/annulation of ferrocenyl amide with 1,3-diyne and subsequent intramolecular annulation (Scheme 1a). Disappointingly, the reaction of ferrocenyl amide **1** with 1,3-diyne **2a** did not deliver such an annulated product **3** (Scheme 2). Actually, the analysis of X-ray single crystal diffraction demonstrated the formation of alkyne addition product **4** (Scheme 2 and Fig. 1a), which was generated *via* the migratory insertion of 1,3-diyne into the carbon–metal bond at the C2-position rather than the usual C1-position.

**Scheme 3:**
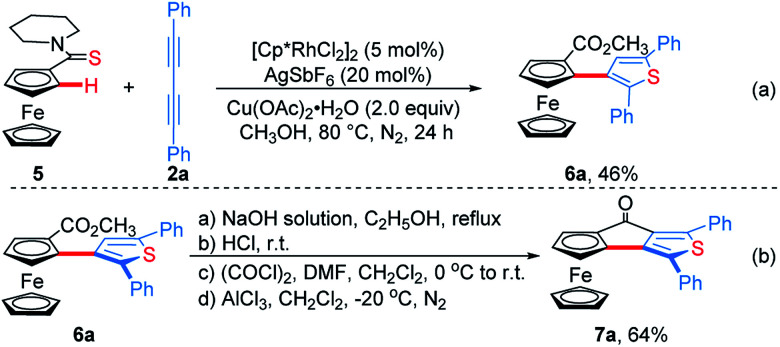
Synthesis of ThienylFc **6a** and OCTFc **7a**.

Subsequently, ferrocenyl thioamide (**5**) was attempted instead of ferrocenyl amide (**1**). Unexpectedly, in the presence of 5 mol% of [Cp*RhCl_2_]_2_, 20 mol% of AgSbF_6_ and 2.0 equiv. of Cu(OAc)_2_·H_2_O in methanol at 80 °C for 24 h, the reaction of **5** with **2a** gave ThienylFc (**6a**) in 46% yield (Scheme 3a and Table S1,† entry 1). The existence of a thiophene ring was confirmed clearly by the X-ray single crystal analysis of **6a** (Fig. 1b). In addition, **6a** could be conveniently converted into OCTFc **7a** through an intramolecular Friedel–Crafts acylation (Scheme 3b).

The reaction conditions of **5** with **2a** were further optimized. Screening of silver salts indicated that AgOTs was better than others, affording **6a** in 61% yield (Table S1,† entries 2–5). Other oxidants were proven to be less effective than Cu(OAc)_2_·H_2_O (Table S1,† entries 6–9). Owing to the poor solubility of 1,3-diyne in methanol, the solvent volume was doubled, improving the yield to 79% (Table S1,† entry 10). Moreover, the mixed solvent of trichloroethanol and methanol (1 : 3, v/v) could further improve the solubility of 1,3-diyne, delivering **6a** in an increased yield of 81% (Table S1,† entry 12). Shortening the reaction time and reducing the catalyst dosage both would decrease the yield of **6a** (Table S1,† entries 13 and 14). [Cp*Rh(CH_3_CN)_3_](OTs)_2_ was also attempted, which gave **6a** in 72% yield, demonstrating that the cationic Rh species is the real reactive catalyst of this addition/annulation of ferrocenyl thioamides with 1,3-diynes (Table S1,† entry 15).

**Scheme 4:**
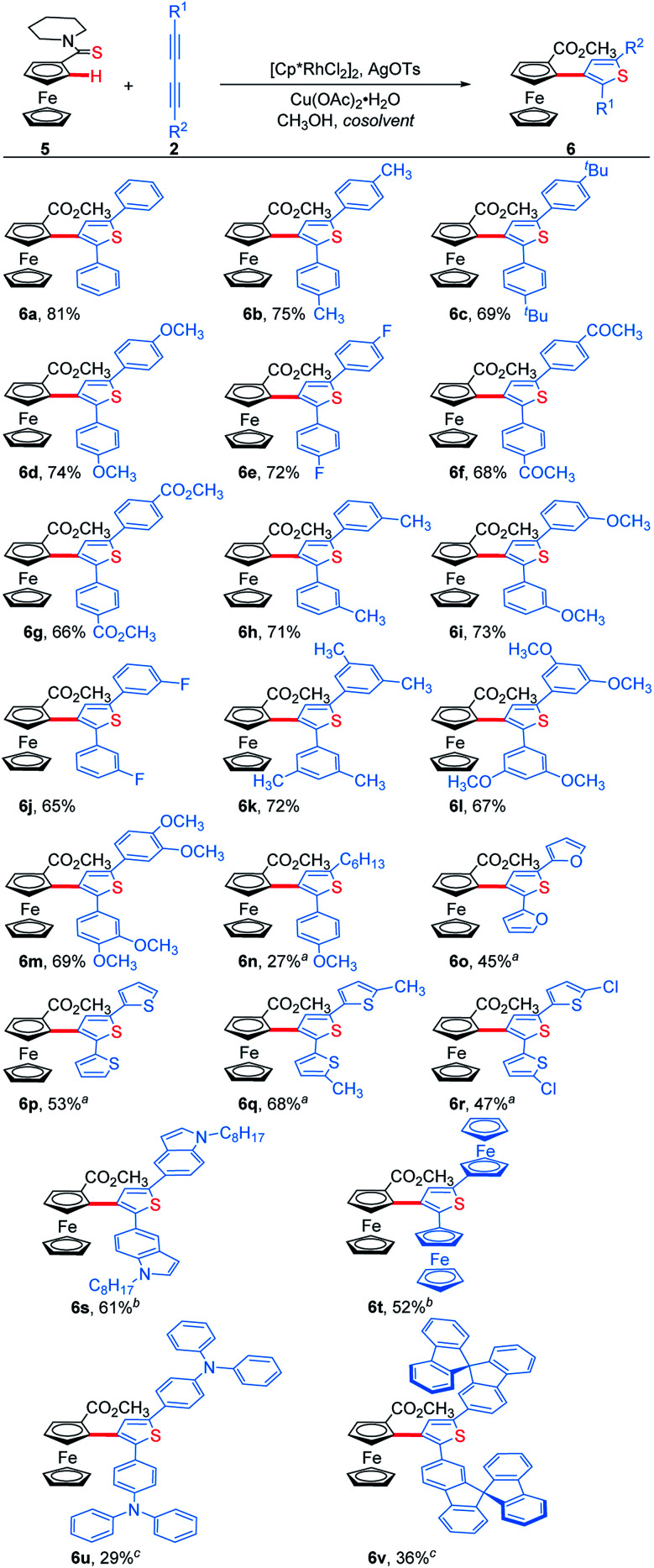
Regioselective addition/annulation of ferrocenyl thioamide with 1,3-diyne. Standard conditions: **5** (0.2 mmol), **2** (0.1 mmol), [Cp*RhCl_2_]_2_ (5 mol%), AgOTs (20 mol%), and Cu(OAc)_2_·H_2_O (2.0 equiv.) in trichloroethanol (0.5 mL)/methanol (1.5 mL) at 80 °C for 24 h under N_2_. Isolated yields. ^*a*^ Methanol (2.0 mL) as solvent. ^*b*^ 1,2-Dichloroethane (1.0 mL) and methanol (1.0 mL) as solvents. ^*c*^ 1,2-Dichloroethane (2.0 mL) and methanol (2.0 mL) as solvents at 120 °C.

We next examined the substrate scope of 1,3-diynes (Scheme 4). 1,4-Diphenyl-1,3-butadiynes with both electron-donating and electron-withdrawing substituents at the *para*- or *meta*-position of the phenyl ring afforded the desired products **6b–6j** in good yields. 3,5-Dimethyl, 3,5-dimethoxyl and 3,4-dimethoxyl phenyl-substituted 1,3-butadiynes also worked well under the standard conditions, delivering **6k**, **6l** and **6m** in 72%, 67% and 69% yields, respectively. The unsymmetrical aryl alkyl 1,3-butadiyne could undergo the addition/annulation process with complete regioselectivity confirmed with the ^1^H–^1^H NOESY spectrum, but giving a lower yield (**6n**). No product was obtained when 1,4-dialkyl-1,3-butadiynes were used as the substrate. 1,4-Di(furan-2-yl) and 1,4-di(thiophen-2-yl) substituted 1,3-butadiynes were tolerated by using methanol as solvent, giving **6o–6r** in 45–68% yields. 1,4-Di(indol-5-yl)-1,3-butadiyne and 1,4-diferrocenyl-1,3-butadiyne afforded the corresponding thienylferrocenes **6s** and **6t** in moderate yields when using 1,2-dichloroethane (DCE) as the cosolvent. In addition, ThienylFc containing special functional groups such as triphenylamine and 9,9′-spirobifluorene were prepared when doubling the volume of mixed DCE/CH_3_OH solvent and increasing the temperature to 120 °C (**6u** and **6v**).

**Scheme 5:**
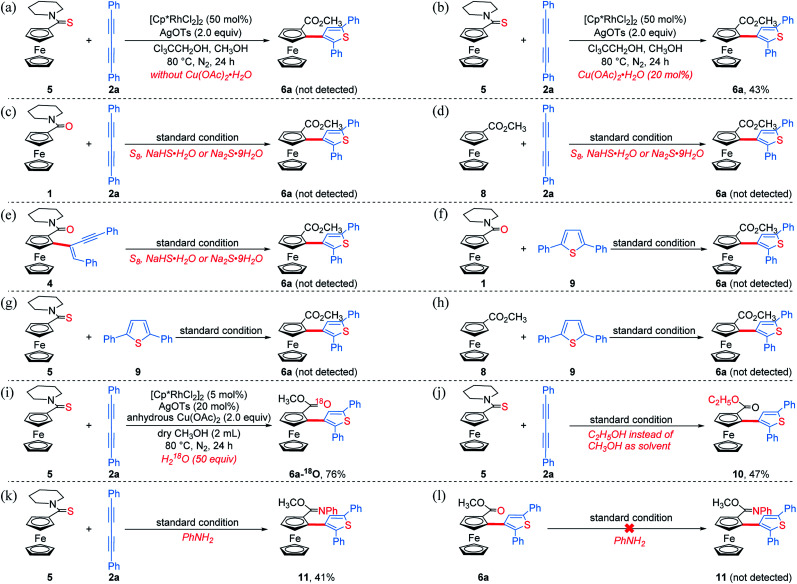
Control experiments and mechanistic investigations.

**Scheme 6:**
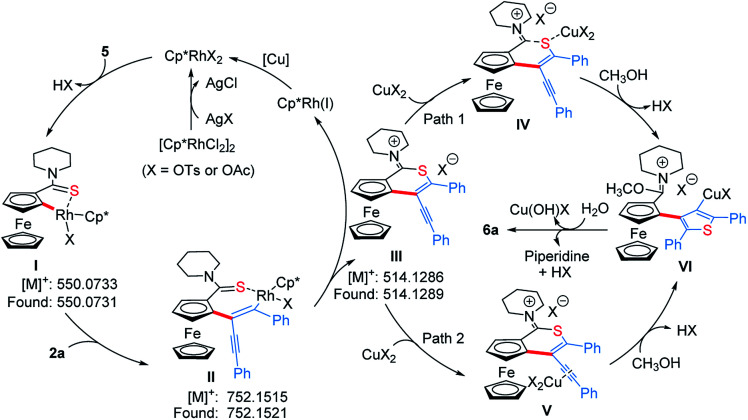
Proposed mechanistic pathway.

To get clearer insight into the pathway of the reaction relay, a series of control experiments were conducted (Scheme 5 and Section VII in the ESI†). In the absence of Cu(OAc)_2_·H_2_O, the reaction of an equivalent mole of **5** with 1,3-diyne **2a** did not provide the desired product **6a**, but **5** almost disappeared completely (Scheme 5a). Moreover, without Cu(OAc)_2_·H_2_O, **6a** could not be obtained when employing other oxidants such as AgOAc, benzoquinone, O_2_ or air (Table S1,† entries 7–9 and 16). The analysis of HRMS indicated the formation of the thiopyran intermediate **III** ([M]^+^: 514.1286, found: 514.1289) (Scheme 6 and Fig. S2†), which could not be isolated from the reaction mixture due to its poor stability. Upon addition of 20 mol% of Cu(OAc)_2_·H_2_O, **6a** was obtained in 43% yield (Scheme 5b), indicating that the sulfur-transfer reaction may need the participation of the copper ion. The coordination of Cu^2+^ with the sulfur atom or alkyne may facilitate the cleavage of the C–S bond of thioamide (Scheme 6). Considering that ferrocenyl amide (**1**) and methyl ferrocene carboxylate (**8**) could be detected under the standard reaction conditions (Scheme 4, **6a**), the reactions of **1** and **8** with **2a** were performed in the presence of external sulfur sources, such as S_8_, NaHS·H_2_O and Na_2_S·9H_2_O, respectively.^42–44^ However, **6a** was not observed (Scheme 5c and d). The cycloaddition reaction of **4** did not occur in the presence of additional sulfur sources (Scheme 5e). Moreover, **6a** was not detected in the reactions of **1**, **5** and **8** with 2,5-diphenylthiophene (**9**), respectively (Scheme 5f–h). The above results demonstrate that the sulfur atom of the thiophene ring of **6a** comes from the thioamide group *via* the intramolecular sulfur-transfer rearrangement, and moreover, the sulfur transfer is a successive process. Although thioamide has been employed as a directing group to activate *ortho*-C–H bonds,^33,45,46^ it remains undisclosed that a directing group containing sulfur simultaneously acts as a sulfur source to participate in the functionalization process.

Furthermore, **6a–18O** and ethyl thienylferrocenyl carboxylate (**10**) were formed upon addition of H_2_^18^O and ethanol into the reaction system, respectively, indicating that the carbonyl oxygen atom of **6a** stems from water and the alkoxy originates from alcohol through the nucleophilic substitution (Scheme 5i, j and Fig. S1†).^47^ When adding aniline into this reaction system, *N*-phenyl carbimidate substituted ThienylFc **11** was obtained in 41% yield (Fig. 1c and Scheme 5k). However, the reaction of **6a** and aniline could not deliver **11** under the standard conditions (Scheme 5l). The above observations further validate a successive nucleophilic substitution process.

Based on the above mechanistic studies, a plausible pathway was proposed in Scheme 6. Firstly, the thioamide-directed *ortho*-C–H activation of **5** forms the five-membered cyclic rhodium intermediate **I**, detected by ESI-HRMS ([M]^+^: 550.0733, found: 550.0731, Fig. S2†). 1,3-Diyne **2a** inserts into the C–Rh bond to generate the seven-membered cyclic rhodium intermediate **II** ([M]^+^: 752.1515, found: 752.1521, Fig. S3†). The reductive elimination of **II** delivers thiopyran **III** ([M]^+^: 514.1286, found: 514.1289, Fig. S2†), and the released Rh(i) species is re-oxidized by copper salt to the reactive Rh(iii). The coordination of the copper ion with sulfur or alkyne affords intermediate **IV** or **V**. Then, the nucleophilic attack of methanol to the imine cation and subsequent Cu(ii)-assisted sulfur-transfer form intermediate **VI**. Finally, the hydrolysis of the imidate and thienylcopper fragments gives **6a**.

**Scheme 7:**
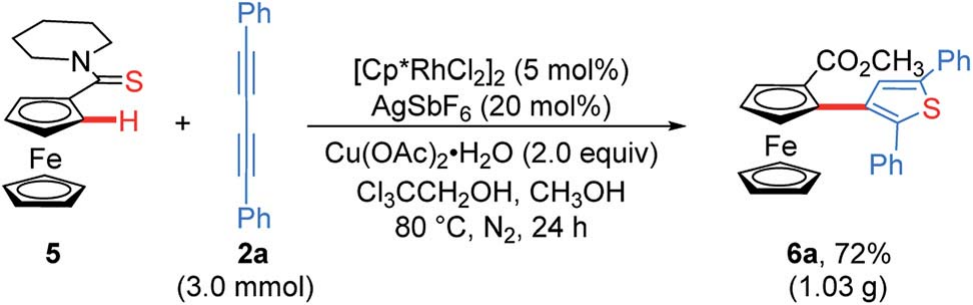
Gram-scale synthesis of ThienylFc **6a**.

**Scheme 8:**
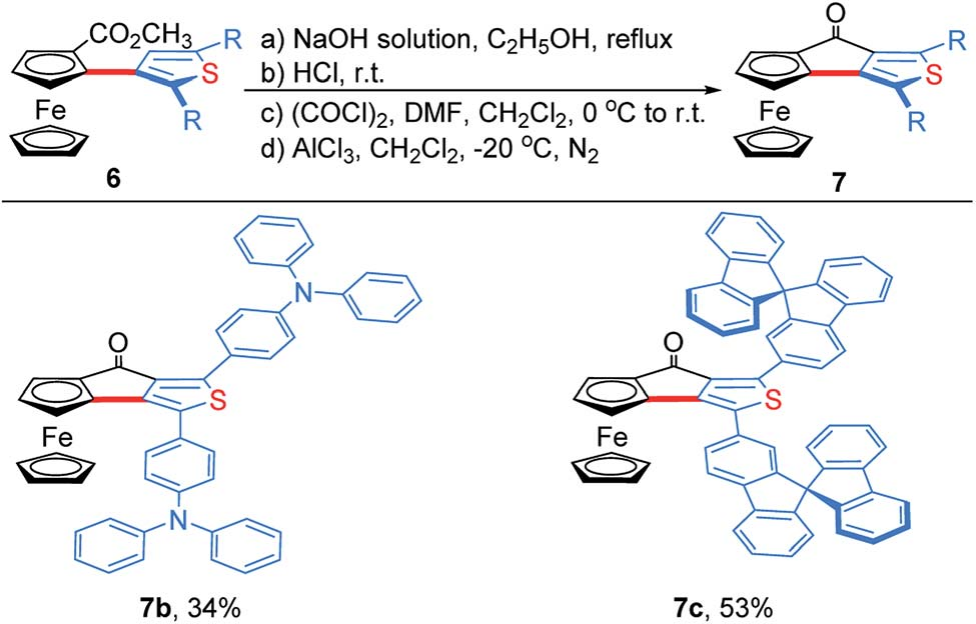
Synthesis of extended π-conjugated ferrocenes **7b** and **7c**.

To demonstrate the effectiveness and utility of our methodology, a gram scale experiment was performed, affording **6a** in 72% yield (1.03 g, Scheme 7). Subsequently, ThienylFc structures **6u** and **6v** were transformed into the extended π-conjugated OCTFc **7b** and **7c** through the hydrolysis of methyl carboxylate in NaOH solution, acidification, nucleophile substitution, and intramolecular Friedel–Crafts acylation (Scheme 8).

**Fig. 2:**
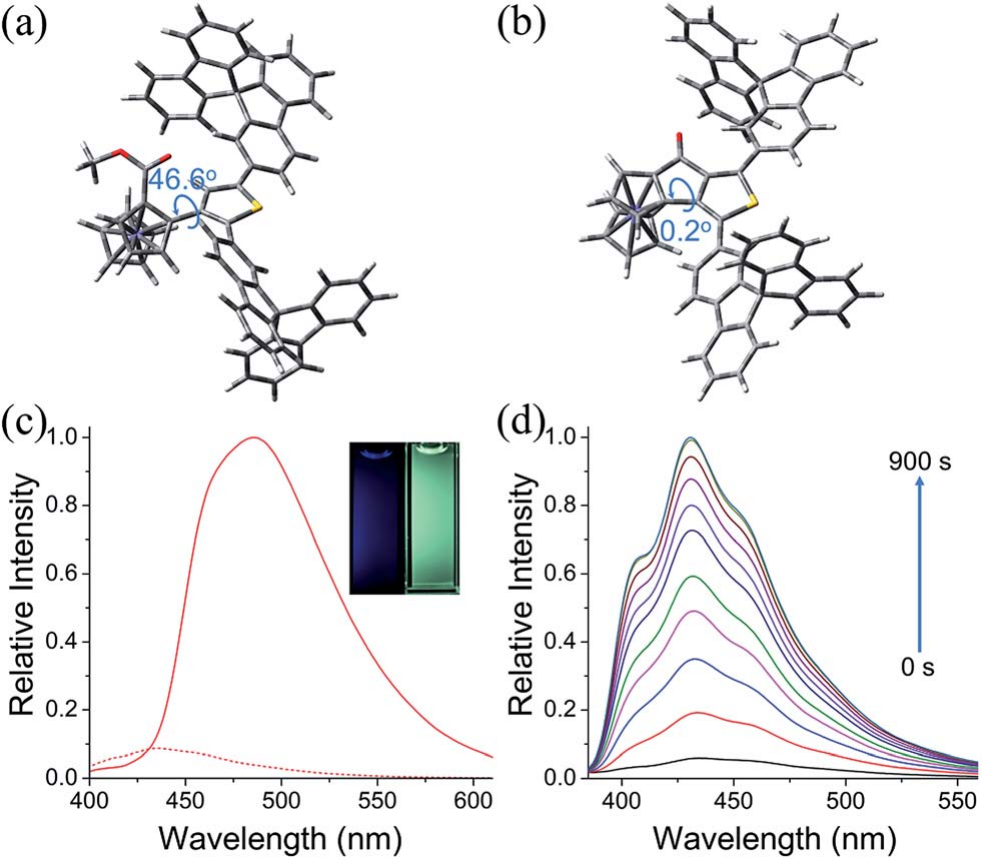
Calculated molecular conformations of (a) **6v** and (b) **7c**. Molecular optimization was performed by density functional theory (DFT) computation with Gaussian 09 at the B3LYP/6-31G* level. (c) Fluorescence spectra of **6v** (dash line) and **7c** (solid line) in CH_3_CN (1 × 10^−5^ M). Inset: fluorescence images of **6v** (left) and **7c** (right) in CH_3_CN (1 × 10^−5^ M) under UV light (365 nm). (d) Fluorescence spectra of **6v** in CH_3_CN (5 × 10^−6^ M) containing *n*-Bu_4_NPF_6_ (0.1 M) when applying an oxidation potential of +0.48 V (*vs.* Fc/Fc^+^) during 0–900 s.

The photophysical properties of **6u**, **6v**, **7b** and **7c** were studied. The absorption spectra of **6u** and **6v** show peaks at 372 nm and 361 nm, respectively (Fig. S4†). The absorption bands of **7b** and **7c** are broadened and red-shifted due to their extended π-conjugation and intramolecular charge transfer (ICT) transitions (Fig. S4†). Density functional theory (DFT) computation demonstrates that the dihedral angles between Cp and thiophene rings are 55.8° and 46.6° for the ThienylFc structures **6u** and **6v**, respectively, which are roughly consistent with that in the single-crystal data of **6a** (Fig. 1b, 2a and S5a†). The twisted conformation inhibits the ICT process. Therefore, **6u** and **6v** display effective fluorescence quenching due to the photoinduced electron transfer (PET) from the Fc unit to the excited luminophore (Fig. 2c and S6†).^5–7^ In sharp contrast, conformational twisting is not allowed in the OCTFc structure (Fig. 2b and S5b†). Thus, **7b** and **7c** exhibit relatively strong red-shifted emission owing to their ICT characteristics (Fig. 2c and S6†). The emission wavelengths of **7b** and **7c** are gradually red-shifted with an increasing solvent polarity, which are in accordance with the ICT effect (Fig. S7†).^48^ Although fused ferrocenes have been studied widely,^49–53^ the ring-fused strategy is herein employed for the first time to construct luminescent materials based on ferrocenes, which would give us inspiration for the development of novel organic optoelectronic materials, such as electroluminescent materials based on ferrocenes.

The fluorescence on/off properties of **6v** in an electro-redox process was investigated (Fig. 2d, S8 and S9†). To the CH_3_CN solution (5 × 10^−6^ M) of **6v** with *n*-Bu_4_NPF_6_ (0.1 M) as the supporting electrolyte, an oxidation potential of +0.48 V (*vs.* Fc/Fc^+^) was applied. As shown in Fig. 2d, the fluorescence intensity of **6v** exhibits an obviously upward trend with increasing time of electrochemical oxidation. Emission enhancement of approximately 17-fold is observed when applying the oxidation potential for 900 s. When applying a reduction potential of −0.62 V (*vs.* Fc/Fc^+^) to the oxidized solution of **6v**, the emission intensity decreases gradually to the initial low fluorescence intensity. Moreover, the oxidation and reduction of **6v** were carried out for several cycles without significant fatigue (Fig. S9†). These results show that **6v** is a promising redox molecular switch.

## Conclusions

In summary, we have developed a highly efficient strategy to construct ThienylFc structures *via ortho*-C–H activation/addition/annulation of ferrocenyl thioamides with 1,3-diynes. Mechanistic studies demonstrate that the formation of the thiophene ring is a successive process *via* the addition reaction of the sulfur atom of the thiocarbonyl group to the C1-position of 1,3-diyne and subsequent intramolecular sulfur-transfer rearrangement from the carbonyl group to the C4-position of 1,3-diyne. In this protocol, thioamide plays a dual role, firstly, as a directing group to activate *ortho*-C–H on ferrocene, and secondly, as a sulfur source to form the thiophene ring. The resulting ThienylFc can be conveniently transformed into extended π-conjugated ferrocenes with luminescent properties through the sequential hydrolysis and intramolecular Friedel–Crafts acylation. ThienylFc displays effective fluorescence quenching due to the PET process from the Fc unit to the excited luminophore, while OCTFc exhibits relatively strong emission owing to their ICT characteristics. In this work, the ring-fused strategy is employed for the first time to construct luminescent materials based on ferrocenes, which provides inspiration for the development of novel organic optoelectronic materials, such as electroluminescent materials based on ferrocenes.

## Conflicts of interest

There are no conflicts to declare.

## Supplementary Material

SC-011-D0SC04597G-s001

SC-011-D0SC04597G-s002
