# Supplementary material for: Regioselective addition/annulation of ferrocenyl thioamides with 1,3-diynes via a sulfur-transfer rearrangement to construct extended π-conjugated ferrocenes with luminescent properties
Source: Chem Sci. 2020 Sep 16;11(40):11030–6. doi: 10.1039/d0sc04597g (PMC8162306; doi:10.1039/d0sc04597g)
Supplement: SC-011-D0SC04597G-s001 [file SC-011-D0SC04597G-s001.pdf]

Electronic Supplementary Material (ESI) for Chemical Science.
This journal is © The Royal Society of Chemistry 2020

Supplementary Information

# Regioselective addition/annulation of ferrocenyl thioamides with 1,3-diynes *via* a sulfur-transfer rearrangement to construct extended π-conjugated ferrocenes with luminescent property

Lipeng Yan, Jingbo Lan,* Hu Cheng, Yihang Li, Mangang Zhang, Di Wu, and Jingsong You*

*Key Laboratory of Green Chemistry and Technology of Ministry of Education, College of Chemistry, Sichuan University, 29 Wangjiang Road, Chengdu 610064, People's Republic of China*

# Table of Contents

I. General Remarks ........ S3

II. Synthesis of 1,3-diynes **2** ........ S4

III. Rh catalyzed *ortho*-C–H activation/addition of ferrocenyl amide **1** with 1,3-diyne **2a** ........ S6

IV. Optimization of regioselective addition/annulation of ferrocenyl thioamide **5** with 1,3-diyne **2a** ........ S7

V. General procedure for regioselective addition/annulation of ferrocenyl thioamide **5** with 1,3-diynes **2** ........ S9

VI. Gram-scale synthesis of **6a** ........ S9

VII. Mechanistic studies ........ S10

VIII. Synthesis of extended π-conjugated ferrocenes **7** ........ S16

IX. Calculated molecular conformations, photophysical and electrochemical properties ........ S17

X. Preparation and characterization of the described compounds ........ S19

XI. References ........ S36

XII. Copies of $^{1}H$, $^{13}C$ and $^{1}H$-$^{1}H$ NOESY NMR spectra ........ S37

## I. General Remarks

Unless otherwise noted, all reagents were obtained from commercial suppliers and used without further purification. Ferrocenyl amide **1**,[1] ferrocenyl thioamide **5**,[1] methyl ferrocene carboxylate **8**,[2] and 2,5-diphenylthiophene **9**[3] and 1-(deca-1,3-diyn-1-yl)-4-methoxybenzene **2n**[4] were prepared according to literature procedures. 1,3-Diynes **2a**-**2e** and **2h-2j** were prepared by homocouplings of the corresponding terminal alkynes.[5] 1,3-Diynes **2f**, **2g**, **2k**-**2m** and **2o**-**2r** were prepared *via* the Sonogashira coupling reaction of the corresponding brominated (hetero)arenes with trimethylsilylacetylene, desilication, and homocoupling of the resulting terminal alkynes.[5]

NMR spectra were obtained on an Agilent 400-MR DD2 spectrometer. $^{1}$H NMR (400 MHz) chemical shifts were measured relative to $CDCl_3$ or DMSO-$d_6$ as the internal reference ($CDCl_3$: $\delta$ = 7.26 ppm; DMSO-$d_6$: $\delta$ = 2.50 ppm). $^{13}$C NMR (100 MHz) chemical shifts were measured relative to $CDCl_3$ or DMSO-$d_6$ as the internal reference ($CDCl_3$: $\delta$ = 77.16 ppm; DMSO-$d_6$: $\delta$ = 39.52 ppm). High-resolution mass spectra (HRMS) were obtained with a Shimadzu LCMS-IT-TOF (ESI) spectrometer. Melting points were determined with XRC-1 with no correction. The absorption and fluorescence spectra were obtained using a HITACHI U-2910 spectrometer and a Horiba Fluorolog-3 fluorescence spectrometer, respectively.

Cyclic voltammetry (CV) measurements were carried out on a CHI660D electrochemical workstation at a scan rate of 50 mV/s in dry acetonitrile containing 0.1 M of *n*-$Bu_4NPF_6$ supporting electrolyte under an $N_2$ atmosphere, using Fc/$Fc^+$ (0.08 V) as reference. The three-electrode setup consisted of an Ag/$Ag^+$ (0.01 M of $AgNO_3$ in acetonitrile) reference electrode, a platinum wire counter electrode, and a platinum plate working electrode.

Spectroelectrochemical experiments were performed in a thin-layer (1 mm) quartz cell under an $N_2$ atmosphere, employing dry acetonitrile as solvent with *n*-$Bu_4NPF_6$ (0.1 M) as supporting electrolyte. The three-electrode setup consisted of a platinum

network working electrode, a platinum wire counter electrode, and an Ag/AgCl reference electrode (0.52 V, vs Fc/Fc$^+$).

## II. Synthesis of 1,3-diynes 2

### 2.1 Synthesis of 1,3-diyne 2s[5,6]

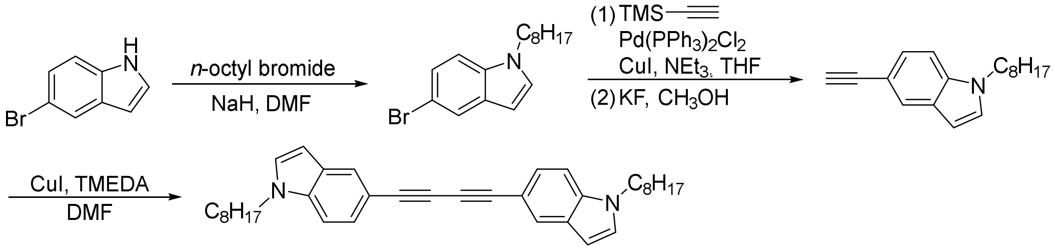


**2s**

A solution of 5-bromoindole (0.98 g, 5 mmol) in anhydrous DMF (15 mL) was added dropwise over 20 min to a stirred suspension of NaH (60% dispersion in mineral oil, 0.32 g, 8 mmol) in anhydrous DMF (15 mL) in an ice bath. A solution of *n*-octyl bromide (0.9 mL, 5.2 mmol) in anhydrous DMF (15 mL) was slowly added over 5 min. Then, the mixture was warmed to room temperature and stirred overnight. The reaction was quenched with ethanol (10 mL) carefully and poured into ice-water. The resulting mixture was extracted with dichloromethane twice. The combined organic layer was washed with brine, dried with anhydrous $Na_2SO_4$, and concentrated under reduced pressure. The residue was purified by column chromatography on silica gel (petroleum ether/ethyl acetate = 20/1, v/v) to give 5-bromo-1-octyl-1*H*-indole as a yellow oil in 87% yield (1.34 g).

A three-necked flask with a magnetic stir bar was charged with 5-bromo-1-octyl-1*H*-indole (1.23 g, 4 mmol), trimethylsilylacetylene (1.1 mL, 8 mmol), $Pd(PPh_3)_2Cl_2$ (140.4 mg, 5 mol%), CuI (76.2 mg, 10 mol%), triethylamine (3 mL) and THF (9 mL) under an $N_2$ atmosphere. After stirred at 80 °C for 24 h, the resulting mixture was cooled to room temperature. Then, KF (0.46 g, 8 mmol) was added along with $CH_3OH$ (10 mL), and the mixture was stirred under air for another 4 h. The mixture was filtered through a celite pad and washed with 20 mL of dichloromethane. Then the filtrate was concentrated under reduced pressure and purified by column

chromatography on silica gel (petroleum ether/ethyl acetate = 20/1, v/v) to provide 5-ethynyl-1-octyl-1*H*-indole as a yellow oil in 79% yield (0.80 g).

CuI (38.1 mg, 10 mol%) and TMEDA (0.3 mL, 2 mmol) was added to the solution of 5-ethynyl-1-octyl-1*H*-indole (0.51 g, 2 mmol) in DMF (5 mL). The resulting mixture was then stirred at room temperature overnight. Then, the mixture was diluted in ethyl acetate (50 mL). The organic layer was washed with saline, dried over $MgSO_4$, filtered and the filtrate was concentrated under reduced pressure. The residue was then purified by column chromatography on silica gel (petroleum ether/ethyl acetate = 20/1, v/v) to give 1,4-bis(1-octyl-1*H*-indol-5-yl)buta-1,3-diyne (**2s**) as a yellow oil in a yield of 81% (0.41 g).

### 2.2 Synthesis of 1,3-diyne 2t[5,7]

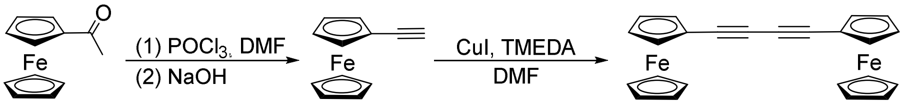


**2t**

Acetyl ferrocene (1.14 g, 5 mmol) was dissolved in DMF (15 mL) and then phosphorus oxychloride (3 mL) in DMF (10 mL) was added dropwise at 0 °C under $N_2$. The reaction mixture was stirred at 0 °C for 15 min and then warmed to room temperature for 2 h. The mixture was poured to 20% of NaOAc solution (100 mL) under $N_2$ and stirred for 1.5 h. The mixture was extracted with dichloromethane and washed with saline. The organic phase was dried over $MgSO_4$, filtered and the filtrate was concentrated under reduced pressure to give the crude product. To the solution of the crude product in 1,4-dioxane (8 mL) under $N_2$, 0.5 M of NaOH solution (5 mL) was added. Then the reaction mixture was refluxed for 5 min and poured into ice-water (50 mL). The mixture was subsequently neutralized by 1 N of HCl solution and extracted with ethyl acetate. The organic layer was washed with saline, dried over $MgSO_4$, filtered and the filtrate was concentrated under reduced pressure. The crude product was purified by column chromatography on silica gel (petroleum ether/ethyl acetate = 20/1, v/v) to give ethynyl ferrocene in a yield of 61% (0.64 g).

CuI (38.1 mg, 10 mol%) and TMEDA (0.3 mL, 2 mmol) was added to the solution of ethynyl ferrocene (0.42 g, 2 mmol) in DMF (5 mL). The resulting mixture was then stirred at room temperature overnight. Then, the mixture was diluted in ethyl acetate (50 mL). The organic layer was washed with saline, dried over $MgSO_4$, filtered and the filtrate was concentrated under reduced pressure. The residue was then purified by column chromatography on silica gel (petroleum ether/ethyl acetate = 20/1, v/v) to give 1,4-diferrocenylbuta-1,3-diyne (**2t**) as a red solid in a yield of 67% (0.28 g).

### 2.3 Synthesis of 1,3-diyne 2u and 2v[5]

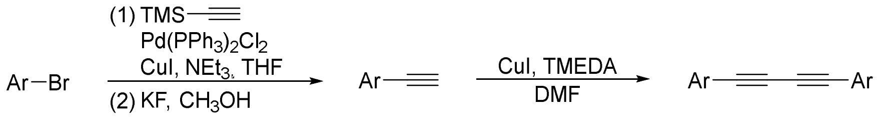


A three-necked flask with a magnetic stir bar was charged with the brominated arenes (5 mmol), trimethylsilylacetylene (10 mmol), $Pd(PPh_3)_2Cl_2$ (5 mol%), CuI (10 mol%), triethylamine (3 mL) and THF (9 mL) under an $N_2$ atmosphere. After refluxed at 80 °C for 24 h, the resulting mixture was cooled to room temperature. Then, KF (10 mmol) was added along with $CH_3OH$ (10 mL), and the mixture was stirred under air for another 4 h. The mixture was filtered through a celite pad and washed with 20 mL of dichloromethane. Then the filtrate was concentrated under reduced pressure and purified by column chromatography on silica gel to provide the corresponding terminal alkynes.

CuI (10 mol%) and TMEDA (2 mmol) was added to the solution of the acquired terminal alkyne (2 mmol) in DMF (5 mL). The resulting mixture was then stirred at room temperature overnight. Then, the mixture was diluted in ethyl acetate (50 mL). The organic layer was washed with saline, dried over $MgSO_4$, filtered and the filtrate was concentrated under reduced pressure. The residue was then purified by column chromatography on silica gel to give the final 1,3-diyne products.

## III. Rh catalyzed *ortho*-C–H activation/addition of ferrocenyl amide 1

**with 1,3-diyne 2a**

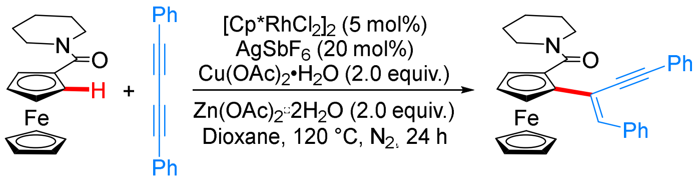


A 25 mL Schlenk sealed tube with a magnetic stir bar was charged with ferrocenyl amides **1** (59.4 mg, 0.2 mmol), 1,4-diphenylbuta-1,3-diyne **2a** (40.5 mg, 0.2 mmol, 1.0 equiv.), $[Cp^*RhCl_2]_2$ (6.2 mg, 5 mol%), $AgSbF_6$ (13.7 mg, 20 mol%), $Cu(OAc)_2{\cdot}H_2O$ (79.9 mg, 0.4 mmol, 2.0 equiv.), $Zn(OAc)_2{\cdot}2H_2O$ (87.8 mg, 0.4 mmol, 2.0 equiv.) and 1,4-dioxane (2 mL) under an $N_2$ atmosphere. The resulting mixture was stirred at 120 °C for 24 h and then diluted with 10 mL of dichloromethane. The mixture was filtered through a celite pad and washed with 20 mL of dichloromethane. Then the filtrate was concentrated under reduced pressure and purified by column chromatography on silica gel (petroleum ether/ethyl acetate = 4/1, v/v) to provide compound **4** as a yellow solid in a yield of 14% (14.2 mg).

## IV. Optimization of regioselective addition/annulation of ferrocenyl thioamide 5 with 1,3-diyne 2a

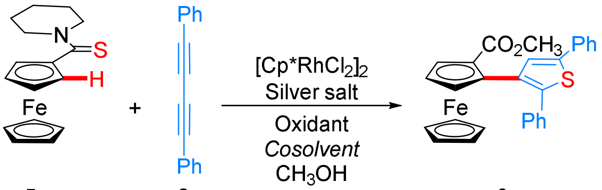


A 25 mL Schlenk sealed tube with a magnetic stir bar was charged with ferrocenyl thioamide **5** (62.6 mg, 0.2 mmol, 2.0 equiv.), 1,4-diphenyl-1,3-butadiyne **2a** (20.2 mg, 0.1 mmol), [Rh], [Ag], oxidant and solvent under an $N_2$ atmosphere. The resulting mixture was stirred at 80 °C for 16-24 h and then diluted with 10 mL of dichloromethane. The mixture was filtered through a celite pad and washed with 20 mL of dichloromethane. Then the filtrate was concentrated under reduced pressure.

The residue was dissolved in $CDCl_3$ and analyzed by $^1H$ NMR with $CH_2Br_2$ (3.5 $\mu$L, 0.05 mmol) as the internal standard. The NMR yields determined by the characteristic proton signal of **6a** at 4.88 ppm relative to the internal standard signal of $CH_2Br_2$ at 4.93 ppm. If necessary, the residue would be purified by column chromatography on silica gel (petroleum ether/ethyl acetate = 15/1, v/v) to give the isolated yield of **6a**.

**Table S1. Optimization of regioselective addition/annulation of 1,3-diyne 2a with ferrocenyl thioamide 5.**[a]

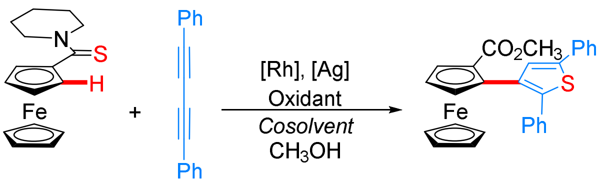


5 2a 6a

| Entry | [Rh] | [Ag] | Oxidant | Solvent | Yield (%)[b] |
|---|---|---|---|---|---|
| 1 | $[Cp^*RhCl_2]_2$ | $AgSbF_6$ | $Cu(OAc)_2{\cdot}H_2O$ | $CH_3OH$ | 46[c] |
| 2 | $[Cp^*RhCl_2]_2$ | $AgBF_4$ | $Cu(OAc)_2{\cdot}H_2O$ | $CH_3OH$ | 49 |
| 3 | $[Cp^*RhCl_2]_2$ | AgOTf | $Cu(OAc)_2{\cdot}H_2O$ | $CH_3OH$ | 38 |
| 4 | $[Cp^*RhCl_2]_2$ | $AgNTf_2$ | $Cu(OAc)_2{\cdot}H_2O$ | $CH_3OH$ | 55 |
| 5 | $[Cp^*RhCl_2]_2$ | AgOTs | $Cu(OAc)_2{\cdot}H_2O$ | $CH_3OH$ | 61 |
| 6 | $[Cp^*RhCl_2]_2$ | AgOTs | $Cu(OPiv)_2$ | $CH_3OH$ | 37 |
| 7 | $[Cp^*RhCl_2]_2$ | AgOTs | AgOAc | $CH_3OH$ | trace |
| 8 | $[Cp^*RhCl_2]_2$ | AgOTs | Oxygen (1 atm) | $CH_3OH$ | N.D. |
| 9 | $[Cp^*RhCl_2]_2$ | AgOTs | Benzoquinone | $CH_3OH$ | N.D. |
| 10[d] | $[Cp^*RhCl_2]_2$ | AgOTs | $Cu(OAc)_2{\cdot}H_2O$ | $CH_3OH$ | 79 |
| 11[e] | $[Cp^*RhCl_2]_2$ | AgOTs | $Cu(OAc)_2{\cdot}H_2O$ | DCE/$CH_3OH$ | 65 |
| 12[f] | $[Cp^*RhCl_2]_2$ | AgOTs | $Cu(OAc)_2{\cdot}H_2O$ | $Cl_3CCH_2OH/CH_3OH$ | 83 (81[c]) |
| 13[g] | $[Cp^*RhCl_2]_2$ | AgOTs | $Cu(OAc)_2{\cdot}H_2O$ | $Cl_3CCH_2OH/CH_3OH$ | 69 |
| 14[h] | $[Cp^*RhCl_2]_2$ | AgOTs | $Cu(OAc)_2{\cdot}H_2O$ | $Cl_3CCH_2OH/CH_3OH$ | 47 |
| 15[i] | $[Cp^*Rh(CH_3CN)_3](OTs)_2$ | – | $Cu(OAc)_2{\cdot}H_2O$ | $Cl_3CCH_2OH/CH_3OH$ | 72 |
| 16[j] | $[Cp^*Rh(CH_3CN)_3](OTs)_2$ | – | Air (1 atm) | $Cl_3CCH_2OH/CH_3OH$ | N.D. |

[a]Reactions were carried out by using **5** (0.2 mmol), **2a** (0.1 mmol), [Rh] (5 mol%), [Ag] (20 mol%) and oxidant (2.0 equiv.) in $CH_3OH$ (1 mL) at 80 °C for 24 h under an $N_2$ atmosphere. [b]NMR yields. [c]Isolated yields. [d]$CH_3OH$ (2 mL). [e]1,2-Dichloroethane (DCE, 0.5 mL) and $CH_3OH$ (1.5 mL). [f]$Cl_3CCH_2OH$ (0.5 mL) and $CH_3OH$ (1.5 mL). [g]$Cl_3CCH_2OH$ (0.5 mL), $CH_3OH$ (1.5 mL) and stirred for 16 h. [h][Cp*RhCl$_2$]$_2$ (2.5 mol%), AgOTs (10 mol%), $Cl_3CCH_2OH$ (0.5 mL) and $CH_3OH$ (1.5 mL). [i][Cp*Rh($CH_3CN$)$_3$](OTs)$_2$ (10 mol%), $Cl_3CCH_2OH$ (0.5 mL) and $CH_3OH$ (1.5 mL). [j][Cp*Rh($CH_3CN$)$_3$](OTs)$_2$ (10 mol%), $Cl_3CCH_2OH$ (0.5 mL) and $CH_3OH$ (1.5 mL) under air. N.D. = not detected.

## V. General procedure for regioselective addition/annulation of ferrocenyl thioamide 5 with 1,3-diynes 2

A 25 mL Schlenk sealed tube with a magnetic stir bar was charged with ferrocenyl thioamide **5** (0.2 mmol), 1,3-diyne **2** (0.1 mmol), [Cp*RhCl$_2$]$_2$ (5 mol%), AgOTs (20 mol%), $Cu(OAc)_2·H_2O$ (0.2 mmol), $Cl_3CCH_2OH$ (0.5 mL) and $CH_3OH$ (1.5 mL) under an $N_2$ atmosphere. The resulting mixture was stirred at 80 °C for 24 h and then diluted with 10 mL of dichloromethane. The mixture was filtered through a celite pad and washed with about 20 mL of dichloromethane. Then the filtrate was concentrated under reduced pressure and purified by column chromatography on silica gel to provide desired products **6**.

## VI. Gram-scale synthesis of 6a

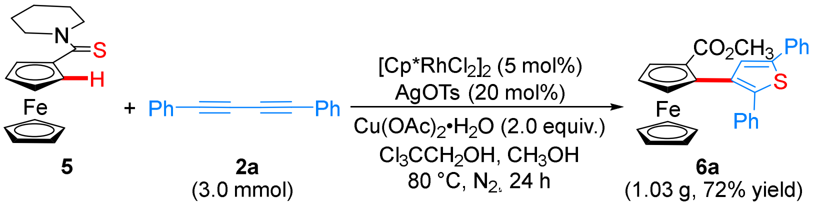


A 100 mL three-necked flask with a magnetic stir bar was charged with ferrocenyl thioamides **5** (1.88 g, 6.0 mmol, 2.0 equiv.), 1,4-diphenylbuta-1,3-diyne **2a** (0.61 g, 3.0 mmol), [Cp*RhCl$_2$]$_2$ (92.7 mg, 5 mol%), AgOTs (167.4 mg, 20 mol%), $Cu(OAc)_2·H_2O$ (1.20 g, 6.0 mmol, 2.0 equiv.), $Cl_3CCH_2OH$ (7.5 mL) and $CH_3OH$ (22.5 mL) under an $N_2$ atmosphere. The resulting mixture was stirred at 80 °C for 24 h and then diluted with 20 mL of dichloromethane. The mixture was filtered through a celite pad and washed with 30 mL of dichloromethane. Then the filtrate was concentrated under reduced

pressure and purified by column chromatography on silica gel (petroleum ether/ethyl acetate = 15:1, v/v) to provide the desired product **6a** (1.03 g, 72% yield).

## VII. Mechanistic studies

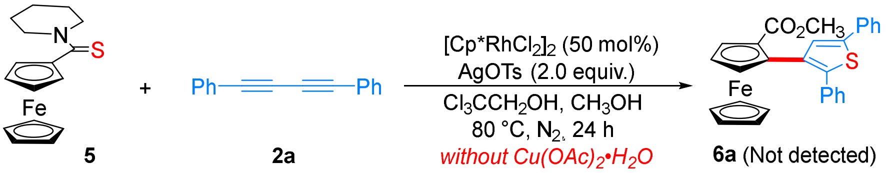


A 25 mL Schlenk sealed tube with a magnetic stir bar was charged with ferrocenyl thioamide **5** (31.3 mg, 0.1 mmol), 1,4-diphenylbuta-1,3-diyne **2a** (20.2 mg, 0.1 mmol), $[Cp^*RhCl_2]_2$ (30.9 mg, 0.05 mmol, 50 mol%), AgOTs (55.8 mg, 0.2 mmol, 2.0 equiv.), $Cl_3CCH_2OH$ (0.5 mL) and $CH_3OH$ (1.5 mL) under an $N_2$ atmosphere. The resulting mixture was stirred at 80 °C for 24 h and then diluted with 10 mL of dichloromethane. The mixture was filtered through a celite pad and washed with 20 mL of dichloromethane. The filtrate was concentrated under reduced pressure, followed by dissolved in $CDCl_3$. The in-situ $^1H$ NMR spectrum of the reaction mixture showed that there was no product **6a** obtained.

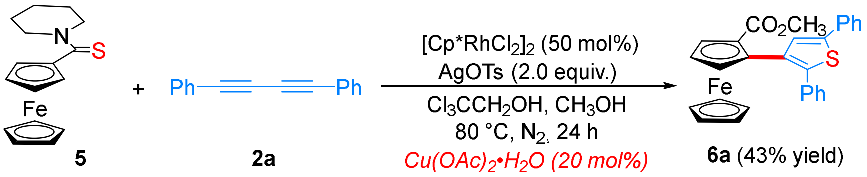


A 25 mL Schlenk sealed tube with a magnetic stir bar was charged with ferrocenyl thioamide **5** (31.3 mg, 0.1 mmol), 1,4-diphenylbuta-1,3-diyne **2a** (20.2 mg, 0.1 mmol), $[Cp^*RhCl_2]_2$ (30.9 mg, 0.05 mmol, 50 mol%), AgOTs (55.8 mg, 0.2 mmol, 2.0 equiv.), $Cu(OAc)_2 \cdot H_2O$ (4.0 mg, 0.02 mmol, 20 mol%), $Cl_3CCH_2OH$ (0.5 mL) and $CH_3OH$ (1.5 mL) under an $N_2$ atmosphere. The resulting mixture was stirred at 80 °C for 24 h and then diluted with 10 mL of dichloromethane. The mixture was filtered through a celite pad and washed with 20 mL of dichloromethane. The filtrate was concentrated under reduced pressure and purified by column chromatography on silica gel (petroleum ether/ethyl acetate = 15:1, v/v), providing compound **6a** with 43% yield (20.6 mg).

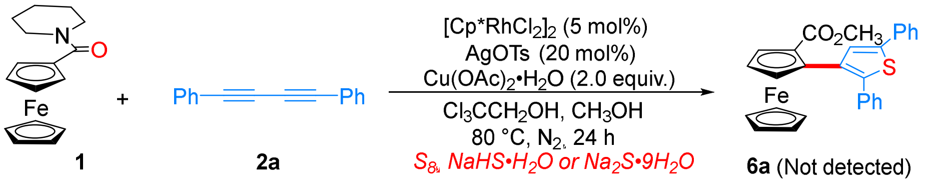


A 25 mL Schlenk sealed tube with a magnetic stir bar was charged with ferrocenyl amide **1** (59.4 mg, 0.2 mmol, 2.0 equiv.), 1,4-diphenylbuta-1,3-diyne **2a** (20.2 mg, 0.1 mmol), the corresponding external sulfur source (0.3 mmol, 3.0 equiv.), $[Cp^*RhCl_2]_2$ (3.1 mg, 5 mol%), AgOTs (5.6 mg, 20 mol%), $Cu(OAc)_2 \cdot H_2O$ (39.9 mg, 0.2 mmol, 2.0 equiv.), $Cl_3CCH_2OH$ (0.5 mL) and $CH_3OH$ (1.5 mL) under an $N_2$ atmosphere. The resulting mixture was stirred at 80 °C for 24 h and then diluted with 10 mL of dichloromethane. The mixture was filtered through a celite pad and washed with 20 mL of dichloromethane. The filtrate was concentrated under reduced pressure, followed by dissolved in $CDCl_3$. In-situ $^1H$ NMR spectra of all the reaction mixtures showed that there was no product **6a** obtained.

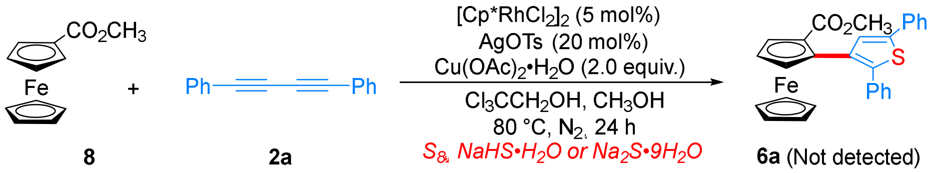


A 25 mL Schlenk sealed tube with a magnetic stir bar was charged with methyl ferrocene carboxylate **8** (48.8 mg, 0.2 mmol, 2.0 equiv.), 1,4-diphenylbuta-1,3-diyne **2a** (20.2 mg, 0.1 mmol), the corresponding external sulfur source (0.3 mmol, 3.0 equiv.), $[Cp^*RhCl_2]_2$ (3.1 mg, 5 mol%), AgOTs (5.6 mg, 20 mol%), $Cu(OAc)_2 \cdot H_2O$ (39.9 mg, 0.2 mmol, 2.0 equiv.), $Cl_3CCH_2OH$ (0.5 mL) and $CH_3OH$ (1.5 mL) under an $N_2$ atmosphere. The resulting mixture was stirred at 80 °C for 24 h and then diluted with 10 mL of dichloromethane. The mixture was filtered through a celite pad and washed with 20 mL of dichloromethane. The filtrate was concentrated under reduced pressure, followed by dissolved in $CDCl_3$. In-situ $^1H$ NMR spectra of all the reaction mixtures showed that there was no product **6a** obtained.

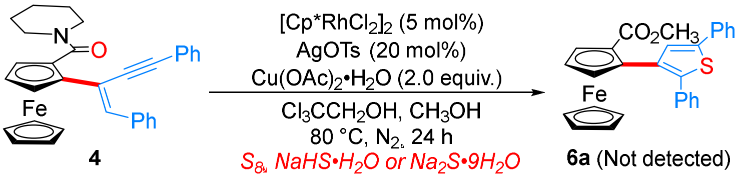


A 25 mL Schlenk sealed tube with a magnetic stir bar was charged with **4** (49.9 mg, 0.1 mmol), the corresponding external sulfur source (0.3 mmol, 3.0 equiv.), $[Cp^*RhCl_2]_2$ (3.1 mg, 5 mol%), AgOTs (5.6 mg, 20 mol%), $Cu(OAc)_2\cdot H_2O$ (39.9 mg, 0.2 mmol, 2.0 equiv.), $Cl_3CCH_2OH$ (0.5 mL) and $CH_3OH$ (1.5 mL) under an $N_2$ atmosphere. The resulting mixture was stirred at 80 °C for 24 h and then diluted with 10 mL of dichloromethane. The mixture was filtered through a celite pad and washed with 20 mL of dichloromethane. The filtrate was concentrated under reduced pressure, followed by dissolved in $CDCl_3$. The in-situ $^1H$ NMR spectrum of the reaction mixture showed that there was no product **6a** obtained.

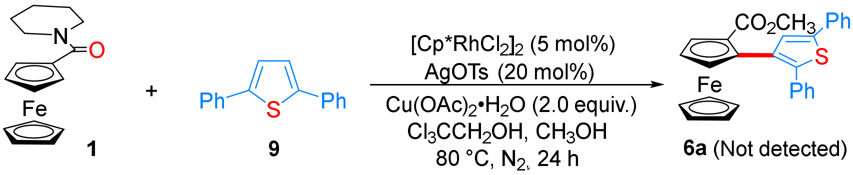


A 25 mL Schlenk sealed tube with a magnetic stir bar was charged with ferrocenyl amide **1** (59.4 mg, 0.2 mmol, 2.0 equiv.), 2,5-diphenylthiophene **9** (23.6 mg, 0.1 mmol), $[Cp^*RhCl_2]_2$ (3.1 mg, 5 mol%), AgOTs (5.6 mg, 20 mol%), $Cu(OAc)_2\cdot H_2O$ (39.9 mg, 0.2 mmol, 2.0 equiv.), $Cl_3CCH_2OH$ (0.5 mL) and $CH_3OH$ (1.5 mL) under an $N_2$ atmosphere. The resulting mixture was stirred at 80 °C for 24 h and then diluted with 10 mL of dichloromethane. The mixture was filtered through a celite pad and washed with 20 mL of dichloromethane. The filtrate was concentrated under reduced pressure, followed by dissolved in $CDCl_3$. In-situ $^1H$ NMR spectra of all the reaction mixtures showed that there was no product **6a** obtained.

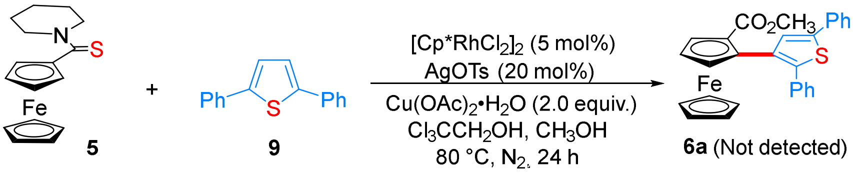

A 25 mL Schlenk sealed tube with a magnetic stir bar was charged with ferrocenyl thioamide **5** (62.6 mg, 0.2 mmol, 2.0 equiv.), 2,5-diphenylthiophene **9** (23.6 mg, 0.1 mmol), $[Cp^*RhCl_2]_2$ (3.1 mg, 5 mol%), AgOTs (5.6 mg, 20 mol%), $Cu(OAc)_2 \cdot H_2O$ (39.9 mg, 0.2 mmol, 2.0 equiv.), $Cl_3CCH_2OH$ (0.5 mL) and $CH_3OH$ (1.5 mL) under an $N_2$ atmosphere. The resulting mixture was stirred at 80 °C for 24 h and then diluted with 10 mL of dichloromethane. The mixture was filtered through a celite pad and washed with 20 mL of dichloromethane. The filtrate was concentrated under reduced pressure, followed by dissolved in $CDCl_3$. In-situ $^1H$ NMR spectra of all the reaction mixtures showed that there was no product **6a** obtained.

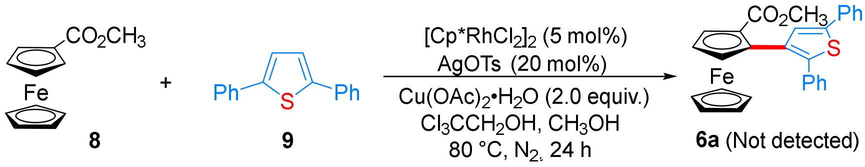


A 25 mL Schlenk sealed tube with a magnetic stir bar was charged with methyl ferrocene carboxylate **8** (48.8 mg, 0.2 mmol, 2.0 equiv.), 2,5-diphenylthiophene **9** (23.6 mg, 0.1 mmol), $[Cp^*RhCl_2]_2$ (3.1 mg, 5 mol%), AgOTs (5.6 mg, 20 mol%), $Cu(OAc)_2 \cdot H_2O$ (39.9 mg, 0.2 mmol, 2.0 equiv.), $Cl_3CCH_2OH$ (0.5 mL) and $CH_3OH$ (1.5 mL) under an $N_2$ atmosphere. The resulting mixture was stirred at 80 °C for 24 h and then diluted with 10 mL of dichloromethane. The mixture was filtered through a celite pad and washed with 20 mL of dichloromethane. The filtrate was concentrated under reduced pressure, followed by dissolved in $CDCl_3$. In-situ $^1H$ NMR spectra of all the reaction mixtures showed that there was no product **6a** obtained.

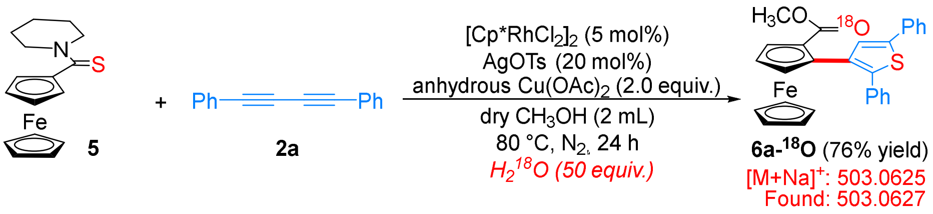


A 25 mL Schlenk sealed tube with a magnetic stir bar was charged with ferrocenyl thioamide **5** (62.6 mg, 0.2 mmol, 2.0 equiv.), 1,4-diphenylbuta-1,3-diyne **2a** (20.2 mg, 0.1 mmol), heavy-oxygen water ($H_2{}^{18}O$, 90 $\mu$L, 5.0 mmol, 50 equiv.), $[Cp^*RhCl_2]_2$ (3.1

mg, 5 mol%), AgOTs (5.6 mg, 20 mol%), anhydrous $Cu(OAc)_2$ (36.3 mg, 0.2 mmol, 2.0 equiv.) and dry $CH_3OH$ (2 mL) under an $N_2$ atmosphere. The resulting mixture was stirred at 80 °C for 24 h and then diluted with 10 mL of dichloromethane. The mixture was filtered through a celite pad and washed with 20 mL of dichloromethane. The filtrate was concentrated under reduced pressure and purified by column chromatography on silica gel (petroleum ether/ethyl acetate = 15:1, v/v), providing 36.4 mg of compound **6a-$^{18}$O** in 76% yield. The ESI-HRMS of **6a-$^{18}$O** calculated for $C_{28}H_{22}Fe^{16}O^{18}OSNa$ $[M+Na]^+$ 503.0625, was found 503.0627.

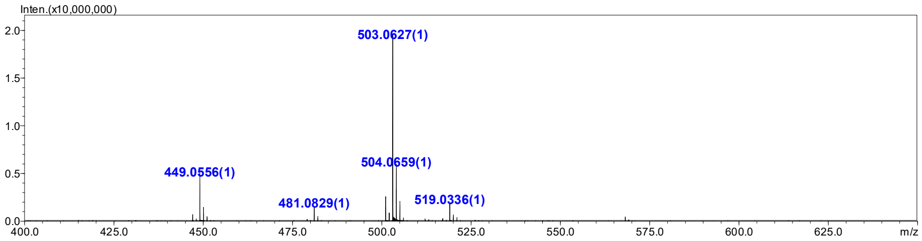


**Fig. S1.** ESI-HRMS spectrum of **6a-$^{18}$O**.

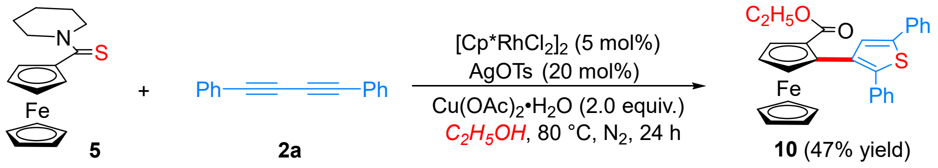


A 25 mL Schlenk sealed tube with a magnetic stir bar was charged with ferrocenyl thioamide **5** (62.6 mg, 0.2 mmol, 2.0 equiv.), 1,4-diphenylbuta-1,3-diyne **2a** (20.2 mg, 0.1 mmol), $[Cp^*RhCl_2]_2$ (3.1 mg, 5 mol%), AgOTs (5.6 mg, 20 mol%), $Cu(OAc)_2{\cdot}H_2O$ (39.9 mg, 0.2 mmol, 2.0 equiv.) and $CH_3CH_2OH$ (2 mL) under an $N_2$ atmosphere. The resulting mixture was stirred at 80 °C for 24 h and then diluted with 10 mL of dichloromethane. The mixture was filtered through a celite pad and washed with 20 mL of dichloromethane. The filtrate was concentrated under reduced pressure, and purified by column chromatography on silica gel (petroleum ether/ethyl acetate = 15:1, v/v) to provide compound **10** as a yellow solid in a yield of 47% (23.3 mg).

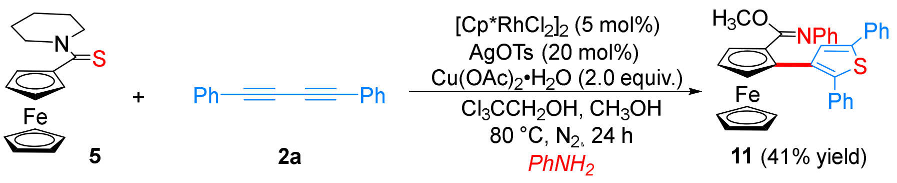

A 25 mL Schlenk sealed tube with a magnetic stir bar was charged with ferrocenyl thioamide **5** (62.6 mg, 0.2 mmol, 2.0 equiv.), 1,4-diphenylbuta-1,3-diyne **2a** (20.2 mg, 0.1 mmol), aniline (27 $\mu$L, 0.3 mmol, 3.0 equiv.), $[Cp^*RhCl_2]_2$ (3.1 mg, 5 mol%), AgOTs (5.6 mg, 20 mol%), $Cu(OAc)_2{\cdot}H_2O$ (39.9 mg, 0.2 mmol, 2.0 equiv.), $Cl_3CCH_2OH$ (0.5 mL) and $CH_3OH$ (1.5 mL) under an $N_2$ atmosphere. The resulting mixture was stirred at 80 °C for 24 h and then diluted with 10 mL of dichloromethane. The mixture was filtered through a celite pad and washed with 20 mL of dichloromethane. The filtrate was concentrated under reduced pressure and purified by column chromatography on silica gel (petroleum ether/ethyl acetate = 15:1, v/v) to provide compound **11** as a yellow solid in 41% yield (22.7 mg).

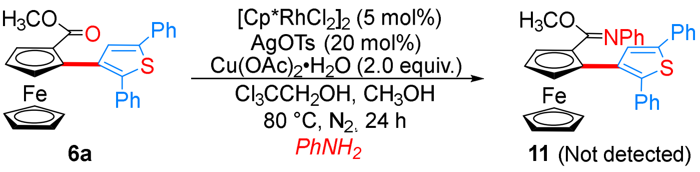


A 25 mL Schlenk sealed tube with a magnetic stir bar was charged with thienylferrocene **6a** (47.8 mg, 0.1 mmol), aniline (27 $\mu$L, 0.3 mmol, 3.0 equiv.), $[Cp^*RhCl_2]_2$ (3.1 mg, 5 mol%), AgOTs (5.6 mg, 20 mol%), $Cu(OAc)_2{\cdot}H_2O$ (39.9 mg, 0.2 mmol, 2.0 equiv.), $Cl_3CCH_2OH$ (0.5 mL) and $CH_3OH$ (1.5 mL) under an $N_2$ atmosphere. The resulting mixture was stirred at 80 °C for 24 h and then diluted with 10 mL of dichloromethane. The mixture was filtered through a celite pad and washed with 20 mL of dichloromethane. The filtrate was concentrated under reduced pressure, followed by dissolved in $CDCl_3$. In-situ $^1H$ NMR spectra of the reaction mixture showed that there was no product **11** obtained.

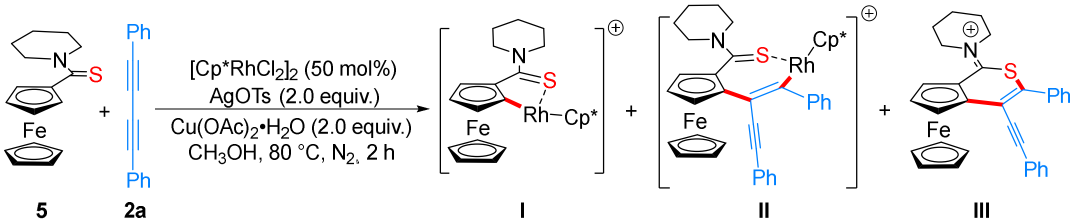


A 25 mL Schlenk sealed tube with a magnetic stir bar was charged with ferrocenyl thioamide **5** (31.3 mg, 0.1 mmol), 1,4-diphenylbuta-1,3-diyne **2a** (20.2 mg, 0.1 mmol),

[Cp*RhCl2]2 (30.9 mg, 50 mol%), AgOTs (55.8 mg, 2.0 equiv.), $Cu(OAc)_2 \cdot H_2O$ (39.9 mg, 0.2 mmol, 2.0 equiv.) and $CH_3OH$ (2 mL) under an $N_2$ atmosphere. The resulting mixture was stirred at 80 °C for 2 h, and then cooled to room temperature for ESI-HRMS detection.

Intermediate **I**: calcd for $C_{26}H_{33}FeNRhS$ $[M]^+$ 550.0733, found 550.0731;

Intermediate **II**: calcd for $C_{42}H_{43}FeNRhS$ $[M]^+$ 752.1515, found 752.1521;

Intermediate **III**: calcd for $C_{32}H_{28}FeNS$ $[M]^+$ 514.1286, found 514.1289.

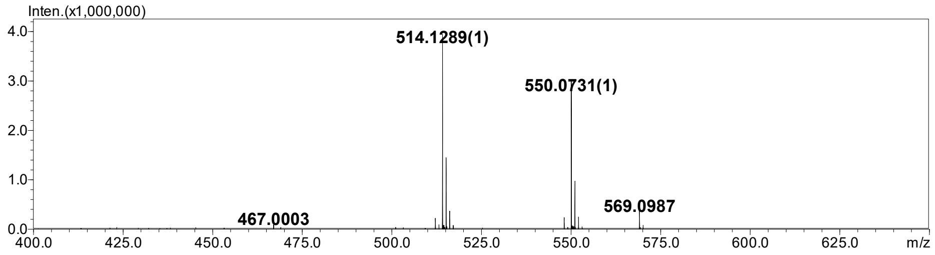


**Fig. S2.** ESI-HRMS spectrum of intermediates **I** and **III**.

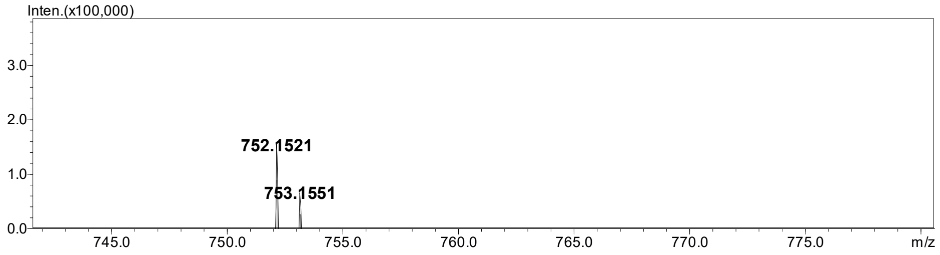


**Fig. S3.** ESI-HRMS spectrum of intermediate **II**.

## VIII. Synthesis of extended π-conjugated ferrocenes 7

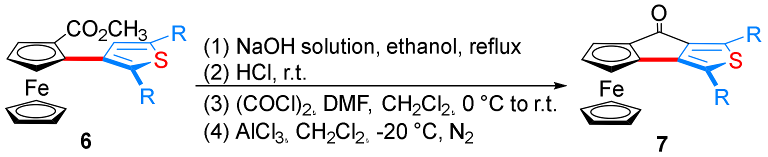


4 M of NaOH solution (15 mL) was added to the solution of thienylferrocene **6** (0.3 mmol) and EtOH (45 mL). Monitored by thin-layer chromatography, the mixture was refluxed until hydrolyzed completely. Then, 2 N of HCl solution was added carefully at room temperature until the mixture was weakly acidic. After evaporated under reduced pressure, the residue was diluted with 150 mL of ethyl acetate and washed

with saline solution (200 mL) three times. The organic layer was dried over $MgSO_4$, filtered, and concentrated under reduced pressure. The residue was re-dissolved in 50 mL of dichloromethane and filtered out the insoluble. Finally, the filtrate was concentrated under reduced pressure to give the hydrolysate.

Then, oxalyl chloride (45 $\mu$L, 0.53 mmol) was added to the solution of the hydrolysate in dry dichloromethane (20 mL) at 0 °C under $N_2$, followed by two drops of DMF. The mixture was stirred for 6 h at room temperature and evaporated under reduced pressure. To a mixture of $AlCl_3$ (160.0 mg, 1.2 mmol) in dry dichloromethane (20 mL) cooled to −20 °C, the solution of the prepared acyl chloride re-dissolved in dry dichloromethane (20 mL), which had also been cooled to −20 °C, was added *via* a double-ended needle under an $N_2$ atmosphere. The resulting mixture was stirred for overnight at −20 °C. Then 2 mL water was poured into the reaction flask at −20 °C, stirring for 5 min. The mixture was diluted with 100 mL of dichloromethane and washed with $NH_4Cl$, $NaHCO_3$ and saline solution (150 mL) successively. The organic layer was dried over $MgSO_4$, filtered, concentrated under reduced pressure, and purified by column chromatography on silica gel to provide extended π-conjugated ferrocenes **7**.

## IX. Calculated molecular conformations, photophysical and electrochemical properties

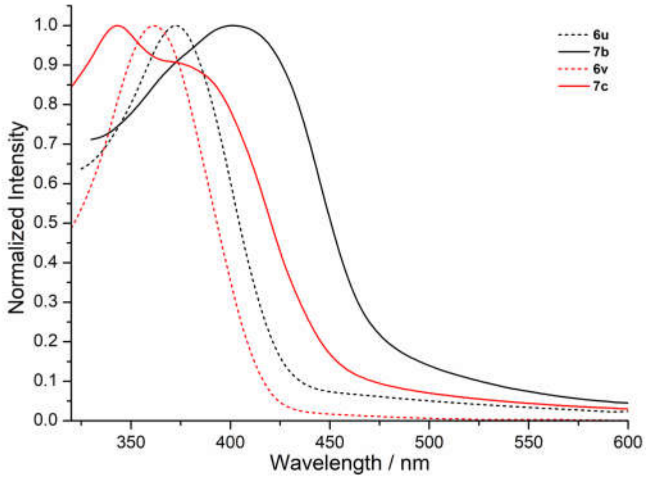


**Fig. S4** UV-Vis absorption spectra of **6u**, **6v**, **7b** and **7c** in $CH_3CN$ ($1 \times 10^{-5}$ M).

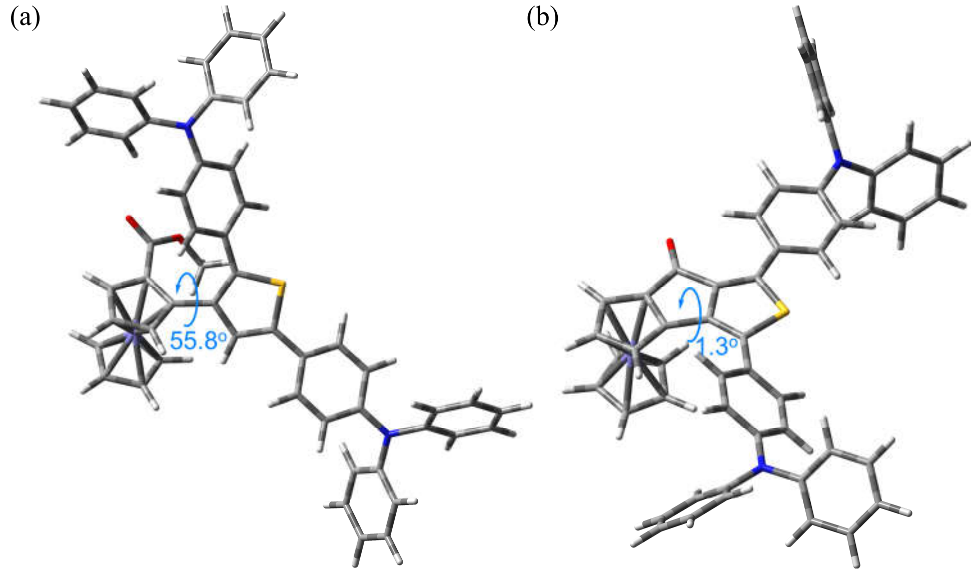


**Fig. S5** Calculated molecular conformation of (a) **6u** and (b) **7b**. Molecular optimization was performed by density functional theory computation with Gaussian 09 at the B3LYP/6-31G* level.

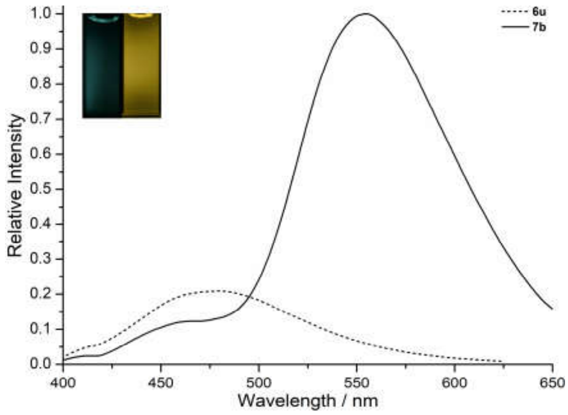


**Fig. S6** Fluorescence spectra of **6u** and **7b** in $CH_3CN$ ($1 \times 10^{-5}$ M). Inset: Fluorescent images of **6u** (left) and **7b** (right) in $CH_3CN$ ($1 \times 10^{-5}$ M) under UV light (365 nm).

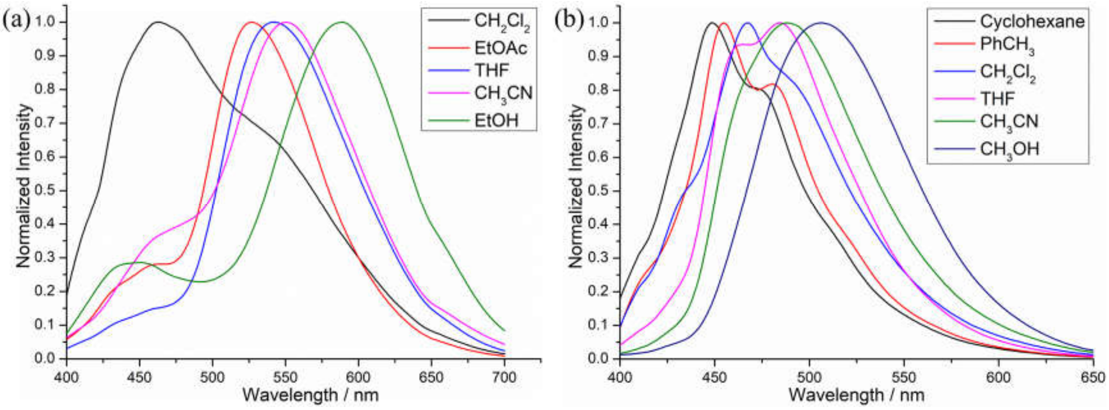


**Fig. S7** Fluorescence spectra of (a) **7b** and (b) **7c** in different solvents.

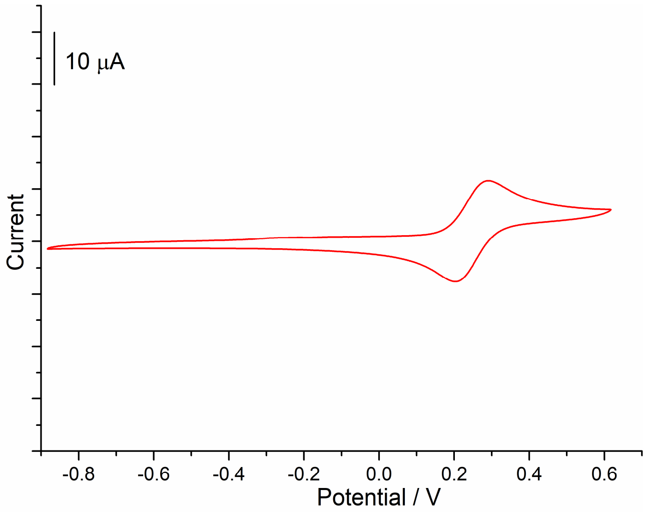


**Fig. S8** Cyclic voltammograms of **6v**. Measurements were carried out at a scan rate of 50 mV/s in $CH_3CN$ with **6v** ($1 \times 10^{-3}$ M) containing *n*-$Bu_4NPF_6$ (0.1 M), using $Fc/Fc^+$ (0.08 V) as reference. The three-electrode setup consisted of an $Ag/Ag^+$ (0.01 M of $AgNO_3$ in $CH_3CN$) reference electrode, a platinum wire counter electrode, and a platinum plate working electrode.

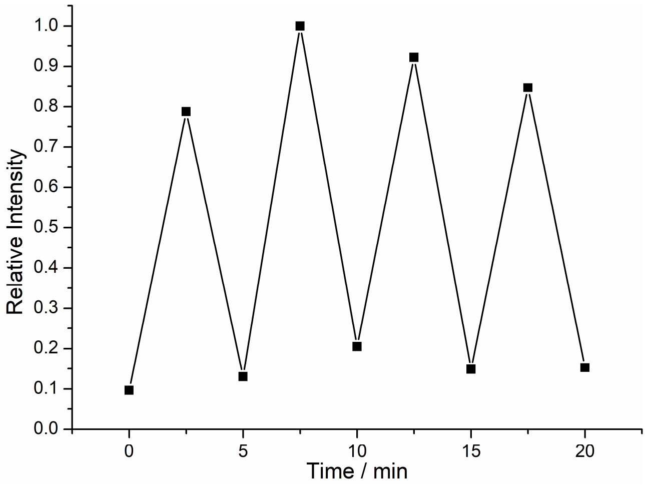


**Fig. S9** Changes by the evolution of the fluorescence intensity of **6v** at 432 nm with time. Stepwise oxidation and reduction cycles carried out in $CH_3CN$ with **6v** ($5 \times 10^{-6}$ M) containing *n*-$Bu_4NPF_6$ (0.1 M) by chronoamperometric analysis, which uses fixed potentials at + 0.48 V (for oxidation, vs $Fc/Fc^+$) and – 0.62 V (for reduction, vs $Fc/Fc^+$) changed every 150 s.

## X. Preparation and characterization of the described compounds

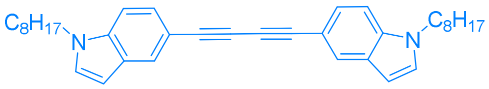


**1,4-Bis(1-octyl-1*H*-indol-5-yl)buta-1,3-diyne (2s)**

$^{1}H$ NMR (400 MHz, $CDCl_3$): $\delta$ = 0.87 (t, $J$ = 7.2 Hz, 6H), 1.25-1.30 (m, 20H), 1.79-1.86 (m, 4H), 4.09 (t, $J$ = 7.2 Hz, 4H), 6.48 (d, $J$ = 2.8 Hz, 2H), 7.12 (d, $J$ = 2.8 Hz, 2H), 7.28 (d, $J$ = 8.4 Hz, 2H), 7.36 (dd, $J$ = 8.4 Hz, 1.6 Hz, 2H), 7.85 (s, 2H) ppm. $^{13}C$ NMR (100 MHz, $CDCl_3$): $\delta$ = 14.2, 22.7, 27.1, 29.29, 29.32, 30.4, 31.9, 46.7, 72.3, 82.8, 101.5, 109.7, 112.5, 125.8, 126.2, 128.5, 129.1, 136.1 ppm.

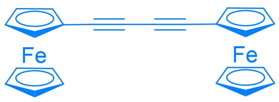


**1,4-Diferrocenylbuta-1,3-diyne** (**2t**)

$^{1}H$ NMR (400 MHz, $CDCl_3$): $\delta$ = 4.25 (t, $J$ = 2.0 Hz, 4H), 4.26 (s, 10H), 4.51 (t, $J$ = 2.0 Hz, 4H) ppm. $^{13}C$ NMR (100 MHz, $CDCl_3$): $\delta$ = 63.8, 69.4, 70.3, 71.1, 72.3, 79.2 ppm.

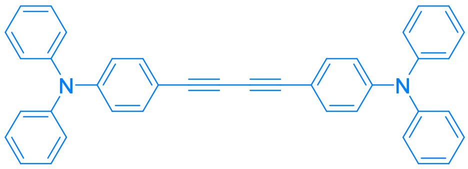


**4,4'-(buta-1,3-diyne-1,4-diyl)bis(*N*,*N*-diphenylaniline)** (**2u**)

$^{1}H$ NMR (400 MHz, $CDCl_3$): $\delta$ = 6.95 (dt, $J$ = 8.8 Hz, 2.0 Hz, 4H), 7.06-7.12 (m, 12H), 7.27-7.30 (m, 8H), 7.34 (dt, $J$ = 8.8 Hz, 2.0 Hz, 4H) ppm. $^{13}C$ NMR (100 MHz, $CDCl_3$): $\delta$ = 73.6, 82.1, 114.3, 121.7, 124.1, 125.5, 129.6, 133.5, 147.0, 148.7 ppm.

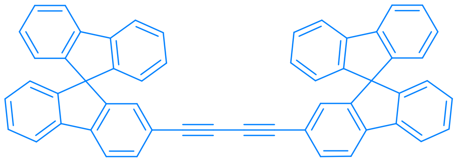

**1,4-Di(9,9'-spirobifluoren-2-yl)buta-1,3-diyne (2v)**

$^{1}H$ NMR (400 MHz, $CDCl_3$): $\delta$ = 6.70 (d, $J$ = 7.6 Hz, 4H), 6.74 (d, $J$ = 7.6 Hz, 2H), 6.82 (s, 2H), 7.08-7.15 (m, 6H), 7.34-7.39 (m, 6H), 7.46 (dd, $J$ = 8.0 Hz, 1.6 Hz, 2H), 7.76 (d, $J$ = 8.0 Hz, 2H), 7.81-7.84 (m, 6H) ppm. $^{13}C$ NMR (100 MHz, $CDCl_3$): $\delta$ = 65.8, 74.4, 82.4, 120.1, 120.2, 120.6, 120.9, 124.1, 124.3, 128.0, 128.1, 128.2, 128.7, 132.4, 140.9, 141.9, 142.8, 148.0, 149.1, 149.3 ppm.

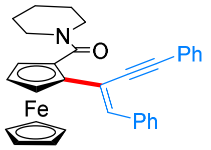


**N-[2-(1,4-diphenylbut-1-en-3-yn-2-yl)ferrocenylformyl]piperidine (4)**

M.p.: 84-87 °C. $^{1}H$ NMR (400 MHz, $CDCl_3$): $\delta$ = 1.45-1.71 (m, 6H), 3.11-3.22 (m, 2H), 3.45-3.48 (m, 1H), 3.64-3.67 (m, 1H), 4.33 (t, $J$ = 2.4 Hz, 1H), 4.40 (s, 5H), 4.57 (dd, $J$ = 2.4 Hz, 1.6 Hz, 1H), 4.60 (dd, $J$ = 2.4 Hz, 1.6 Hz, 1H), 6.93 (s, 1H), 7.31-7.44 (m, 6H), 7.67 (dt, $J$ = 6.4 Hz, 1.6 Hz, 2H), 8.00 (d, $J$ = 7.2 Hz, 2H) ppm. $^{13}C$ NMR (100 MHz, $CDCl_3$): $\delta$ = 24.6, 25.3, 26.2, 43.1, 47.9, 65.9, 67.5, 71.7, 72.8, 85.0, 85.6, 88.8, 96.6, 119.8, 123.6, 128.1, 128.5, 128.59, 128.64, 128.8, 131.8, 132.4, 136.9, 167.7 ppm. ESI-HRMS: calcd for $C_{32}H_{29}FeNONa$ $[M+Na]^+$ 522.1491, found 522.1487.

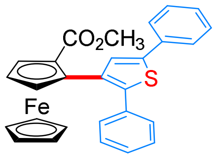


**Methyl 2-(2,5-diphenylthien-3-yl)ferrocene carboxylate (6a)**

Following the general procedure for regioselective addition/annulation reaction, compound **6a** was obtained as a yellow solid (38.8 mg, 81% yield) *via* column chromatography on silica gel (petroleum ether/ethyl acetate = 15/1, v/v). M.p.: 155-156 °C. $^{1}H$ NMR (400 MHz, $CDCl_3$): $\delta$ = 3.58 (s, 3H), 4.25 (s, 5H), 4.36 (dd, $J$ = 2.4 Hz, 1.6 Hz, 1H), 4.42 (t, $J$ = 2.4 Hz, 1H), 4.88 (dd, $J$ = 2.8 Hz, 1.6 Hz, 1H), 7.16-7.24 (m, 5H), 7.31-7.35 (m, 1H), 7.44 (t, $J$ = 8.0 Hz, 2H), 7.69-7.72 (m, 3H) ppm. $^{13}C$ NMR (100 MHz, $CDCl_3$): $\delta$ = 51.6, 70.0, 70.1, 70.8, 71.1, 74.6, 85.4, 125.9, 127.3, 127.6, 128.3, 128.7, 129.1, 129.2, 133.4, 134.5, 134.6, 140.1, 140.6, 171.7 ppm. ESI-HRMS: calcd for $C_{28}H_{23}FeO_2S$ $[M+H]^+$ 479.0763, found 479.0770.

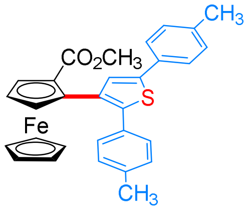

**Methyl 2-[2,5-di(4-methylphenyl)thien-3-yl]ferrocene carboxylate (6b)**

Following the general procedure for regioselective addition/annulation reaction, compound **6b** was obtained as a yellow solid (38.2 mg, 75% yield) *via* column chromatography on silica gel (petroleum ether/ethyl acetate = 15/1, v/v). M.p.: 93-95 °C. $^{1}$H NMR (400 MHz, $CDCl_3$): $\delta$ = 2.29 (s, 3H), 2.40 (s, 3H), 3.58 (s, 3H), 4.24 (s, 5H), 4.34 (dd, $J$ = 2.4 Hz, 1.6 Hz, 1H), 4.41 (t, $J$ = 2.4 Hz, 1H), 4.87 (dd, $J$ = 2.8 Hz, 1.6 Hz, 1H), 7.00-7.06 (m, 4H), 7.23 (d, $J$ = 8.0 Hz, 2H), 7.58 (d, $J$ = 8.4 Hz, 2H), 7.65 (s, 1H) ppm. $^{13}$C NMR (100 MHz, $CDCl_3$): $\delta$ = 21.3, 21.4, 51.6, 69.9, 70.1, 70.7, 71.1, 74.6, 85.7, 125.8, 128.5, 128.8, 129.0, 129.7, 131.8, 131.9, 132.8, 137.0, 137.4, 139.8, 140.3, 171.8 ppm. ESI-HRMS: calcd for $C_{30}H_{27}FeO_2S$ $[M+H]^+$ 507.1076, found 507.1082.

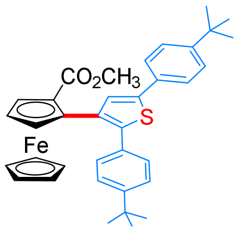


**Methyl 2-[2,5-di(4-*tert*-butylphenyl)thien-3-yl]ferrocene carboxylate (6c)**

Following the general procedure for regioselective addition/annulation reaction, compound **6c** was obtained as a yellow solid (40.9 mg, 69% yield) *via* column chromatography on silica gel (petroleum ether/ethyl acetate = 15/1, v/v). M.p.: 92-94 °C. $^{1}$H NMR (400 MHz, $CDCl_3$): $\delta$ = 1.28 (s, 9H), 1.37 (s, 9H), 3.55 (s, 3H), 4.23 (s, 5H), 4.38-4.39 (m, 1H), 4.42 (t, $J$ = 2.4 Hz, 1H), 4.87 (dd, $J$ = 2.4 Hz, 1.6 Hz, 1H), 7.10 (d, $J$ = 8.4 Hz, 2H), 7.22 (d, $J$ = 8.4 Hz, 2H), 7.45 (d, $J$ = 8.8 Hz, 2H), 7.62 (d, $J$ = 8.8 Hz, 2H), 7.65 (s, 1H) ppm. $^{13}$C NMR (100 MHz, $CDCl_3$): $\delta$ = 31.4, 31.5, 34.7, 34.8, 51.6, 69.9, 70.1, 70.7, 71.1, 74.7, 85.6, 125.2, 125.6, 125.9, 128.2, 129.0, 131.8, 131.9, 132.8, 139.7, 140.2, 150.1, 150.6, 171.7 ppm. ESI-HRMS: calcd for $C_{36}H_{38}FeO_2SNa$ $[M+Na]^+$ 613.1834, found 613.1835.

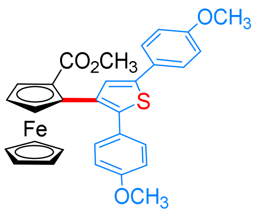


**Methyl 2-[2,5-di(4-methoxylphenyl)thien-3-yl]ferrocene carboxylate (6d)**

Following the general procedure for regioselective addition/annulation reaction, compound **6d** was obtained as a yellow solid (39.6 mg, 74% yield) *via* column chromatography on silica gel (petroleum ether/ethyl acetate = 10/1, v/v). M.p.: 164-167 °C. $^{1}$H NMR (400 MHz, $CDCl_3$): $\delta$ = 3.60 (s, 3H), 3.77 (s, 3H), 3.86 (s, 3H), 4.24 (s, 5H), 4.33 (dd, $J$ = 2.4 Hz, 1.6 Hz, 1H), 4.40 (t, $J$ = 2.4 Hz, 1H), 4.87 (dd, $J$ = 2.4 Hz, 1.6 Hz, 1H), 6.74 (dt, $J$ = 8.8 Hz, 2.0 Hz, 2H), 6.96 (dt, $J$ = 8.8 Hz, 2.0 Hz, 2H), 7.07 (dt, $J$ = 8.8 Hz, 2.0 Hz, 2H), 7.58 (s, 1H), 7.61 (dt, $J$ = 8.8 Hz, 2.0 Hz, 2H) ppm. $^{13}$C NMR (100 MHz, $CDCl_3$): $\delta$ = 51.6, 55.3, 55.5, 69.9, 70.0, 70.7, 71.1, 74.6, 85.7, 113.8, 114.4, 127.1, 127.3, 127.6, 128.3, 129.9, 132.5, 139.1, 139.8, 158.8, 159.2, 171.8 ppm. ESI-HRMS: calcd for $C_{30}H_{27}FeO_4S$ $[M+H]^+$ 539.0974, found 539.0977.

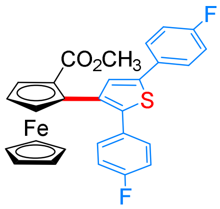


**Methyl 2-[2,5-di(4-fluorophenyl)thien-3-yl]ferrocene carboxylate (6e)**

Following the general procedure for regioselective addition/annulation reaction, compound **6e** was obtained as a yellow solid (37.2 mg, 72% yield) *via* column chromatography on silica gel (petroleum ether/ethyl acetate = 15/1, v/v). M.p.: 141-143 °C. $^{1}$H NMR (400 MHz, $CDCl_3$): $\delta$ = 3.61 (s, 3H), 4.24 (s, 5H), 4.30-4.31 (m, 1H), 4.42 (t, $J$ = 2.4 Hz, 1H), 4.87 (dd, $J$ = 2.4 Hz, 1.6 Hz, 1H), 6.88-6.94 (m, 2H), 7.08-7.16 (m, 4H), 7.62-7.67 (m, 3H) ppm. $^{13}$C NMR (100 MHz, $CDCl_3$): $\delta$ = 51.6, 69.9, 70.1, 70.9, 71.2, 74.5, 85.2, 115.4 (d, $J$ = 21.5 Hz), 116.1 (d, $J$ = 21.7 Hz), 127.6 (d, $J$ = 8.0 Hz), 129.2 (d, $J$ = 0.8 Hz), 130.4 (d, $J$ = 8.0 Hz), 130.6 (d, $J$ = 3.4 Hz), 130.7 (d, $J$ = 3.3 Hz), 133.5, 138.8 (d, $J$ = 0.8 Hz), 139.5, 162.1 (d, $J$ = 246.1 Hz), 162.5 (d, $J$ = 245.8 Hz),

171.5 ppm. $^{19}$F NMR (376 MHz, $CDCl_3$): $\delta$ = -114.54 – -114.48 (m, 1F), -114.47 – -114.41 (m, 1F) ppm. ESI-HRMS: calcd for $C_{28}H_{20}F_2FeO_2SNa$ $[M+Na]^+$ 537.0394, found 537.0396.

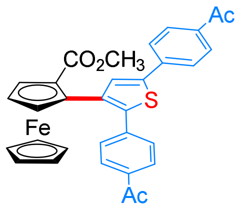


**Methyl 2-[2,5-di(4-acetylphenyl)thien-3-yl]ferrocene carboxylate (6f)**

Following the general procedure for regioselective addition/annulation reaction, compound **6f** was obtained as a yellow solid (38.5 mg, 68% yield) *via* column chromatography on silica gel (petroleum ether/ethyl acetate = 10/1, v/v). M.p.: 169-172 °C. $^1$H NMR (400 MHz, $CDCl_3$): $\delta$ = 2.56 (s, 3H), 2.65 (s, 3H), 3.59 (s, 3H), 4.27 (s, 5H), 4.35 (dd, $J$ = 2.4 Hz, 1.6 Hz, 1H), 4.47 (t, $J$ = 2.4 Hz, 1H), 4.92 (dd, $J$ = 2.4 Hz, 1.6 Hz, 1H), 7.24-7.26 (m, 2H), 7.77-7.82 (m, 4H), 7.86 (s, 1H), 8.04 (dt, $J$ = 8.4 Hz, 2.0 Hz, 2H) ppm. $^{13}$C NMR (100 MHz, $CDCl_3$): $\delta$ = 26.7, 26.8, 51.7, 70.0, 70.4, 71.2, 71.3, 74.6, 84.8, 125.7, 128.5, 128.6, 129.4, 131.1, 135.4, 135.7, 136.1, 138.6, 139.1, 140.2, 140.4, 171.4, 197.4, 197.6 ppm. ESI-HRMS: calcd for $C_{32}H_{26}FeO_4SNa$ $[M+Na]^+$ 585.0793, found 585.0797.

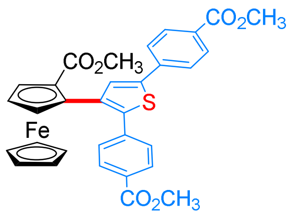


**Methyl 2-[2,5-di(4-methoxycarbonylphenyl)thien-3-yl]ferrocene carboxylate (6g)**

Following the general procedure for regioselective addition/annulation reaction, compound **6g** was obtained as a yellow solid (39.0 mg, 66% yield) *via* column chromatography on silica gel (petroleum ether/ethyl acetate = 2/1, v/v). M.p.: 189-191 °C. $^1$H NMR (400 MHz, $CDCl_3$): $\delta$ = 3.58 (s, 3H), 3.89 (s, 3H), 3.95 (s, 3H), 4.26 (s, 5H), 4.33 (dd, $J$ = 2.8 Hz, 1.6 Hz, 1H), 4.45 (t, $J$ = 2.8 Hz, 1H), 4.91 (dd, $J$ = 2.8 Hz,

1.6 Hz, 1H), 7.22 (dt, $J$ = 8.4 Hz, 2.0 Hz, 2H), 7.75 (dt, $J$ = 8.4 Hz, 2.0 Hz, 2H), 7.84 (s, 1H), 7.88 (dt, $J$ = 8.4 Hz, 2.0 Hz, 2H), 8.10 (dt, $J$ = 8.4 Hz, 2.0 Hz, 2H) ppm. $^{13}C$ NMR (100 MHz, $CDCl_3$): $\delta$ = 51.7, 52.26, 52.34, 70.0, 70.3, 71.17, 71.24, 74.6, 84.8, 125.5, 128.4, 128.8, 129.2, 129.7, 130.5, 130.9, 135.2, 138.5, 139.0, 140.1, 140.4, 166.9, 171.4 ppm. ESI-HRMS: calcd for $C_{32}H_{26}FeO_6SNa$ $[M+Na]^+$ 617.0692, found 617.0694.

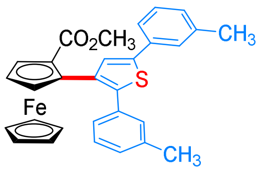


**Methyl 2-[2,5-di(3-methylphenyl)thien-3-yl]ferrocene carboxylate (6h)**

Following the general procedure for regioselective addition/annulation reaction, compound **6h** was obtained as a yellow solid (36.2 mg, 71% yield) *via* column chromatography on silica gel (petroleum ether/ethyl acetate = 15/1, v/v). M.p.: 59-63 °C. $^1H$ NMR (400 MHz, $CDCl_3$): $\delta$ = 2.25 (s, 3H), 2.44 (s, 3H), 3.58 (s, 3H), 4.24 (s, 5H), 4.35 (dd, $J$ = 2.4 Hz, 1.6 Hz, 1H), 4.41 (t, $J$ = 2.4 Hz, 1H), 4.87 (dd, $J$ = 2.4 Hz, 1.6 Hz, 1H), 6.92 (d, $J$ = 7.6 Hz, 1H), 6.99-7.02 (m, 2H), 7.08 (t, $J$ = 7.6 Hz, 1H), 7.14 (d, $J$ = 7.6 Hz, 1H), 7.32 (t, $J$ = 7.6 Hz, 1H), 7.50-7.52 (m, 2H), 7.68 (s, 1H) ppm. $^{13}C$ NMR (100 MHz, $CDCl_3$): $\delta$ = 21.5, 21.7, 51.6, 69.9, 70.1, 70.7, 71.1, 74.6, 85.5, 123.0, 125.8, 126.6, 128.0, 128.2, 128.4, 128.9, 129.1, 129.4, 133.2, 134.48, 134.51, 137.9, 138.7, 140.1, 140.6, 171.8 ppm. ESI-HRMS: calcd for $C_{30}H_{27}FeO_2S$ $[M+H]^+$ 507.1076, found 507.1076.

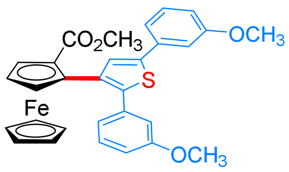


**Methyl 2-[2,5-di(3-methoxylphenyl)thien-3-yl]ferrocene carboxylate (6i)**

Following the general procedure for regioselective addition/annulation reaction, compound **6i** was obtained as a yellow solid (39.5 mg, 73% yield) *via* column chromatography on silica gel (petroleum ether/ethyl acetate = 10/1, v/v). $^1H$ NMR

(400 MHz, DMSO-$d_6$): $\delta$ = 3.43 (s, 3H), 3.59 (s, 3H), 3.85 (s, 3H), 4.30 (s, 5H), 4.49 (br, 1H), 4.59 (br, 1H), 4.81 (br, 1H), 6.55 (s, 1H), 6.72 (d, $J$ = 7.6 Hz, 1H), 6.78 (d, $J$ = 8.4 Hz, 1H), 6.96 (d, $J$ = 8.0 Hz, 1H), 7.17 (t, $J$ = 8.0 Hz, 1H), 7.25 (s, 1H), 7.34 (d, $J$ = 7.6 Hz, 1H), 7.41 (t, $J$ = 8.0 Hz, 1H), 7.79 (s, 1H) ppm. $^{13}$C NMR (100 MHz, DMSO-$d_6$): $\delta$ = 51.2, 54.9, 55.3, 69.7, 70.1, 70.2, 71.0, 74.1, 84.7, 111.0, 112.9, 113.2, 113.3, 117.9, 120.2, 129.6, 129.7, 130.4, 133.9, 134.8, 135.1, 138.4, 139.4, 158.9, 159.8, 170.4 ppm. ESI-HRMS: calcd for $C_{30}H_{27}FeO_4S$ $[M+H]^+$ 539.0974, found 539.0968.

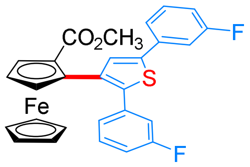


**Methyl 2-[2,5-di(3-fluorophenyl)thien-3-yl]ferrocene carboxylate (6j)**

Following the general procedure for regioselective addition/annulation reaction, compound **6j** was obtained as a yellow solid (33.3 mg, 65% yield) *via* column chromatography on silica gel (petroleum ether/ethyl acetate = 15/1, v/v). $^1$H NMR (400 MHz, $CDCl_3$): $\delta$ = 3.61 (s, 3H), 4.26 (s, 5H), 4.35 (dd, $J$ = 2.8 Hz, 1.6 Hz, 1H), 4.45 (t, $J$ = 2.8 Hz, 1H), 4.91 (dd, $J$ = 2.8 Hz, 1.6 Hz, 1H), 6.84-6.95 (m, 3H), 7.00-7.05 (m, 1H), 7.15-7.21 (m, 1H), 7.37-7.43 (m, 2H), 7.47 (dt, $J$ = 8.0 Hz, 1.2 Hz, 1H), 7.72 (s, 1H) ppm. $^{13}$C NMR (100 MHz, $CDCl_3$): $\delta$ = 51.6, 69.9, 70.2, 71.1, 71.2, 74.5, 84.8, 112.6 (d, $J$ = 22.6 Hz), 114.3 (d, $J$ = 21.1 Hz), 114.5 (d, $J$ = 21.1 Hz), 115.4 (d, $J$ = 22.4 Hz), 121.6 (d, $J$ = 2.8 Hz), 124.4 (d, $J$ = 2.9 Hz), 129.9 (d, $J$ = 8.5 Hz), 130.0, 130.6 (d, $J$ = 8.6 Hz), 134.3, 136.39 (d, $J$ = 8.1 Hz), 136.43 (d, $J$ = 8.3 Hz), 139.0 (d, $J$ = 2.5 Hz), 139.7 (d, $J$ = 2.7 Hz), 162.5 (d, $J$ = 244.5 Hz), 163.3 (d, $J$ = 244.6 Hz), 171.5 ppm. $^{19}$F NMR (376 MHz, $CDCl_3$): $\delta$ = -112.95 – -112.88 (m, 1F), -112.68 – -112.62 (m, 1F) ppm. ESI-HRMS: calcd for $C_{28}H_{21}F_2FeO_2S$ $[M+H]^+$ 515.0574, found 515.0571.

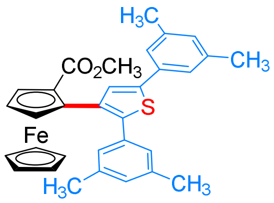


**Methyl 2-[2,5-di(3,5-dimethylphenyl)thien-3-yl]ferrocene carboxylate (6k)**

Following the general procedure for regioselective addition/annulation reaction, compound **6k** was obtained as a yellow solid (38.5 mg, 72% yield) *via* column chromatography on silica gel (petroleum ether/ethyl acetate = 15/1, v/v). M.p.: 88-91 °C. $^1$H NMR (400 MHz, $CDCl_3$): $\delta$ = 2.19 (s, 6H), 2.40 (s, 6H), 3.58 (s, 3H), 4.24 (s, 5H), 4.34 (dd, $J$ = 2.4 Hz, 1.6 Hz, 1H), 4.40 (t, $J$ = 2.4 Hz, 1H), 4.87 (dd, $J$ = 2.8 Hz, 1.6 Hz, 1H), 6.77 (s, 2H), 6.82 (s, 1H), 6.96 (s, 1H), 7.30 (s, 2H), 7.64 (s, 1H) ppm. $^{13}$C NMR (100 MHz, $CDCl_3$): $\delta$ = 21.4, 21.6, 51.6, 69.9, 70.0, 70.7, 71.1, 74.7, 85.6, 123.8, 126.5, 128.8, 128.9, 129.3, 133.0, 134.4, 134.5, 137.7, 138.6, 140.1, 140.6, 171.8 ppm. ESI-HRMS: calcd for $C_{32}H_{31}FeO_2S$ $[M+H]^+$ 535.1389, found 535.1395.

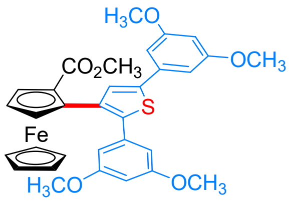


**Methyl 2-[2,5-di(3,5-dimethoxylphenyl)thien-3-yl]ferrocene carboxylate (6l)**

Following the general procedure for regioselective addition/annulation reaction, compound **6l** was obtained as a yellow solid (39.9 mg, 67% yield) *via* column chromatography on silica gel (petroleum ether/ethyl acetate = 7/1, v/v). M.p.: 125-129 °C. $^1$H NMR (400 MHz, $CDCl_3$): $\delta$ = 3.59 (s, 3H), 3.64 (s, 6H), 3.88 (s, 6H), 4.25 (s, 5H), 4.38-4.39 (m, 1H), 4.43 (t, $J$ = 2.8 Hz, 1H), 4.88 (dd, $J$ = 2.8 Hz, 1.6 Hz, 1H), 6.29-6.32 (m, 3H), 6.45 (t, $J$ = 2.0 Hz, 1H), 6.84 (d, $J$ = 2.0 Hz, 2H), 7.66 (s, 1H) ppm. $^{13}$C NMR (100 MHz, $CDCl_3$): $\delta$ = 51.6, 55.4, 55.6, 70.0, 70.2, 70.7, 71.1, 74.7, 85.4, 99.3, 100.1, 104.4, 106.5, 129.6, 133.5, 136.2, 136.4, 139.9, 140.4, 160.4, 161.2, 171.6 ppm. ESI-HRMS: calcd for $C_{32}H_{31}FeO_6S$ $[M+H]^+$ 599.1185, found 599.1181.

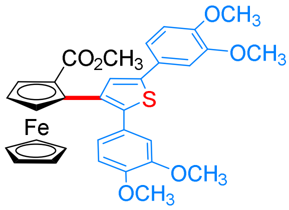


**Methyl 2-[2,5-di(3,4-dimethoxylphenyl)thien-3-yl]ferrocene carboxylate (6m)**

Following the general procedure for regioselective addition/annulation reaction, compound **6m** was obtained as a yellow solid (41.5 mg, 69% yield) *via* column chromatography on silica gel (petroleum ether/ethyl acetate = 6/1, v/v). M.p.: 153-156 °C. $^{1}$H NMR (400 MHz, DMSO-$d_6$): $\delta$ = 3.44 (s, 3H), 3.49 (s, 3H), 3.71 (s, 3H), 3.81 (s, 3H), 3.88 (s, 3H), 4.29 (s, 5H), 4.48 (br, 1H), 4.58 (t, $J$ = 2.4 Hz, 1H), 4.80 (br, 1H), 6.50 (s, 1H), 6.71 (dd, $J$ = 8.4 Hz, 2.0 Hz, 1H), 6.85 (d, $J$ = 8.4 Hz, 1H), 7.05 (d, $J$ = 8.4 Hz, 1H), 7.22 (s, 1H), 7.27 (dd, $J$ = 8.4 Hz, 2.0 Hz, 1H), 7.64 (s, 1H) ppm. $^{13}$C NMR (100 MHz, DMSO-$d_6$): $\delta$ = 51.1, 55.0, 55.4, 55.62, 55.65, 69.4, 69.8, 69.9, 70.9, 74.1, 85.1, 109.2, 111.3, 111.6, 112.3, 117.9, 120.2, 126.5, 126.6, 128.4, 132.7, 137.8, 139.0, 147.9, 148.0, 148.7, 149.1, 170.4 ppm. ESI-HRMS: calcd for $C_{32}H_{31}FeO_6S$ $[M+H]^+$ 599.1185, found 599.1185.

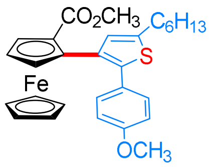


**Methyl 2-[5-hexyl-2-(4-methoxylphenyl)thien-3-yl]ferrocene carboxylate (6n)**

Following the general procedure for regioselective addition/annulation reaction, $CH_3OH$ (2.0 mL) was used as solvent, and compound **6n** was obtained as a yellow liquid (13.8 mg, 27% yield) *via* column chromatography on silica gel (petroleum ether/ethyl acetate = 15/1, v/v). $^{1}$H NMR (400 MHz, $CDCl_3$): $\delta$ = 0.92 (t, $J$ = 7.2 Hz, 3H), 1.33-1.38 (m, 4H), 1.44-1.50 (m, 2H), 1.73-1.80 (m, 2H), 2.86 (t, $J$ = 7.6 Hz, 2H), 3.60 (s, 3H), 3.75 (s, 3H), 4.20 (s, 5H), 4.25 (dd, $J$ = 2.8 Hz, 1.6 Hz, 1H), 4.35 (t, $J$ = 2.8 Hz, 1H), 4.84 (dd, $J$ = 2.8 Hz, 1.6 Hz, 1H), 6.71 (dt, $J$ = 9.2 Hz, 2.8 Hz, 2H), 7.02 (dt, $J$ = 8.8 Hz, 2.8 Hz, 2H), 7.15 (t, $J$ = 0.8 Hz, 1H) ppm. $^{13}$C NMR (100 MHz, $CDCl_3$): $\delta$ = 14.3, 22.8, 29.1, 30.3, 31.81, 31.83, 51.5, 55.3, 69.8, 69.9, 70.6, 71.0, 74.7, 85.9, 113.6, 127.7, 129.9, 130.2, 131.0, 137.9, 141.6, 158.5, 171.9 ppm. ESI-HRMS: calcd for $C_{29}H_{32}FeO_3SNa$ $[M+Na]^+$ 539.1314, found 539.1311.

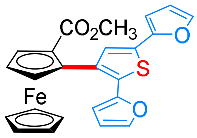

**Methyl 2-[2,5-di(furan-2-yl)thien-3-yl]ferrocene carboxylate (6o)**

Following the general procedure for regioselective addition/annulation reaction, $CH_3OH$ (2.0 mL) was used as solvent, and compound **6o** was obtained as a yellow solid (20.7 mg, 45% yield) *via* column chromatography on silica gel (petroleum ether/ethyl acetate = 20/1, v/v). $^1$H NMR (400 MHz, $CDCl_3$): $\delta$ = 3.60 (s, 3H), 4.30 (s, 5H), 4.51-4.54 (m, 2H), 4.96 (dd, $J$ = 2.4 Hz, 2.0 Hz, 1H), 5.81 (dd, $J$ = 3.6 Hz, 0.8 Hz, 1H), 6.26 (dd, $J$ = 3.6 Hz, 2.0 Hz, 1H), 6.49 (dd, $J$ = 3.6 Hz, 2.0 Hz, 1H), 6.56 (dd, $J$ = 3.6 Hz, 0.8 Hz, 1H), 7.28 (dd, $J$ = 2.0 Hz, 0.8 Hz, 1H), 7.46 (dd, $J$ = 2.0 Hz, 0.8 Hz, 1H), 7.54 (s, 1H) ppm. $^{13}$C NMR (100 MHz, $CDCl_3$): $\delta$ = 51.6, 70.1, 70.4, 70.7, 71.2, 74.5, 85.1, 105.5, 107.0, 111.7, 111.9, 128.4, 128.9, 130.0, 132.8, 141.3, 141.9, 149.0, 149.3, 171.7 ppm. ESI-HRMS: calcd for $C_{24}H_{19}FeO_4S$ $[M+H]^+$ 459.0348, found 459.0353.

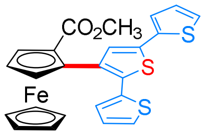


**Methyl 2-[2,5-di(thien-2-yl)thien-3-yl]ferrocene carboxylate (6p)**

Following the general procedure for regioselective addition/annulation reaction, $CH_3OH$ (2.0 mL) was used as solvent, and compound **6p** was obtained as a yellow solid (25.8 mg, 53% yield) *via* column chromatography on silica gel (petroleum ether/ethyl acetate = 20/1, v/v). M.p.: 160-162 °C. $^1$H NMR (400 MHz, $CDCl_3$): $\delta$ = 3.62 (s, 3H), 4.28 (s, 5H), 4.47-4.48 (m, 1H), 4.50 (t, $J$ = 2.8 Hz, 1H), 4.94 (dd, $J$ = 2.8 Hz, 1.6 Hz, 1H), 6.82 (dd, $J$ = 3.6 Hz, 1.2 Hz, 1H), 6.87 (dd, $J$ = 5.2 Hz, 3.6 Hz, 1H), 7.06 (dd, $J$ = 5.2 Hz, 3.6 Hz, 1H), 7.11 (dd, $J$ = 4.8 Hz, 1.2 Hz, 1H), 7.23 (dd, $J$ = 3.6 Hz, 1.2 Hz, 1H), 7.25-7.26 (m, 1H), 7.50 (s, 1H) ppm. $^{13}$C NMR (100 MHz, $CDCl_3$): $\delta$ = 51.7, 70.2, 70.8, 70.9, 71.1, 74.9, 84.7, 123.9, 124.6, 125.3, 125.8, 127.2, 128.1, 129.9, 132.7, 133.4, 133.6, 136.1, 137.3, 171.6 ppm. ESI-HRMS: calcd for $C_{24}H_{19}FeO_2S_3$ $[M+H]^+$ 490.9891, found 490.9896.

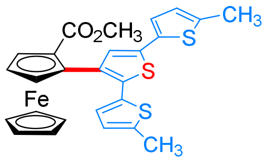


**Methyl 2-[2,5-di(5-methylthien-2-yl)thien-3-yl]ferrocene carboxylate (6q)**

Following the general procedure for regioselective addition/annulation reaction, $CH_3OH$ (2.0 mL) was used as solvent, and compound **6q** was obtained as a yellow solid (35.4 mg, 68% yield) *via* column chromatography on silica gel (petroleum ether/ethyl acetate = 20/1, v/v). M.p.: 153-157 °C. $^1H$ NMR (400 MHz, $CDCl_3$): $\delta$ = 2.36 (s, 3H), 2.51 (s, 3H), 3.64 (s, 3H), 4.27 (s, 5H), 4.47-4.49 (m, 2H), 4.93 (t, $J$ = 2.0 Hz, 1H), 6.50 (dd, $J$ = 3.6 Hz, 0.8 Hz, 1H), 6.58 (d, $J$ = 3.6 Hz, 1H), 6.70 (dd, $J$ = 3.6 Hz, 0.8 Hz, 1H), 6.99 (d, $J$ = 3.2 Hz, 1H), 7.39 (s, 1H) ppm. $^{13}C$ NMR (100 MHz, $CDCl_3$): $\delta$ = 15.4, 15.6, 51.7, 70.1, 70.7, 70.8, 71.1, 75.0, 84.9, 123.6, 125.4, 125.6, 126.1, 129.3, 132.6, 132.7, 133.2, 133.9, 135.0, 139.2, 139.9, 171.8 ppm. ESI-HRMS: calcd for $C_{26}H_{23}FeO_2S_3$ $[M+H]^+$ 519.0204, found 519.0205.

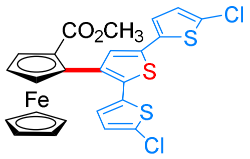


**Methyl 2-[2,5-di(5-chlorothien-2-yl)thien-3-yl]ferrocene carboxylate (6r)**

Following the general procedure for regioselective addition/annulation reaction, $CH_3OH$ (2.0 mL) was used as solvent, and compound **6r** was obtained as a yellow solid (26.3 mg, 47% yield) *via* column chromatography on silica gel (petroleum ether/ethyl acetate = 20/1, v/v). M.p.: 152-155 °C. $^1H$ NMR (400 MHz, $CDCl_3$): $\delta$ = 3.67 (s, 3H), 4.28 (s, 5H), 4.46 (br, 1H), 4.52 (br, 1H), 4.96 (br, 1H), 6.62 (d, $J$ = 4.0 Hz, 1H), 6.68 (d, $J$ = 4.0 Hz, 1H), 6.87 (d, $J$ = 4.0 Hz, 1H), 6.98 (d, $J$ = 4.0 Hz, 1H), 7.40 (s, 1H) ppm. $^{13}C$ NMR (100 MHz, $CDCl_3$): $\delta$ = 51.8, 70.5, 70.6, 71.16, 71.22, 75.0, 84.0, 123.2, 125.0, 126.2, 127.2, 129.0, 129.8, 130.1, 132.2, 132.6, 134.0, 134.6, 135.6, 171.4 ppm. ESI-HRMS: calcd for $C_{24}H_{17}{}^{35}Cl_2FeO_2S_3$ $[M+H]^+$ 558.9112, found 558.9108; $C_{24}H_{17}{}^{35}Cl^{37}ClFeO_2S_3$ $[M+H]^+$ 560.9082, found 560.9085; $C_{24}H_{17}{}^{37}Cl_2FeO_2S_3$ $[M+H]^+$ 562.9053, found 562.9049.

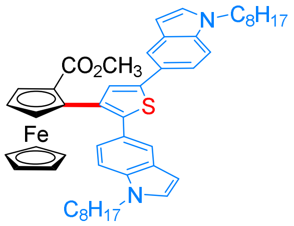


**Methyl 2-[2,5-di(1-octyl-1*H*-indol-5-yl)thien-3-yl]ferrocene carboxylate (6s)**

Following the general procedure for regioselective addition/annulation reaction, 1,2-dichloroethane (1.0 mL) and $CH_3OH$ (1.0 mL) were used as solvent, and compound **6s** was obtained as a yellow liquid (47.8 mg, 61% yield) *via* column chromatography on silica gel (petroleum ether/ethyl acetate = 15/1, v/v). $^1$H NMR (400 MHz, $CDCl_3$): $\delta$ = 0.86-0.90 (m, 6H), 1.29-1.34 (m, 20H), 1.80 (t, *J* = 6.8 Hz, 2H), 1.87 (t, *J* = 6.8 Hz, 2H), 3.60 (s, 3H), 4.05 (t, *J* = 7.2 Hz, 2H), 4.14 (t, *J* = 7.2 Hz, 2H), 4.25 (s, 5H), 4.34-4.35 (m, 2H), 4.88 (dd, *J* = 2.4 Hz, 1.6 Hz, 1H), 6.40 (d, *J* = 3.2 Hz, 1H), 6.55 (d, *J* = 2.8 Hz, 1H), 6.97 (dd, *J* = 8.4 Hz, 1.6 Hz, 1H), 7.05 (d, *J* = 3.2 Hz, 1H), 7.13-7.15 (m, 2H), 7.39 (d, *J* = 8.4 Hz, 1H), 7.52 (s, 1H), 7.58 (dd, *J* = 8.4 Hz, 1.6 Hz, 1H), 7.69 (s, 1H), 7.96 (s, 1H) ppm. $^{13}$C NMR (100 MHz, $CDCl_3$): $\delta$ = 14.2, 22.8, 27.2, 29.3, 29.4, 29.9, 30.4, 30.5, 31.91, 31.93, 46.6, 46.7, 51.6, 69.7, 70.2, 70.6, 71.0, 74.9, 86.1, 101.37, 101.40, 109.1, 109.9, 118.3, 120.4, 121.3, 123.0, 126.1, 126.6, 128.2, 128.3, 128.6, 128.8, 129.1, 131.8, 135.3, 135.7, 140.8, 141.4, 172.1 ppm. ESI-HRMS: calcd for $C_{48}H_{57}FeN_2O_2S$ $[M+H]^+$ 781.3485, found 781.3481.

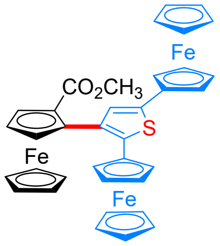


**Methyl 2-(2,5-diferrocenylthien-3-yl)ferrocene carboxylate (6t)**

Following the general procedure for regioselective addition/annulation reaction, 1,2-dichloroethane (1.0 mL) and $CH_3OH$ (1.0 mL) were used as solvent, and compound **6t** was obtained as a red solid (36.3 mg, 52% yield) *via* column chromatography on silica gel (petroleum ether/ethyl acetate = 15/1, v/v). M.p.:

113-117 °C. $^{1}H$ NMR (400 MHz, $CDCl_3$): $\delta$ = 3.62 (s, 3H), 4.04-4.05 (m, 1H), 4.06 (s, 5H), 4.08-4.09 (m, 1H), 4.11-4.12 (m, 1H), 4.14-4.15 (m, 1H), 4.21 (s, 5H), 4.24 (s, 5H), 4.32 (t, $J$ = 1.6 Hz, 2H), 4.40 (dd, $J$ = 2.4 Hz, 1.6 Hz, 1H), 4.44 (t, $J$ = 2.4 Hz, 1H), 4.63-4.65 (m, 2H), 4.89 (dd, $J$ = 2.4 Hz, 1.6 Hz, 1H), 7.19 (s, 1H) ppm. $^{13}C$ NMR (100 MHz, $CDCl_3$): $\delta$ = 51.5, 66.9, 67.0, 68.1, 68.2, 68.3, 68.7, 69.7, 70.0, 70.1, 70.5, 70.6, 70.9, 74.6, 80.2, 80.7, 86.2, 128.0, 132.1, 137.5, 137.7, 171.7 ppm. ESI-HRMS: calcd for $C_{36}H_{31}Fe_3O_2S$ $[M+H]^+$ 695.0088, found 695.0091.

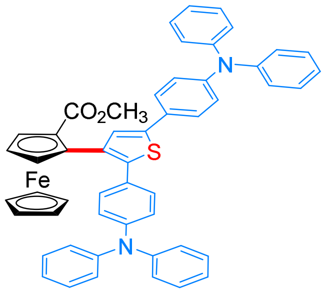


**Methyl 2-[2,5-di(4-diphenylaminophenyl)thien-3-yl]ferrocene carboxylate (6u)**

Following the general procedure for regioselective addition/annulation reaction, 1,2-dichloroethane (2.0 mL) and $CH_3OH$ (2.0 mL) were used as solvent, and the reaction mixture was stirred at 120 °C for 24 h under an $N_2$ atmosphere. Then, compound **6u** was obtained as a yellow solid (23.8 mg, 29% yield) *via* column chromatography on silica gel (petroleum ether/ethyl acetate/dichloromethane = 20/1/2, v/v/v). M.p.: 122-124 °C. $^{1}H$ NMR (400 MHz, $CDCl_3$): $\delta$ = 3.60 (s, 3H), 4.24 (s, 5H), 4.42 (br, 1H), 4.43 (br, 1H), 4.85 (t, $J$ = 2.0 Hz, 1H), 6.89 (d, $J$ = 8.4 Hz, 2H), 6.98-7.03 (m, 5H), 7.05-7.07 (m, 6H), 7.11-7.16 (m, 6H), 7.22-7.30 (m, 7H), 7.54 (d, $J$ = 8.8 Hz, 2H), 7.58 (s, 1H) ppm. $^{13}C$ NMR (100 MHz, $CDCl_3$): $\delta$ = 51.6, 69.9, 70.2, 70.7, 71.1, 74.5, 85.9, 123.1, 123.2, 124.0, 124.58, 124.60, 126.6, 128.4, 128.7, 128.8, 129.3, 129.4, 129.5, 132.8, 139.2, 139.8, 146.8, 147.3, 147.6, 147.7, 171.6 ppm. ESI-HRMS: calcd for $C_{52}H_{41}FeN_2O_2S$ $[M+H]^+$ 813.2233, found 813.2230.

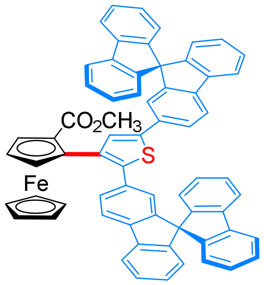


**Methyl 2-[2,5-di(9,9'-spirobifluoren-2-yl)thien-3-yl]ferrocene carboxylate (6v)**

Following the general procedure for regioselective addition/annulation reaction, 1,2-dichloroethane (2.0 mL) and $CH_3OH$ (2.0 mL) were used as solvent, and the reaction mixture was stirred at 120 °C for 24 h under an $N_2$ atmosphere. Then, compound **6v** was obtained as a yellow solid (34.2 mg, 36% yield) *via* column chromatography on silica gel (petroleum ether/ethyl acetate/dichloromethane = 10/1/2, v/v/v). M.p.: 204-206 °C. $^1$H NMR (400 MHz, $CDCl_3$): $\delta$ = 3.35 (s, 3H), 4.07-4.11 (m, 7H), 4.49 (dd, $J$ = 2.8 Hz, 1.6 Hz, 1H), 6.24 (d, $J$ = 2.0 Hz, 1H), 6.61-6.67 (m, 3H), 6.71 (d, $J$ = 7.6 Hz, 1H), 6.77 (d, $J$ = 7.6 Hz, 2H), 6.88 (d, $J$ = 1.6 Hz, 1H), 7.01-7.14 (m, 7H), 7.28-7.40 (m, 7H), 7.62 (d, $J$ = 8.0 Hz, 1H), 7.66 (dd, $J$ = 8.0 Hz, 1.6 Hz, 1H), 7.72-7.77 (m, 3H), 7.83-7.87 (m, 4H) ppm. $^{13}$C NMR (100 MHz, $CDCl_3$): $\delta$ = 51.3, 65.8, 66.1, 69.4, 69.8, 70.8, 70.9, 74.0, 85.2, 119.8, 119.9, 120.0, 120.08, 120.10, 120.18, 120.19, 120.5, 121.5, 124.0, 124.08, 124.12, 124.25, 124.34, 125.5, 127.68, 127.72, 127.8, 127.89, 127.90, 127.93, 127.99, 128.02, 128.1, 128.2, 128.9, 133.5, 134.0, 134.2, 139.9, 140.5, 140.7, 141.3, 141.35, 141.41, 141.7, 141.8, 141.86, 141.89, 148.3, 148.5, 148.56, 148.62, 149.2, 149.3, 149.7, 171.4 ppm. ESI-HRMS: calcd for $C_{66}H_{42}FeO_2SNa$ $[M+Na]^+$ 977.2147, found 977.2150.

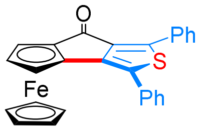


**1,3-Diphenyl-7*H*-ferrocene[1',2':3,4]cyclopenta[1,2-*c*]thiophen-7-one (7a)**

Following the general procedure for synthesis of extended π-conjugated ferrocenes, 4 M of NaOH solution (5 mL) was added to the solution of the thienylferrocene **6a** (239.2 mg, 0.5 mmol) and EtOH (15 mL) in the first step. Then, oxalyl chloride (68 $\mu$L,

0.8 mmol) and $AlCl_3$ (266.7 mg, 2.0 mmol) was used respectively in the next steps. Compound **7a** was obtained as an orange solid (141.8 mg, 64% yield) *via* column chromatography on silica gel (petroleum ether/ethyl acetate = 20/1, v/v). M.p.: 181-184 °C. $^1H$ NMR (400 MHz, $CDCl_3$): $\delta$ = 3.92 (s, 5H), 4.68 (br, 1H), 5.07 (br, 1H), 5.14 (br, 1H), 7.36 (t, $J$ = 7.2 Hz, 1H), 7.45 (t, $J$ = 7.6 Hz, 2H), 7.50-7.55 (m, 2H), 7.61 (t, $J$ = 7.6 Hz, 1H), 7.71 (d, $J$ = 7.6 Hz, 2H), 8.00 (d, $J$ = 8.0 Hz, 1H), 8.46 (d, $J$ = 8.0 Hz, 1H) ppm. $^{13}C$ NMR (100 MHz, $CDCl_3$): $\delta$ = 69.3, 69.7, 71.0, 72.0, 83.1, 83.6, 124.1, 125.9, 127.9, 128.4, 128.9, 129.2, 130.6, 131.9, 132.4, 133.5, 135.0, 136.1, 137.7, 143.4, 194.2 ppm. ESI-HRMS: calcd for $C_{27}H_{18}FeOSNa$ $[M+Na]^+$ 469.0320, found 469.0318.

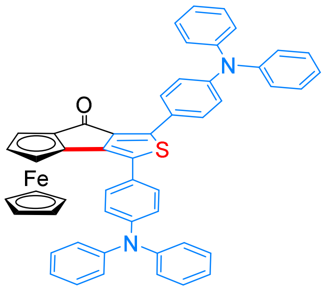


**1,3-Di(4-diphenylaminophenyl)-7*H*-ferrocene[1',2':3,4]cyclopenta[1,2-*c*]thiophen-7-one (7b)**

Following the general procedure for synthesis of extended π-conjugated ferrocenes, compound **7b** was obtained as a yellow solid (79.4 mg, 34% yield) *via* column chromatography on silica gel (petroleum ether/ethyl acetate/dichloromethane = 20/1/2, v/v/v). M.p.: 99-102 °C. $^1H$ NMR (400 MHz, $CDCl_3$): $\delta$ = 3.93 (s, 5H), 4.64 (br, 1H), 5.02 (br, 1H), 5.05 (br, 1H), 7.03-7.18 (m, 15H), 7.27-7.32 (m, 8H), 7.38 (br, 1H), 7.55 (d, $J$ = 8.8 Hz, 2H), 7.83 (d, $J$ = 8.8 Hz, 1H), 8.10 (d, $J$ = 2.4 Hz, 1H) ppm. $^{13}C$ NMR (100 MHz, $CDCl_3$): $\delta$ = 69.0, 69.7, 71.0, 71.9, 83.2, 83.7, 123.0, 123.3, 123.4, 123.5, 123.8, 124.8, 124.9, 126.6, 127.4, 129.5, 129.7, 130.1, 135.5, 136.1, 136.2, 142.2, 147.2, 147.4, 147.5, 147.9, 193.9 ppm. ESI-HRMS: calcd for $C_{51}H_{36}FeN_2OSNa$ $[M+Na]^+$ 803.1790, found 803.1794.

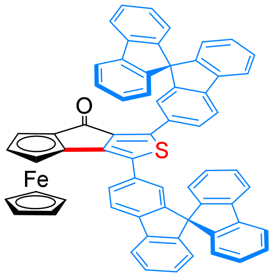


**1,3-Di(9,9'-spirobifluoren-2-yl)-7*H*-ferrocene[1',2':3,4]cyclopenta[1,2-*c*]thiophen-7-one (7c)**

Following the general procedure for synthesis of extended π-conjugated ferrocenes, compound **7c** was obtained as a yellow solid (146.6 mg, 53% yield) *via* column chromatography on silica gel (petroleum ether/ethyl acetate/dichloromethane = 10/1/2, v/v/v). M.p.: > 250 °C. $^{1}$H NMR (400 MHz, $CDCl_3$): $\delta$ = 3.85 (s, 5H), 4.61 (br, 1H), 4.95 (br, 1H), 5.08 (br, 1H), 6.66-6.82 (m, 6H), 6.95 (d, $J$ = 1.2 Hz, 1H), 7.06-7.18 (m, 7H), 7.23 (s, 1H), 7.34-7.43 (m, 6H), 7.57 (dd, $J$ = 8.0 Hz, 1.6 Hz, 1H), 7.81 (t, $J$ = 8.0 Hz, 2H), 7.87-7.94 (m, 4H), 7.99 (d, $J$ = 7.6 Hz, 1H), 8.86 (s, 1H) ppm. $^{13}$C NMR (100 MHz, $CDCl_3$): $\delta$ = 66.1, 66.2, 68.6, 69.8, 71.0, 71.9, 83.5, 83.8, 120.2, 120.3, 120.4, 120.6, 121.0, 121.1, 121.6, 123.97, 124.03, 124.1, 124.16, 124.20, 124.27, 124.31, 124.4, 126.3, 127.9, 127.99, 128.03, 128.13, 128.15, 128.20, 128.25, 128.3, 128.8, 131.6, 133.0, 135.9, 136.1, 137.2, 140.5, 140.9, 141.77, 141.83, 141.9, 142.0, 142.2, 143.0, 148.1, 148.3, 148.4, 148.5, 149.40, 149.41, 149.6, 152.9, 194.8 ppm. ESI-HRMS: calcd for $C_{65}H_{38}FeOSNa$ $[M+Na]^+$ 945.1885, found 945.1882.

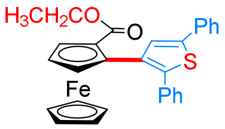


**Ethyl 2-(2,5-diphenylthien-3-yl)ferrocene carboxylate (10)**

M.p.: 45-46 °C. $^{1}$H NMR (400 MHz, $CDCl_3$): $\delta$ = 1.19 (t, $J$ = 7.2 Hz, 3H), 3.94-4.16 (m, 2H), 4.25 (s, 5H), 4.33 (dd, $J$ = 2.8 Hz, 1.6 Hz, 1H), 4.41 (t, $J$ = 2.8 Hz, 1H), 4.90 (dd, $J$ = 2.4 Hz, 1.6 Hz, 1H), 7.16-7.24 (m, 5H), 7.30-7.35 (m, 1H), 7.44 (t, $J$ = 7.6 Hz, 2H), 7.69-7.72 (m, 2H), 7.73 (s, 1H) ppm. $^{13}$C NMR (100 MHz, $CDCl_3$): $\delta$ = 14.5, 60.2, 69.9, 70.5, 70.8, 71.1, 74.5, 85.6, 125.8, 127.2, 127.6, 128.3, 128.7, 129.1, 129.5, 133.5,

134.51, 134.55, 140.0, 140.4, 171.1 ppm. ESI-HRMS: calcd for $C_{29}H_{25}FeO_2S$ $[M+H]^+$ 493.0919, found 493.0921.

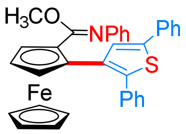


**Methyl *N*-phenyl-2-(2,5-diphenylthien-3-yl)ferrocene carbimidate (11)**

M.p.: 169-173 °C. $^1$H NMR (400 MHz, $CDCl_3$): $\delta$ = 3.88 (s, 3H), 4.02 (dd, $J$ = 2.4 Hz, 1.6 Hz, 1H), 4.09 (t, $J$ = 2.4 Hz, 1H), 4.18 (s, 5H), 4.18-4.19 (m, 1H), 6.38 (d, $J$ = 7.2 Hz, 2H), 6.80 (t, $J$ = 7.6 Hz, 1H), 6.87-6.90 (m, 2H), 7.03 (t, $J$ = 7.6 Hz, 2H), 7.18-7.20 (m, 3H), 7.34 (t, $J$ = 7.6 Hz, 1H), 7.46 (t, $J$ = 8.0 Hz, 2H), 7.57 (s, 1H), 7.70 (dd, $J$ = 8.4 Hz, 1.2 Hz, 2H) ppm. $^{13}$C NMR (100 MHz, $CDCl_3$): $\delta$ = 54.1, 68.2, 70.7, 70.9, 71.2, 76.1, 82.7, 121.4, 122.6, 125.7, 127.3, 127.7, 128.1, 128.2, 128.5, 129.17, 129.18, 134.1, 134.2, 134.6, 138.6, 140.9, 148.6, 160.7 ppm. ESI-HRMS: calcd for $C_{34}H_{28}FeNOS$ $[M+H]^+$ 554.1236, found 554.1232.

## XI. References


1 Y.-H. Liu, P.-X. Li, Q.-J. Yao, Z.-Z. Zhang, D.-Y. Huang, M. D. Le, H. Song, L. Liu and B.-F. Shi, *Org. Lett.*, 2019, **21**, 1895–1899.

2 T. Wang, J. Du, S. Ye, L. Tan and J. Fu, *ACS Appl. Mater. Interfaces*, 2019, **11**, 4425–4438.

3 G. Zhang, H. Yi, H. Chen, C. Bian, C. Liu and A. Lei, *Org. Lett.*, 2014, **16**, 6156–6159.

4 X. Li, X. Liu, H. Chen, W. Wu, C. Qi and H. Jiang, *Angew. Chem., Int. Ed.*, 2014, **53**, 14485–14489.

5 R. S. Jordan, Y. L. Li, C.-W. Lin, R. D. McCurdy, J. B. Lin, J. L. Brosmer, K. L. Marsh, S. I. Khan, K. N. Houk, R. B. Kaner and Y. Rubin, *J. Am. Chem. Soc.*, 2017, **139**, 15878–15890.

6 T. Yang, W. Moreira, S. A. Nyantakyi, H. Chen, D. Aziz, M.-L. Go and T. Dick, *J. Med. Chem.*, 2017, **60**, 2745–2763.

7 P. Chen, C. Liu, J. Hu, H. Zhang and R. Sun, *J. Organomet. Chem.*, 2018, **854**, 113–121.

## XII. Copies of $^1$H, $^{13}$C and $^1$H-$^1$H NOESY NMR spectra

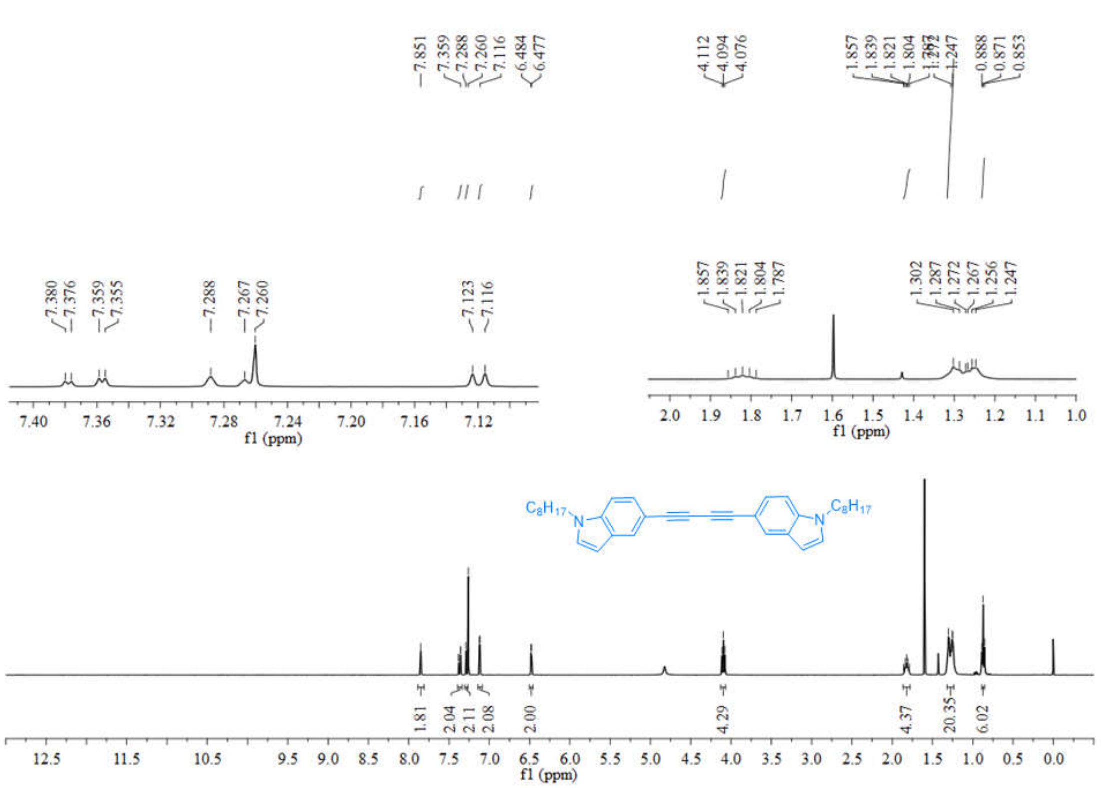

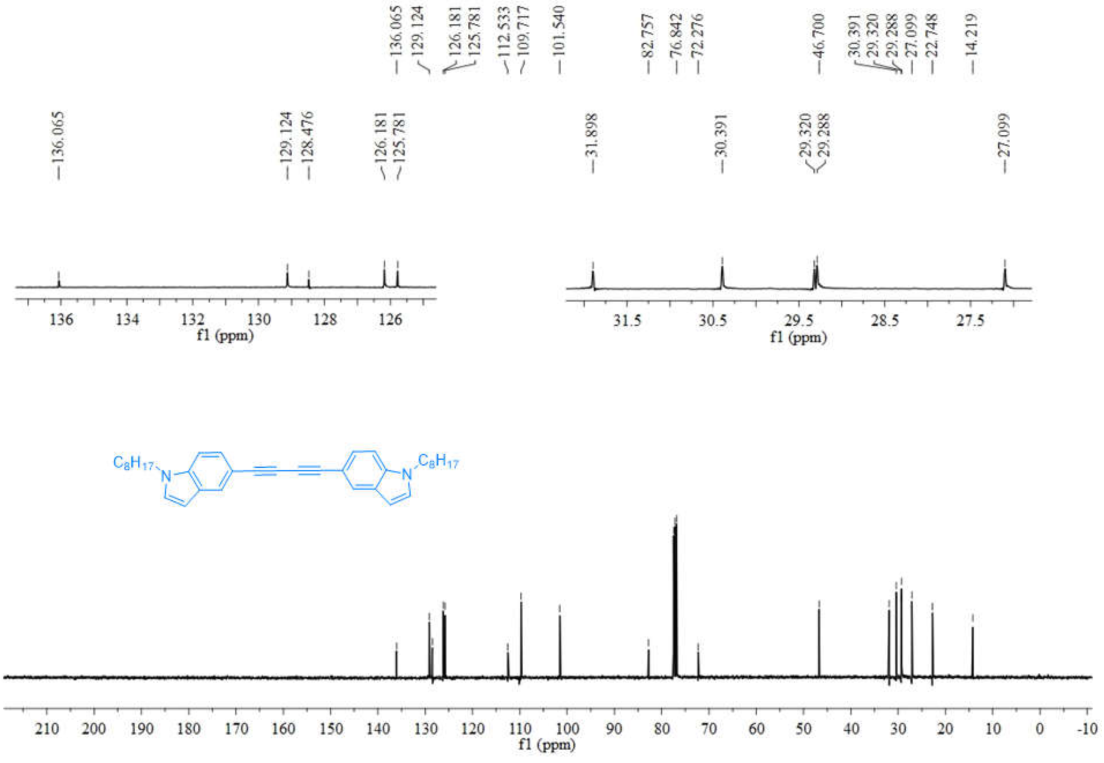

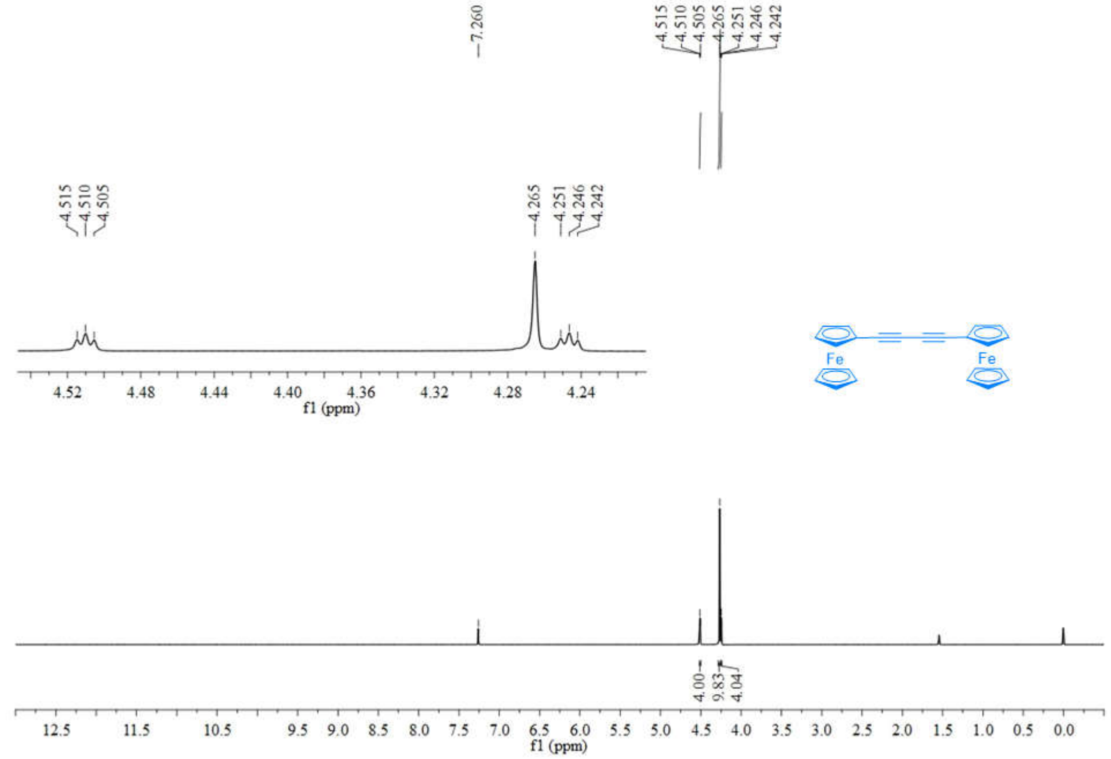
7.260
4.515
4.510
4.505
4.265
4.251
4.246
4.242
Fe
Fe
4.00
9.83
4.04
f1 (ppm)

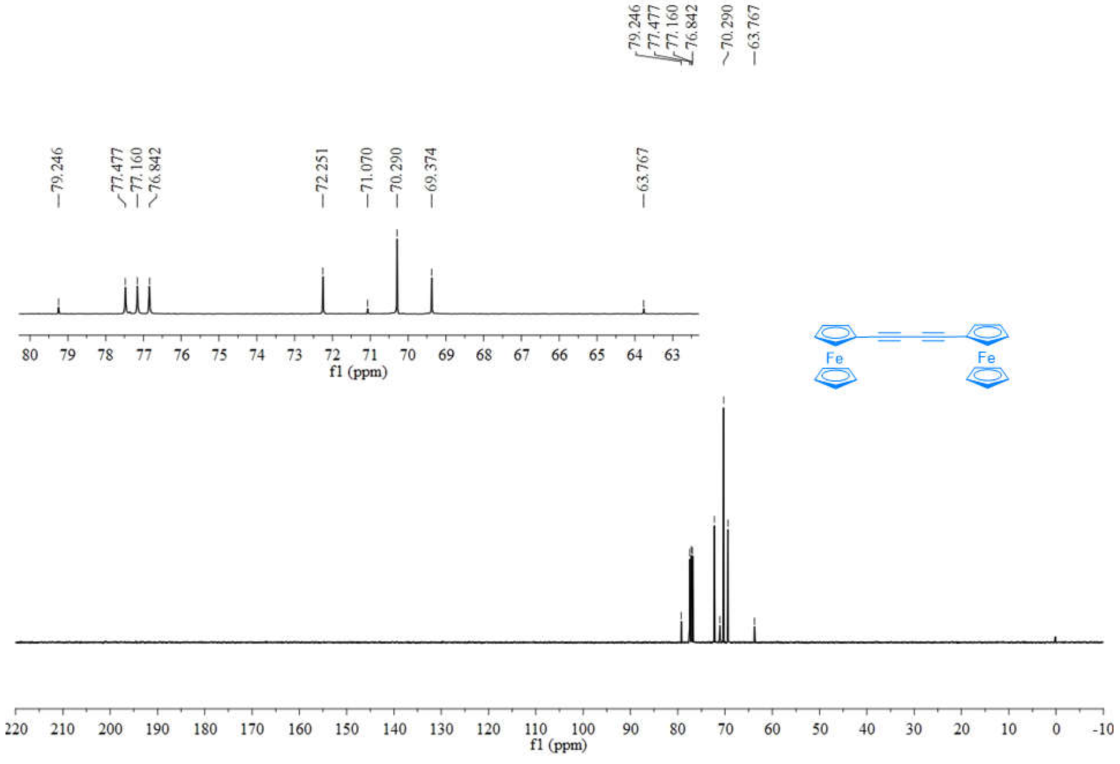
79.246
77.477
77.160
76.842
70.290
63.767
72.251
71.070
69.374
Fe
Fe
f1 (ppm)

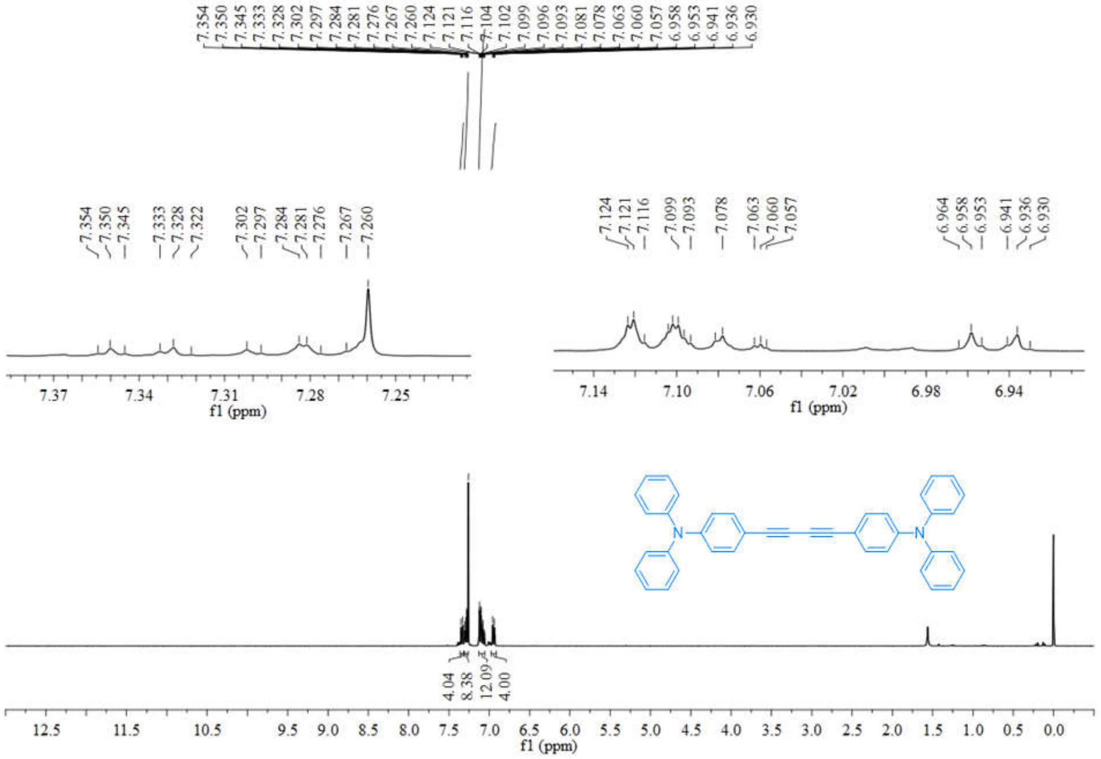
7.354
7.350
7.345
7.333
7.328
7.322
7.302
7.297
7.284
7.281
7.276
7.267
7.260
7.124
7.121
7.116
7.104
7.102
7.099
7.096
7.093
7.081
7.078
7.063
7.060
7.057
6.958
6.953
6.941
6.936
6.930
6.964
N
4.04
8.38
12.09
4.00
f1 (ppm)

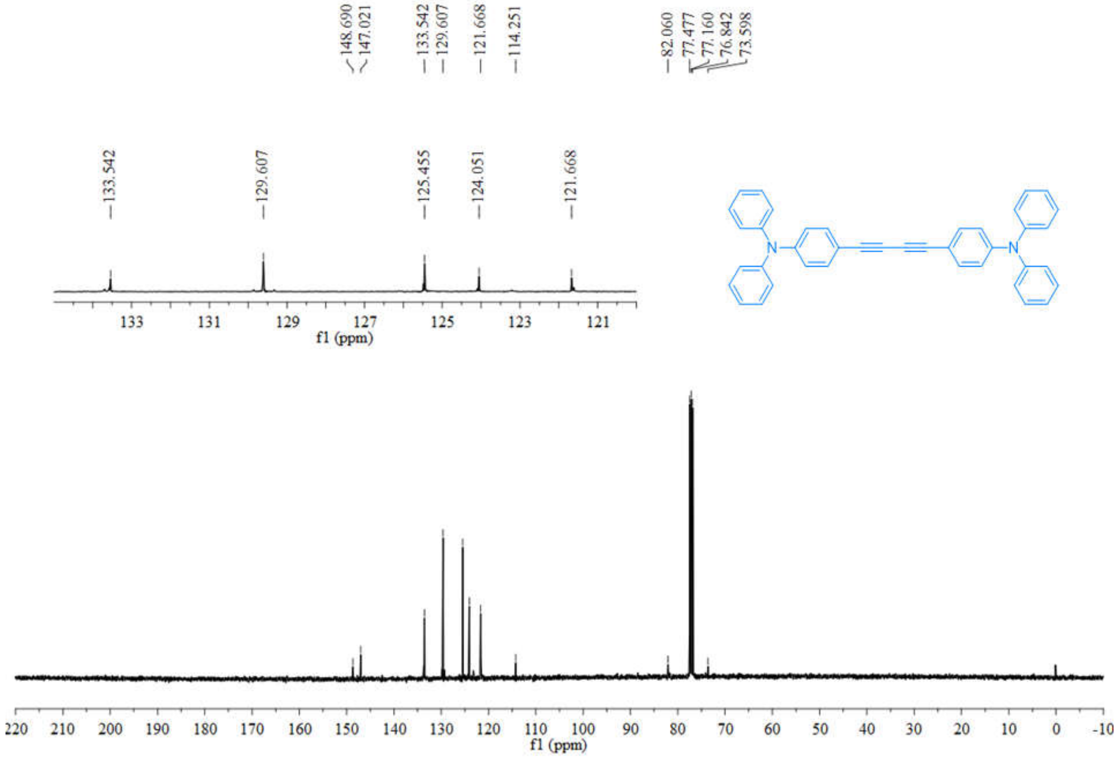
148.690
147.021
133.542
129.607
121.668
114.251
82.060
77.477
77.160
76.842
73.598
125.455
124.051
N
f1 (ppm)

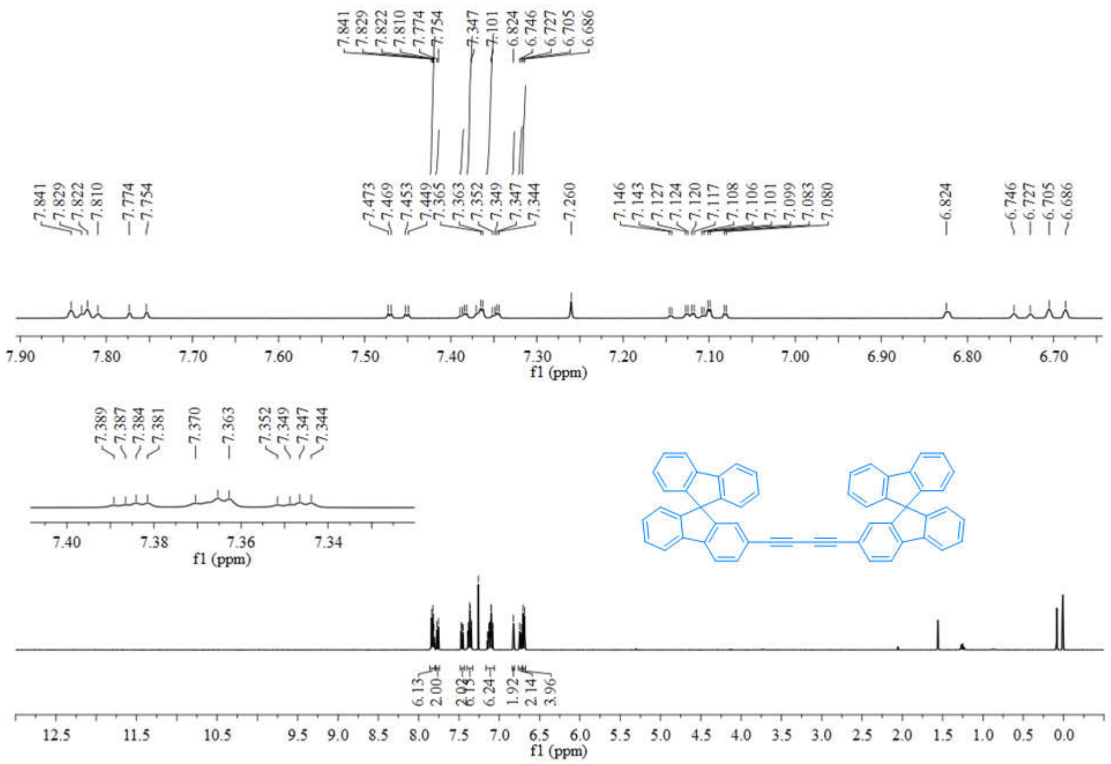
f1 (ppm)

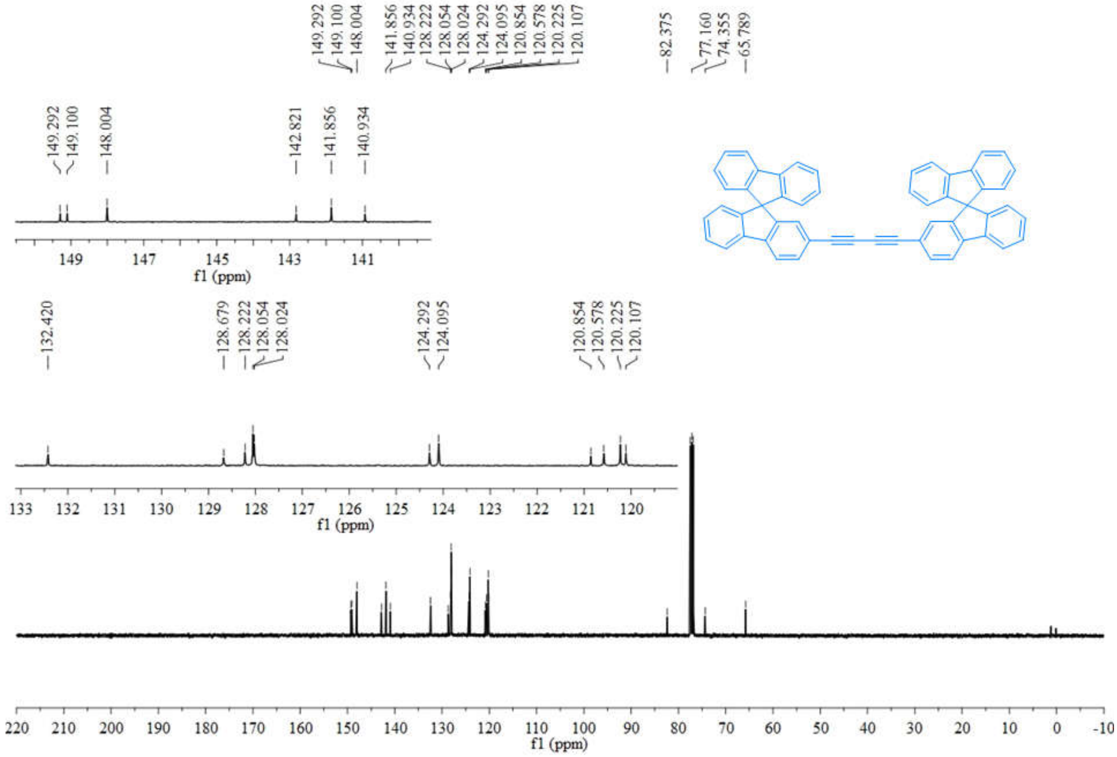
f1 (ppm)

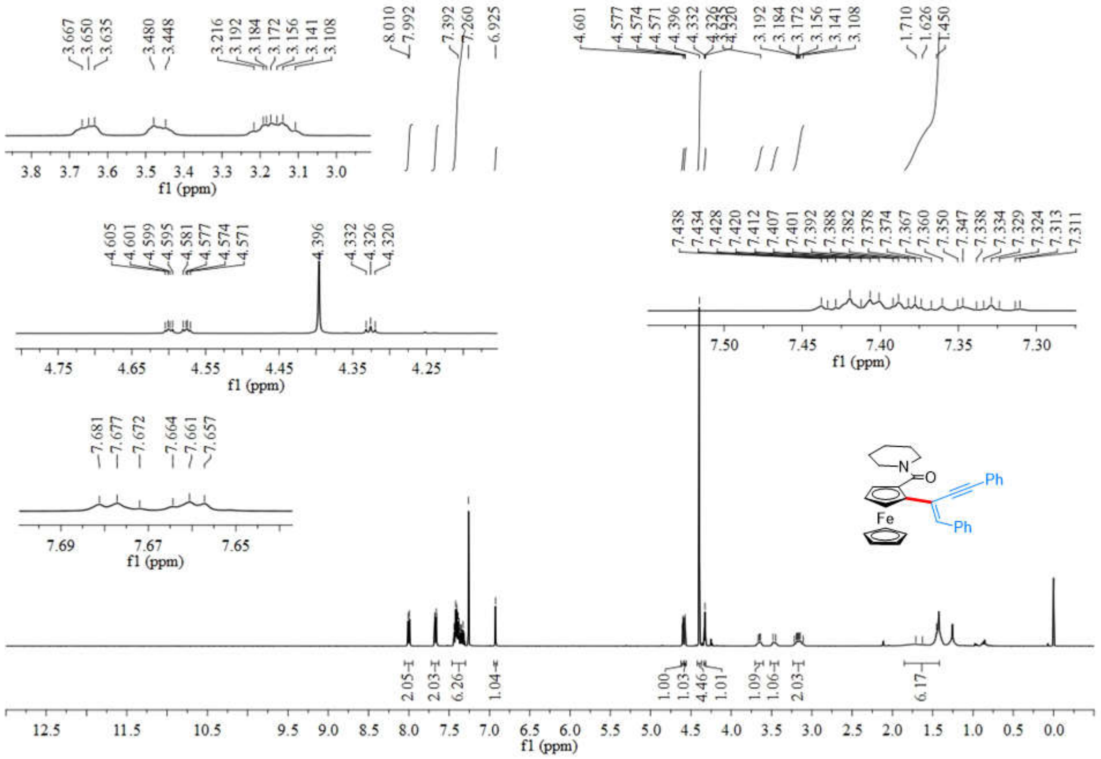

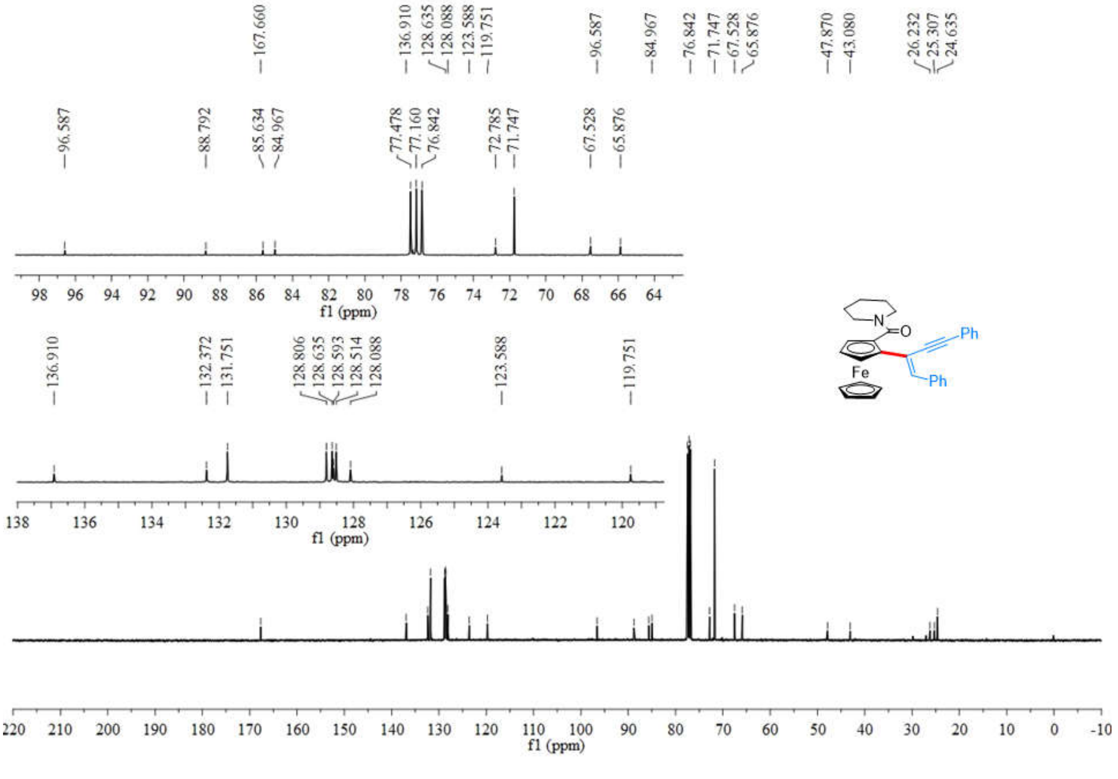

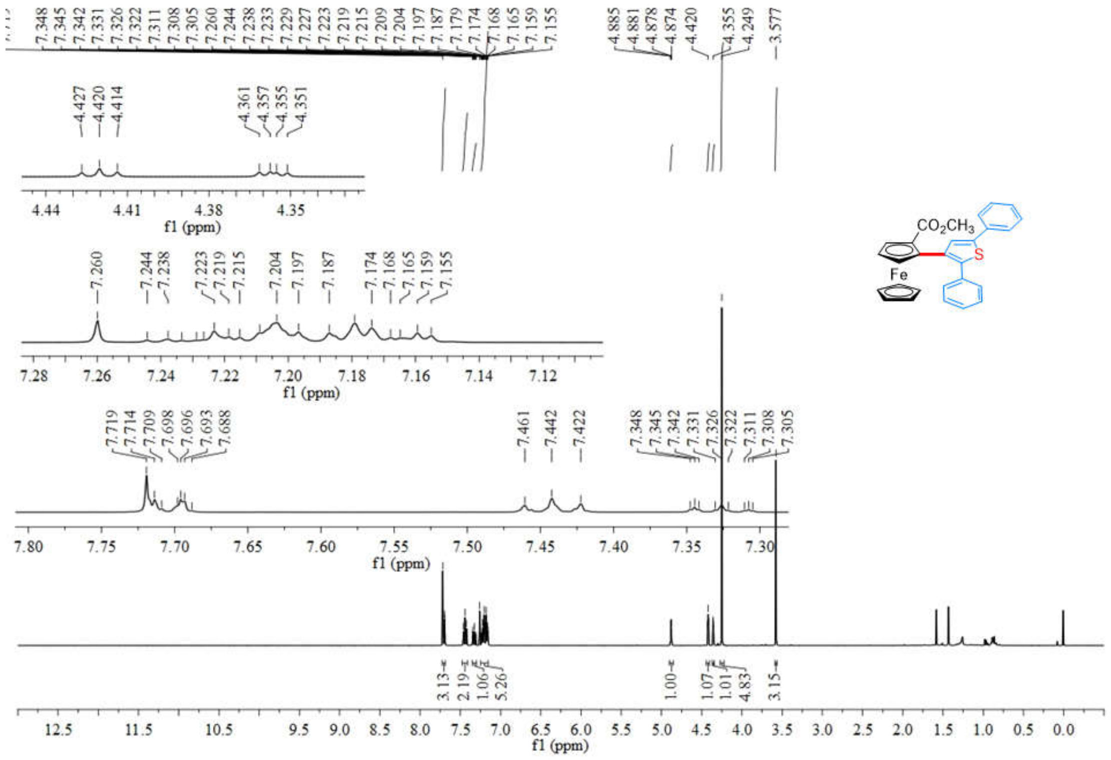

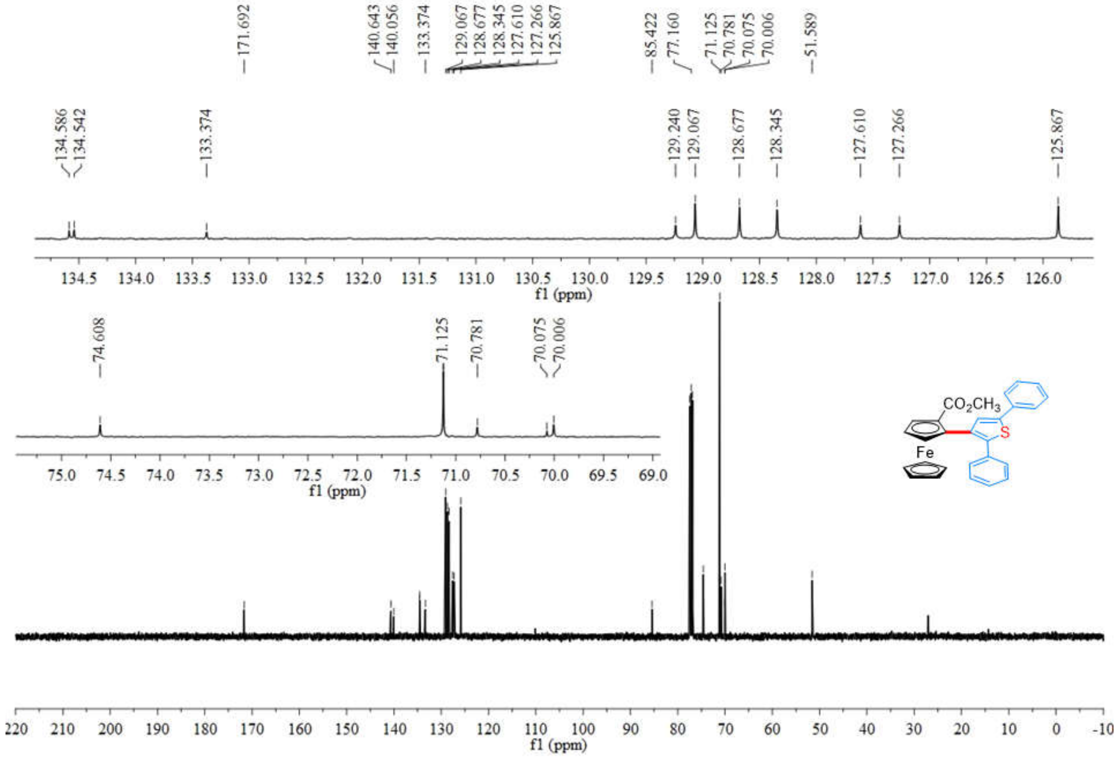

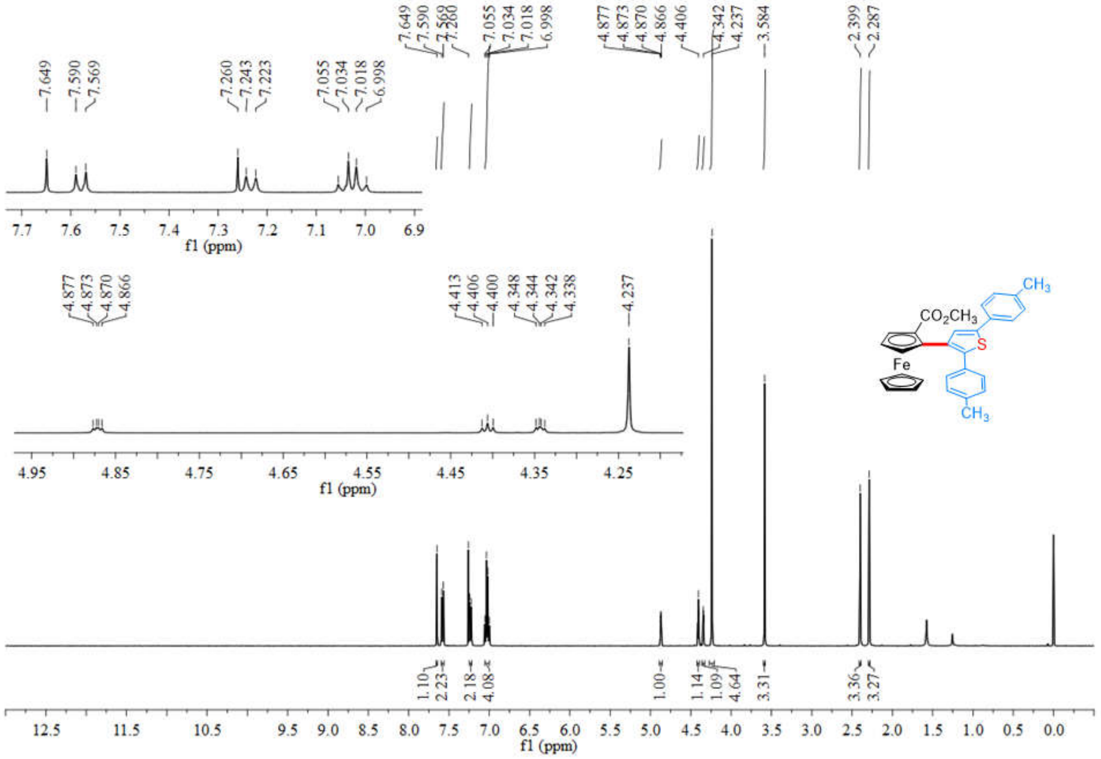
7.649 7.590 7.569 7.260 7.243 7.223 7.055 7.034 7.018 6.998
4.877 4.873 4.870 4.866 4.406 4.342 4.237 3.584 2.399 2.287
4.413 4.406 4.400 4.348 4.344 4.342 4.338 4.237
$CO_2CH_3$
Fe
S
$CH_3$
$CH_3$
1.10 2.23 2.18 4.08 1.00 1.14 1.09 4.64 3.31 3.36 3.27
f1 (ppm)

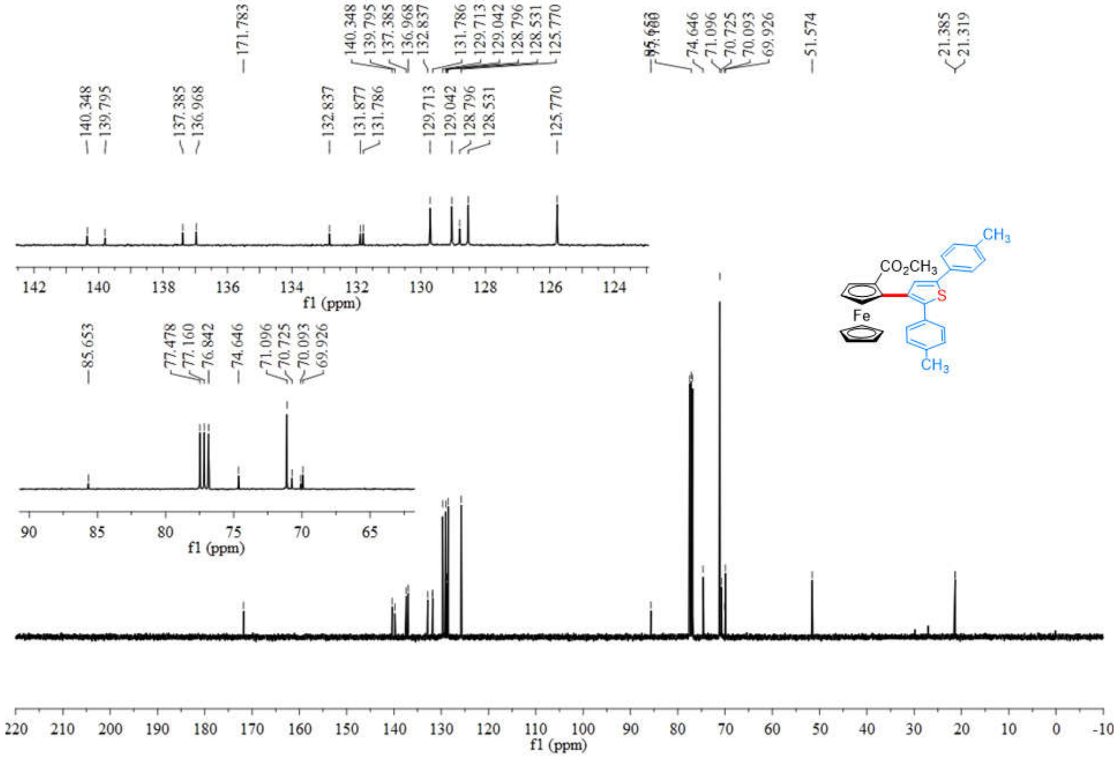
171.783
140.348 139.795 137.385 136.968 132.837 131.786 129.713 129.042 128.796 128.531 125.770
85.653 74.646 71.096 70.725 70.093 69.926 51.574
21.385 21.319
140.348 139.795 137.385 136.968 132.837 131.877 131.786 129.713 129.042 128.796 128.531 125.770
85.653 77.478 77.160 76.842 74.646 71.096 70.725 70.093 69.926
$CO_2CH_3$
Fe
S
$CH_3$
$CH_3$
f1 (ppm)

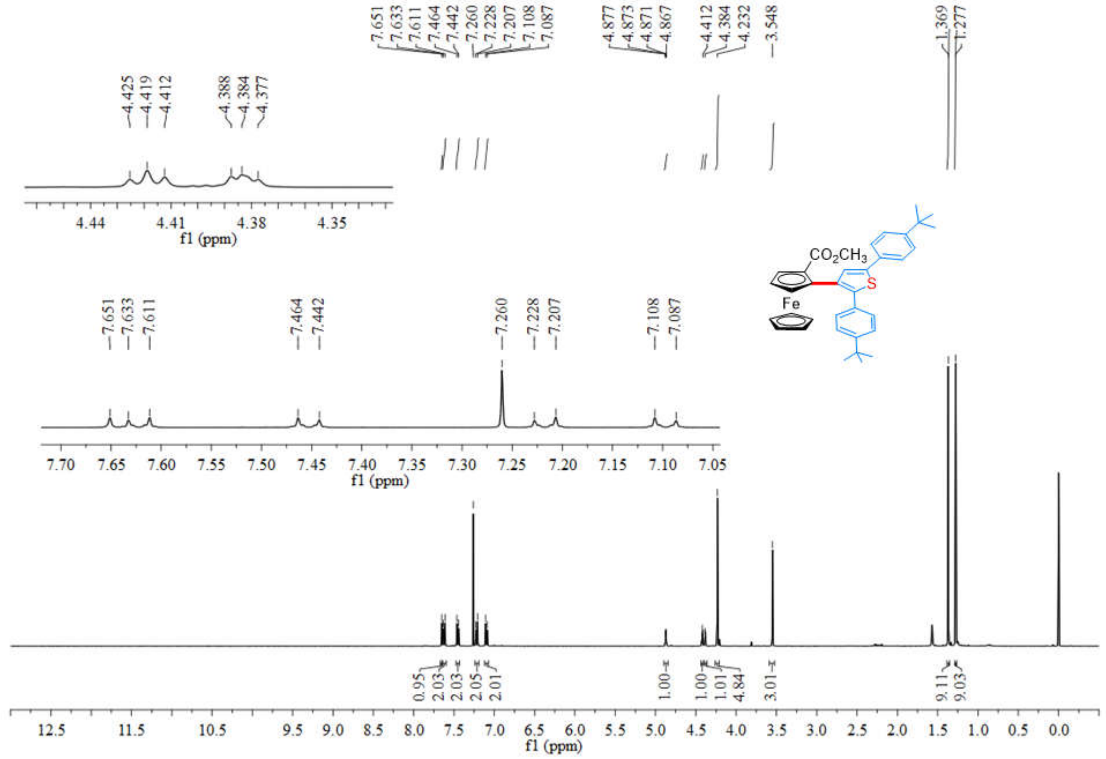
7.651 7.633 7.611 7.464 7.442 7.260 7.228 7.207 7.108 7.087
4.877 4.873 4.871 4.867
4.412 4.384 4.232
3.548
1.369 1.277
4.425 4.419 4.412 4.388 4.384 4.377
f1 (ppm)
CO2CH3
Fe
S
0.95 2.03 2.03 2.05 2.01
1.00 1.00 1.01 4.84 3.01
9.11 9.03
f1 (ppm)

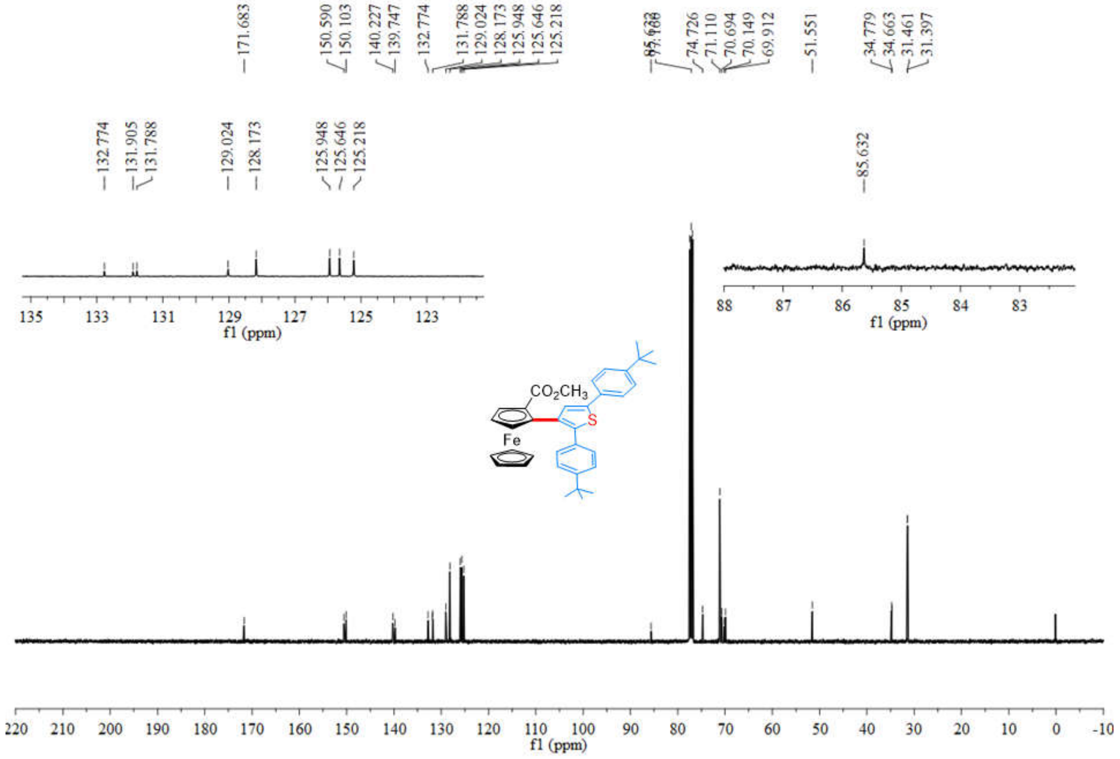
171.683
150.590 150.103 140.227 139.747 132.774 131.788 129.024 128.173 125.948 125.646 125.218
74.726 71.110 70.694 70.149 69.912
51.551
34.779 34.663 31.461 31.397
132.774 131.905 131.788 129.024 128.173 125.948 125.646 125.218
f1 (ppm)
85.632
f1 (ppm)
CO2CH3
Fe
S
f1 (ppm)

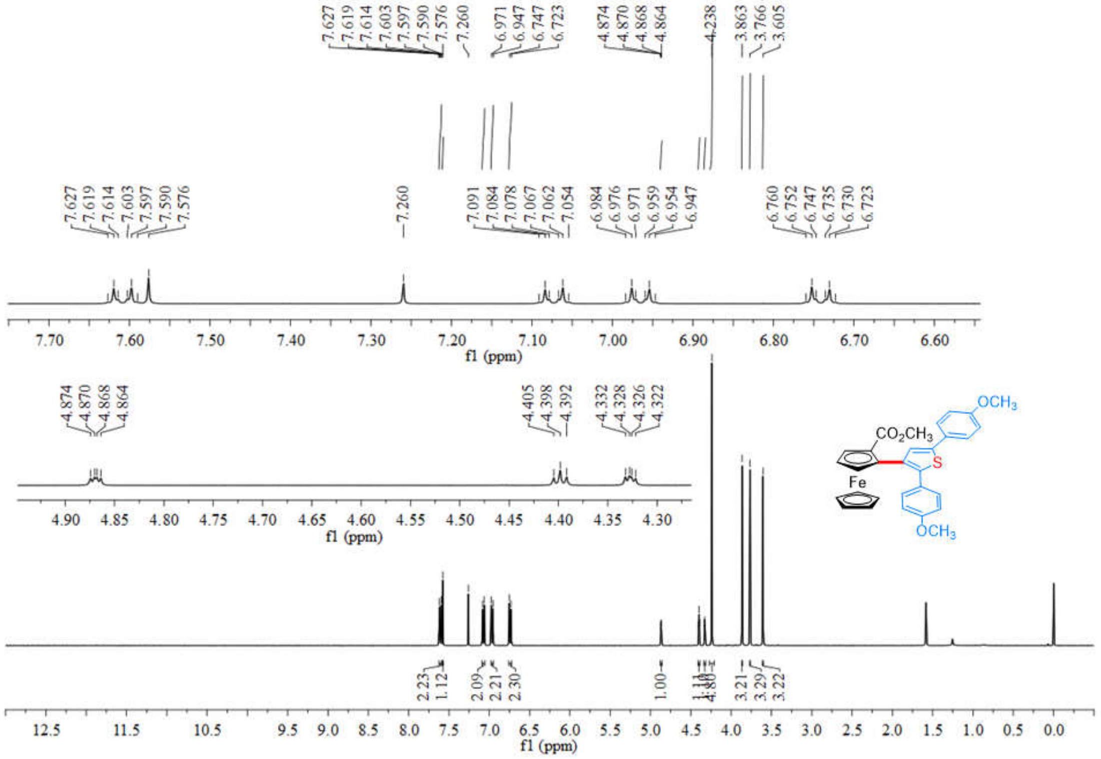
CO2CH3
OCH3
OCH3
Fe
S
f1 (ppm)

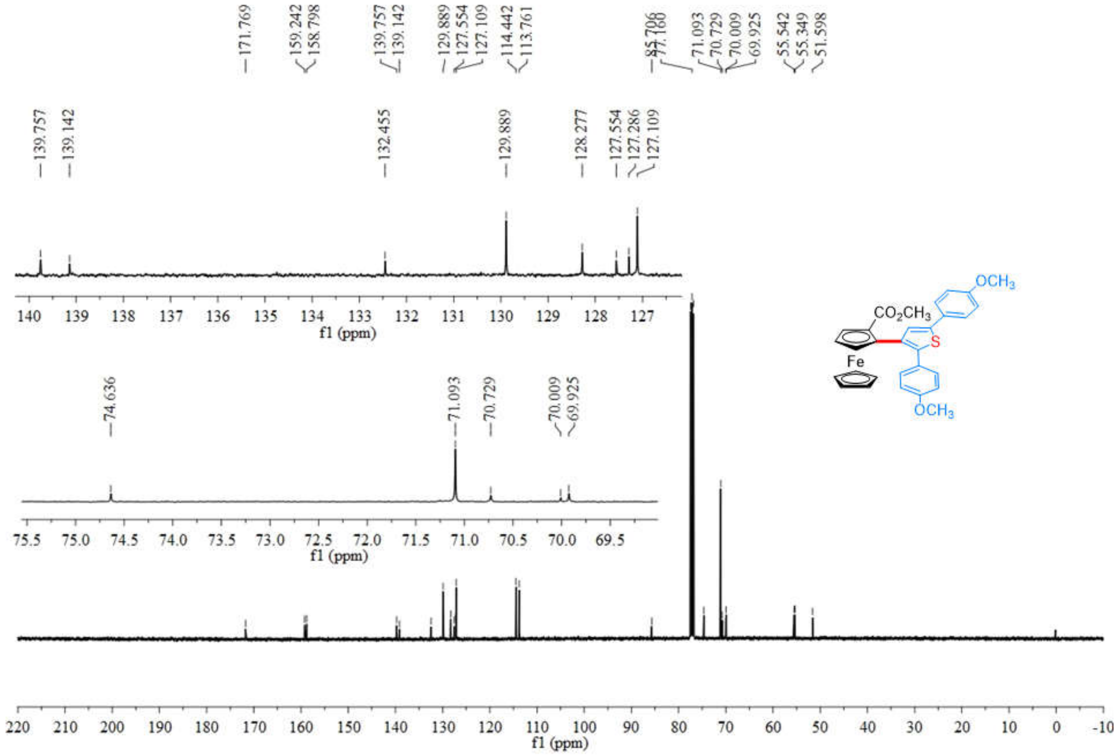
CO2CH3
OCH3
OCH3
Fe
S
f1 (ppm)

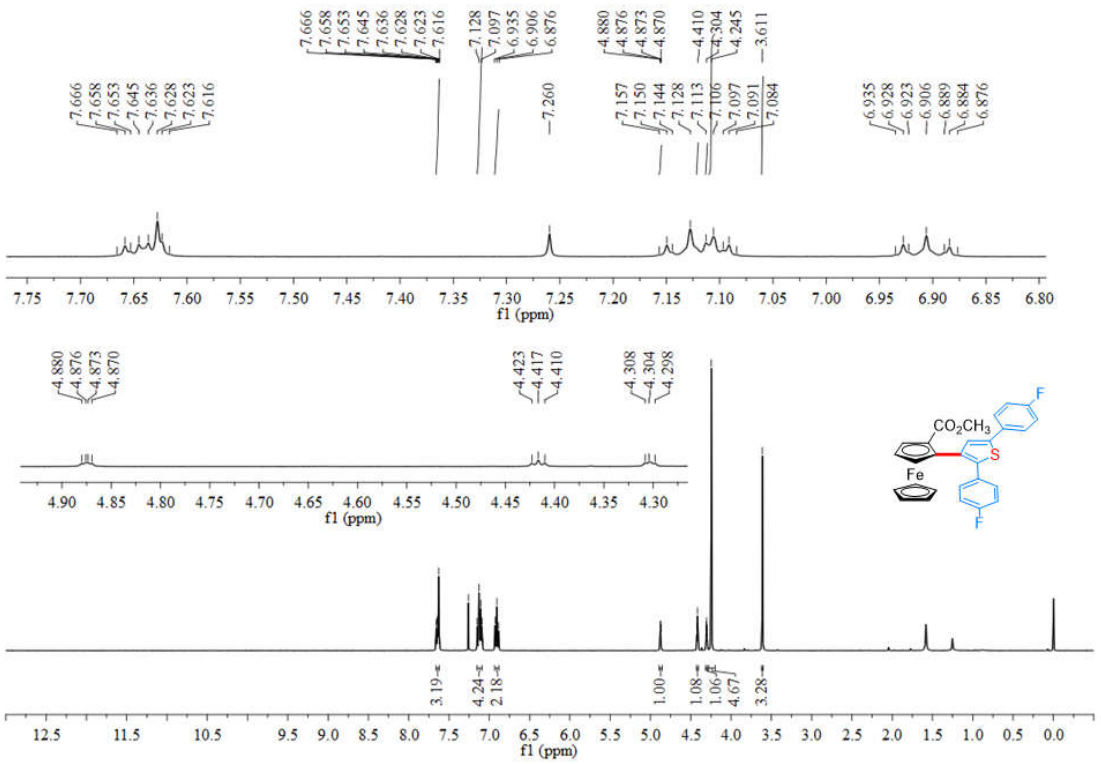
f1 (ppm)
CO2CH3
Fe
S
F
F

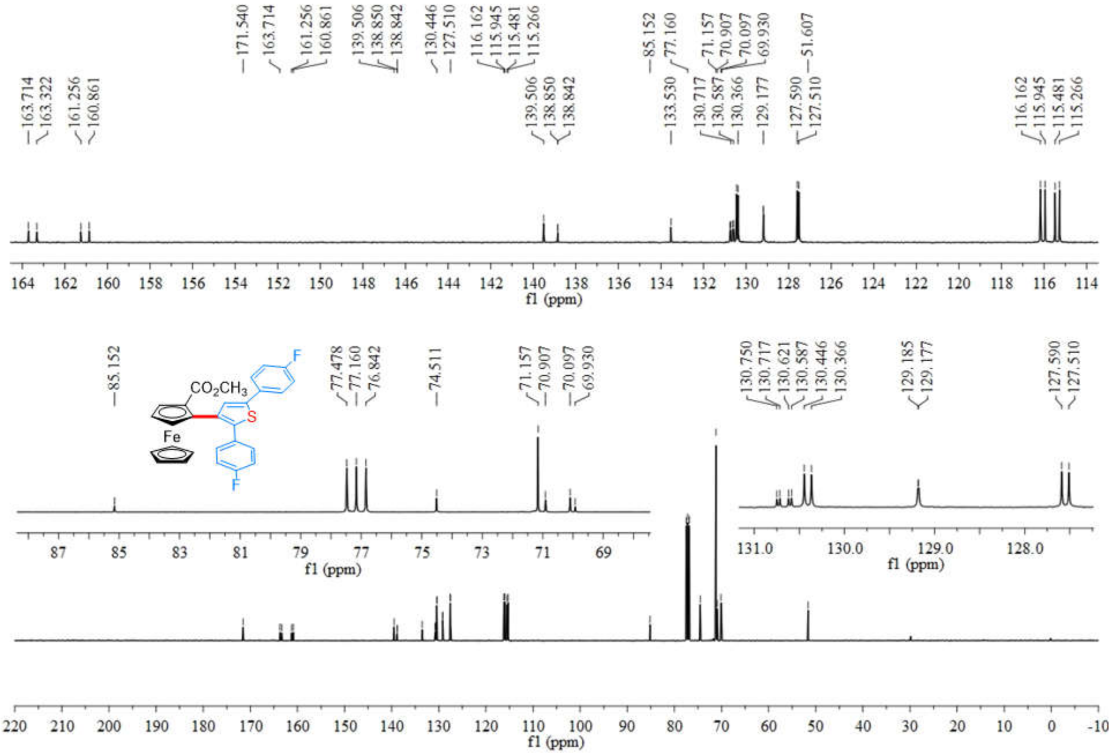
f1 (ppm)
CO2CH3
Fe
S
F
F

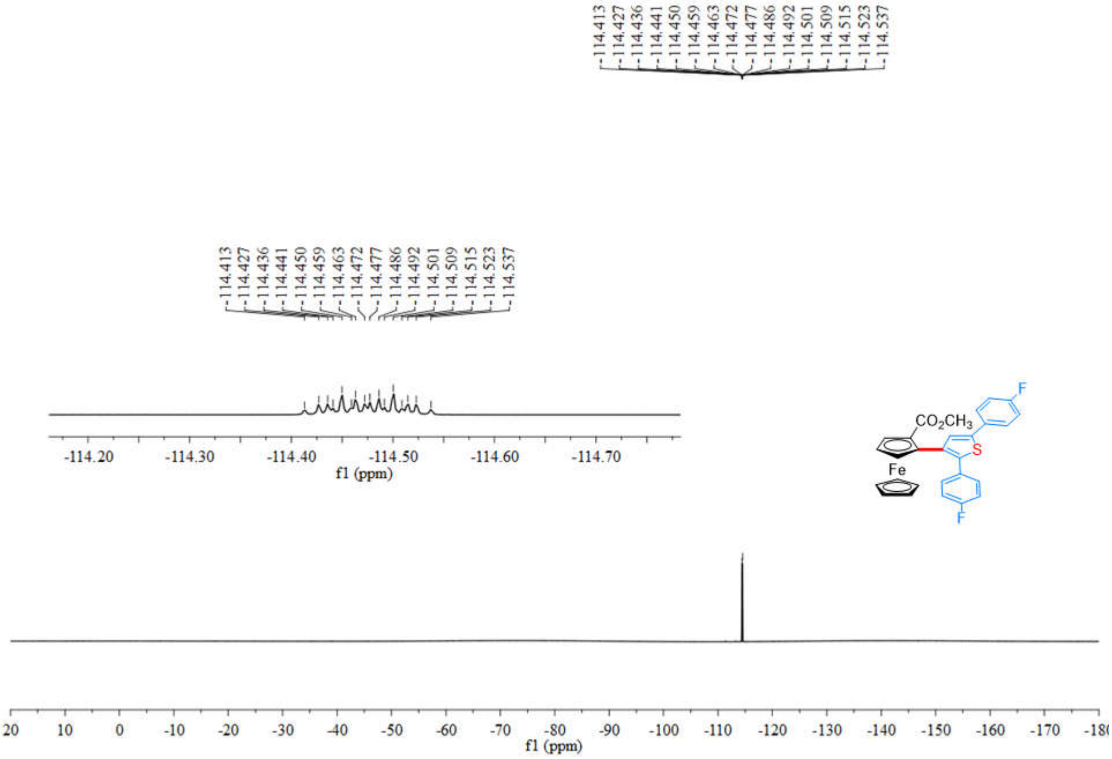
-114.413
-114.427
-114.436
-114.441
-114.450
-114.459
-114.463
-114.472
-114.477
-114.486
-114.492
-114.501
-114.509
-114.515
-114.523
-114.537
-114.20
-114.30
-114.40
-114.50
-114.60
-114.70
f1 (ppm)
20 10 0 -10 -20 -30 -40 -50 -60 -70 -80 -90 -100 -110 -120 -130 -140 -150 -160 -170 -180
f1 (ppm)
CO2CH3
F
S
Fe
F

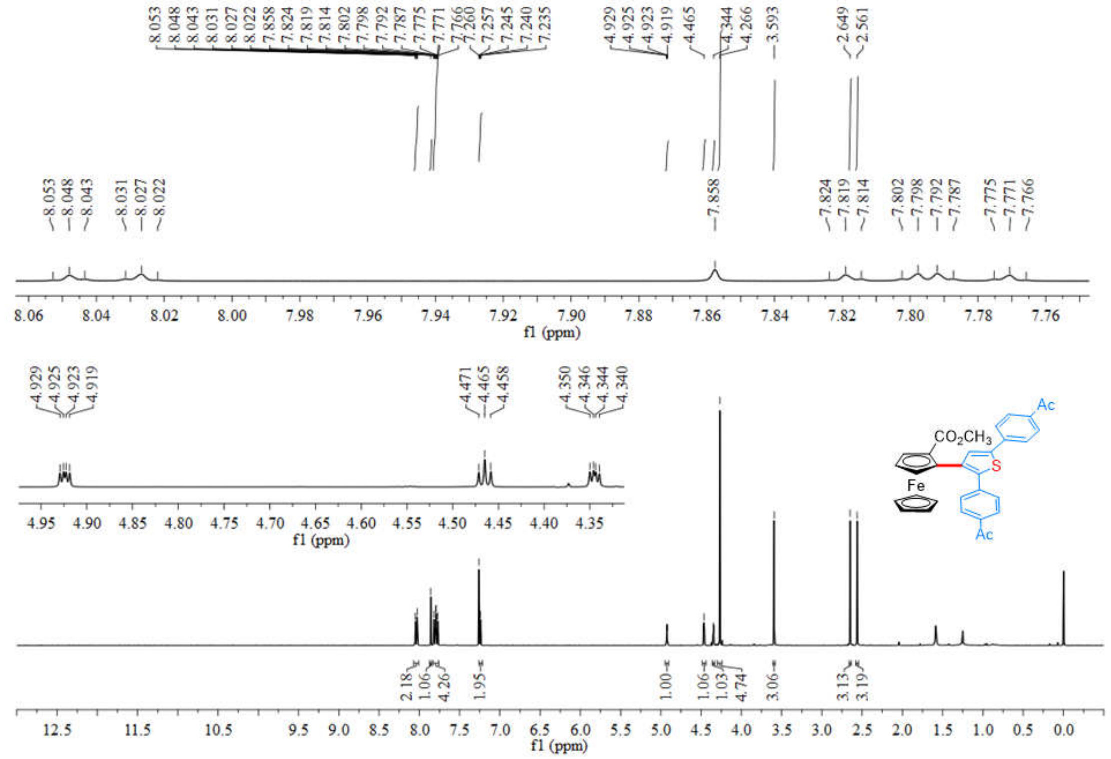

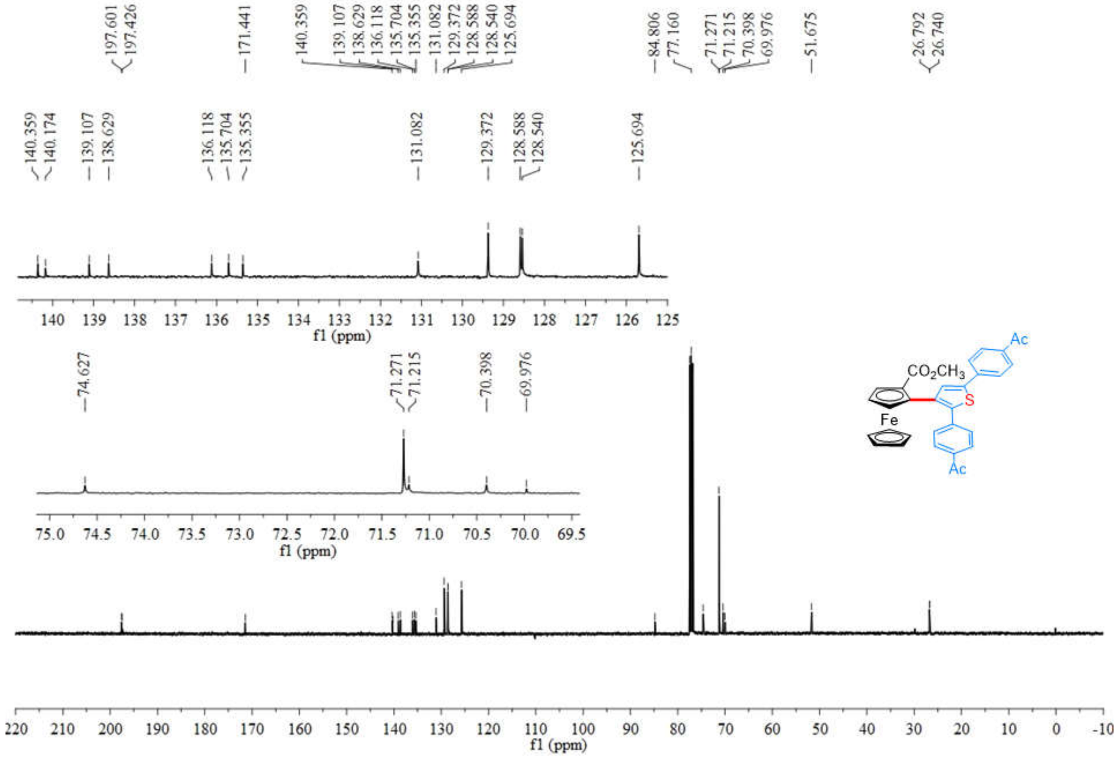

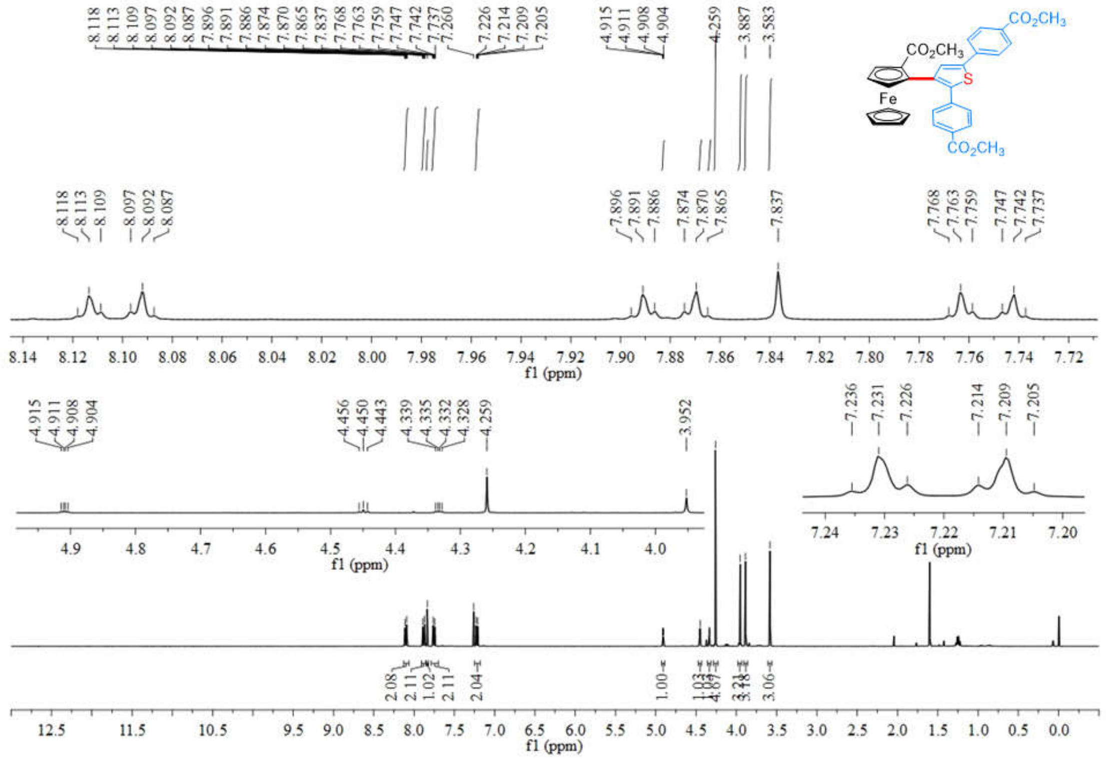
8.118
8.113
8.109
8.097
8.092
8.087
7.896
7.891
7.886
7.874
7.870
7.865
7.837
7.768
7.763
7.759
7.747
7.742
7.737
7.260
7.226
7.214
7.209
7.205
4.915
4.911
4.908
4.904
4.259
3.887
3.583
$CO_2CH_3$
Fe
S
4.456
4.450
4.443
4.339
4.335
4.332
4.328
3.952
7.236
7.231
2.08
2.11
1.02
2.11
2.04
1.00
1.03
3.21
3.18
3.06
f1 (ppm)

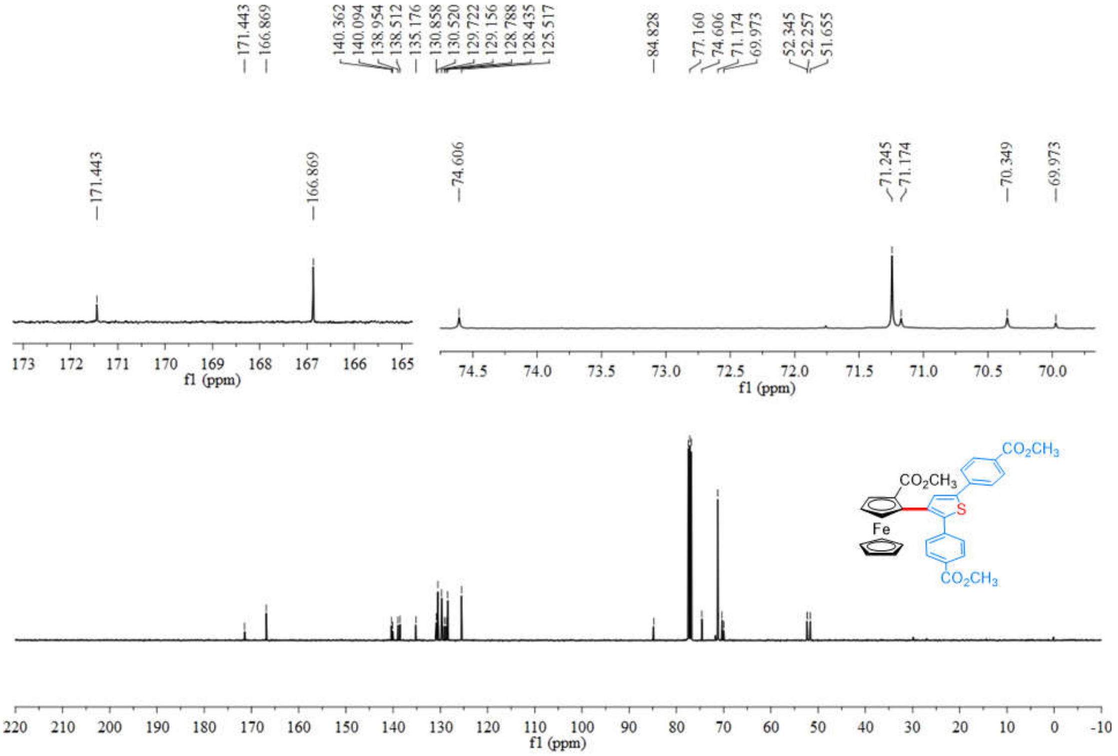
171.443
166.869
140.362
140.094
138.954
138.512
135.176
130.858
130.520
129.722
129.156
128.788
128.435
125.517
84.828
77.160
74.606
71.174
69.973
52.345
52.257
51.655
71.245
70.349
$CO_2CH_3$
Fe
S
f1 (ppm)

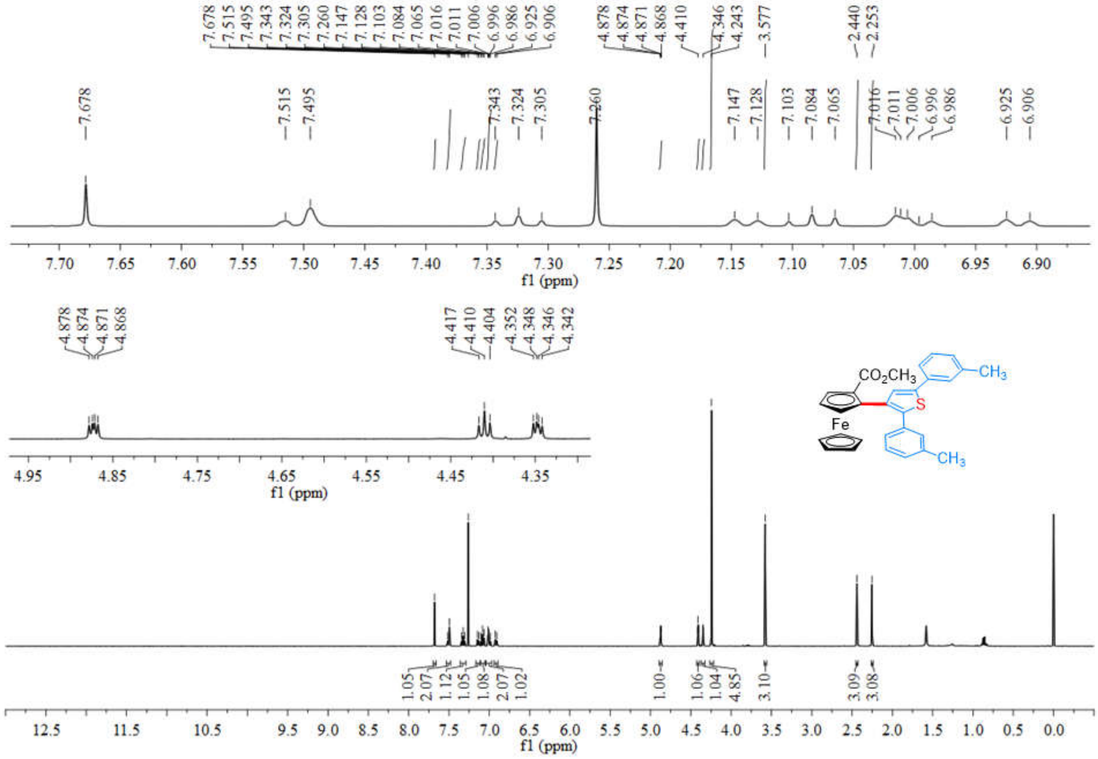
7.678 7.515 7.495 7.343 7.324 7.305 7.260 7.147 7.128 7.103 7.084 7.065 7.016 7.011 7.006 6.996 6.986 6.925 6.906
4.878 4.874 4.871 4.868 4.410 4.346 4.243 3.577 2.440 2.253
4.878 4.874 4.871 4.868 4.417 4.410 4.404 4.352 4.348 4.346 4.342
CO2CH3
Fe
S
CH3
CH3
1.05 2.07 1.12 1.05 1.08 2.07 1.02 1.00 1.06 1.04 4.85 3.10 3.09 3.08
f1 (ppm)

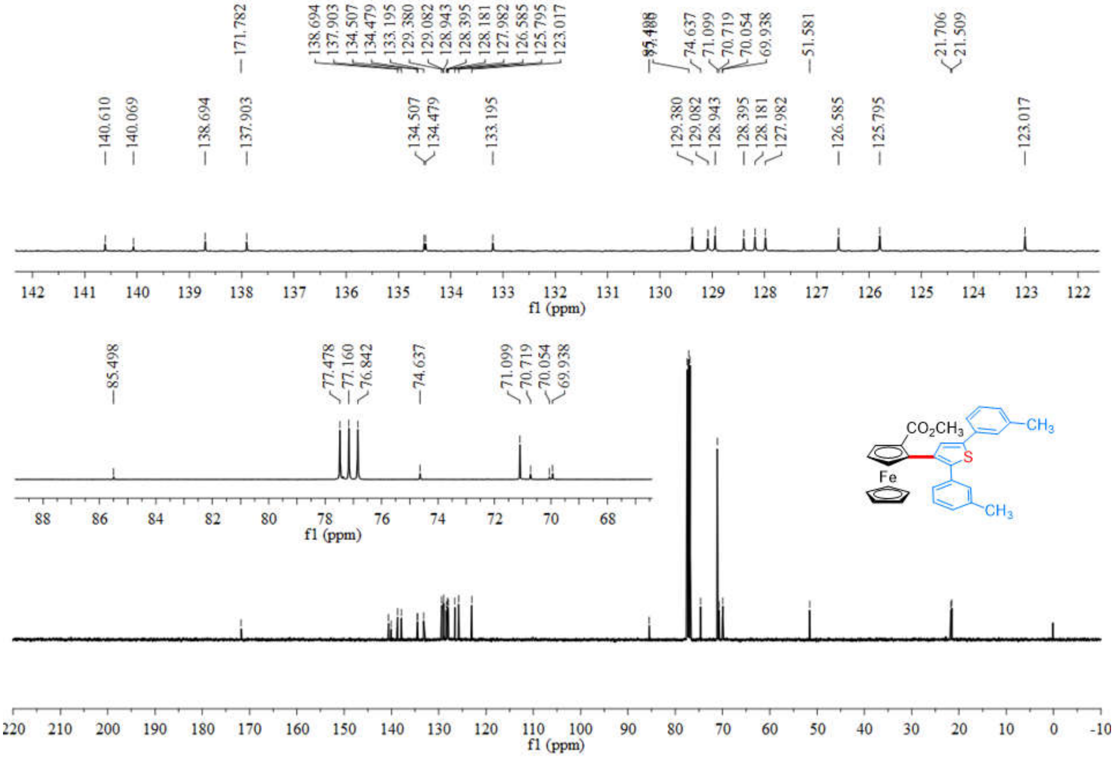
171.782 138.694 137.903 134.507 134.479 133.195 129.380 129.082 128.943 128.395 128.181 127.982 126.585 125.795 123.017 74.637 71.099 70.719 70.054 69.938 51.581 21.706 21.509
140.610 140.069 138.694 137.903 134.507 134.479 133.195 129.380 129.082 128.943 128.395 128.181 127.982 126.585 125.795 123.017
85.498 77.478 77.160 76.842 74.637 71.099 70.719 70.054 69.938
CO2CH3
Fe
S
CH3
CH3
f1 (ppm)

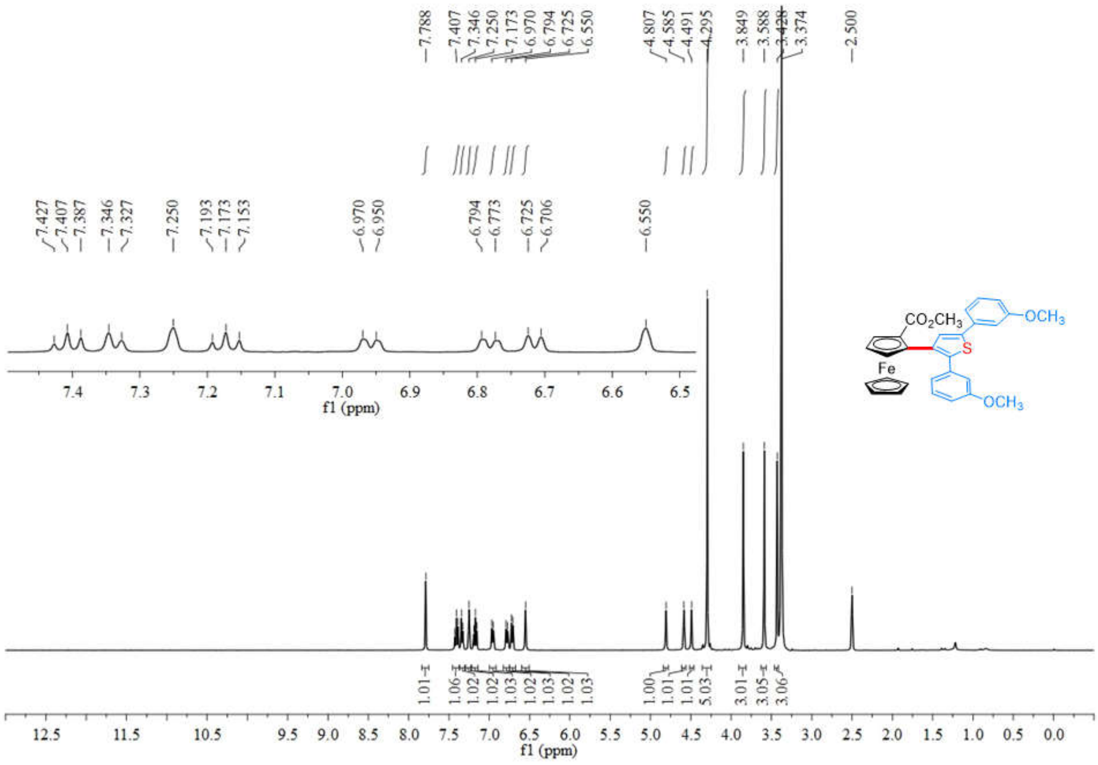
7.788
7.407
7.346
7.250
7.173
6.970
6.794
6.725
6.550
4.807
4.585
4.491
4.295
3.849
3.588
3.428
3.374
2.500
7.427
7.407
7.387
7.346
7.327
7.250
7.193
7.173
7.153
6.970
6.950
6.794
6.773
6.725
6.706
6.550
f1 (ppm)
$CO_2CH_3$
Fe
S
$OCH_3$
$OCH_3$
1.01
1.06
1.02
1.02
1.02
1.03
1.02
1.03
1.02
1.03
1.00
1.01
1.01
5.03
3.01
3.05
3.06
f1 (ppm)

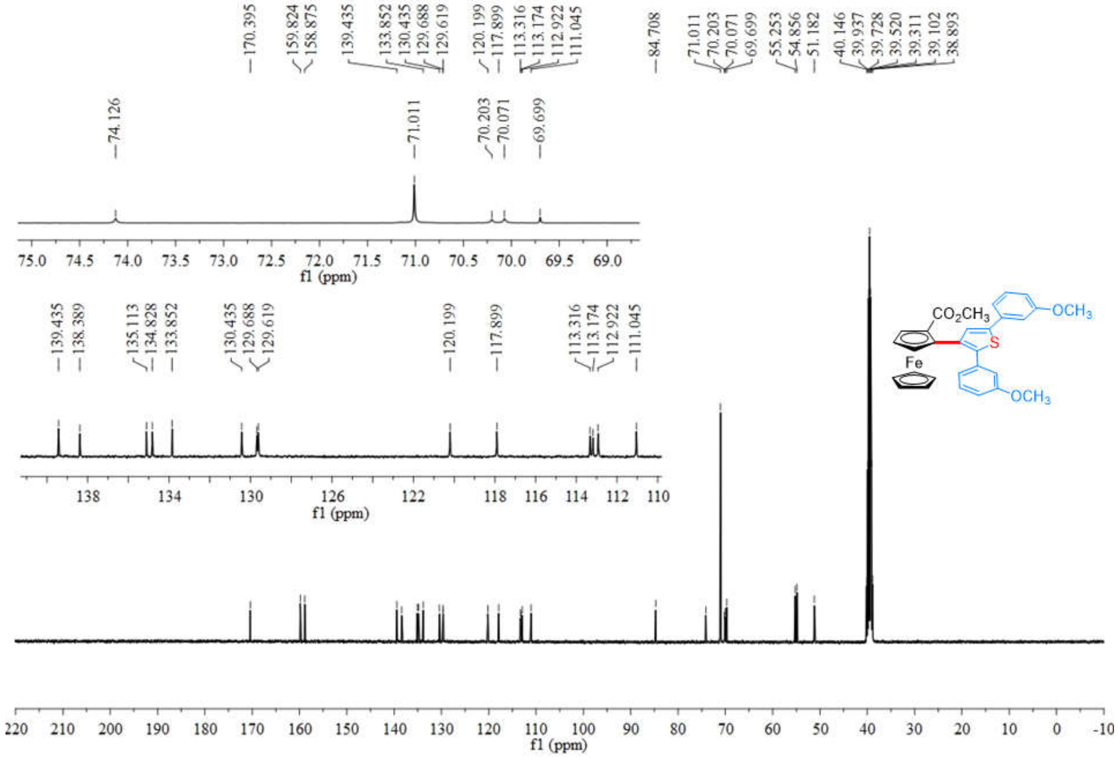
170.395
159.824
158.875
139.435
133.852
130.435
129.688
129.619
120.199
117.899
113.316
113.174
112.922
111.045
84.708
71.011
70.203
70.071
69.699
55.253
54.856
51.182
40.146
39.937
39.728
39.520
39.311
39.102
38.893
74.126
71.011
70.203
70.071
69.699
f1 (ppm)
139.435
138.389
135.113
134.828
133.852
130.435
129.688
129.619
120.199
117.899
113.316
113.174
112.922
111.045
f1 (ppm)
$CO_2CH_3$
Fe
S
$OCH_3$
$OCH_3$
f1 (ppm)

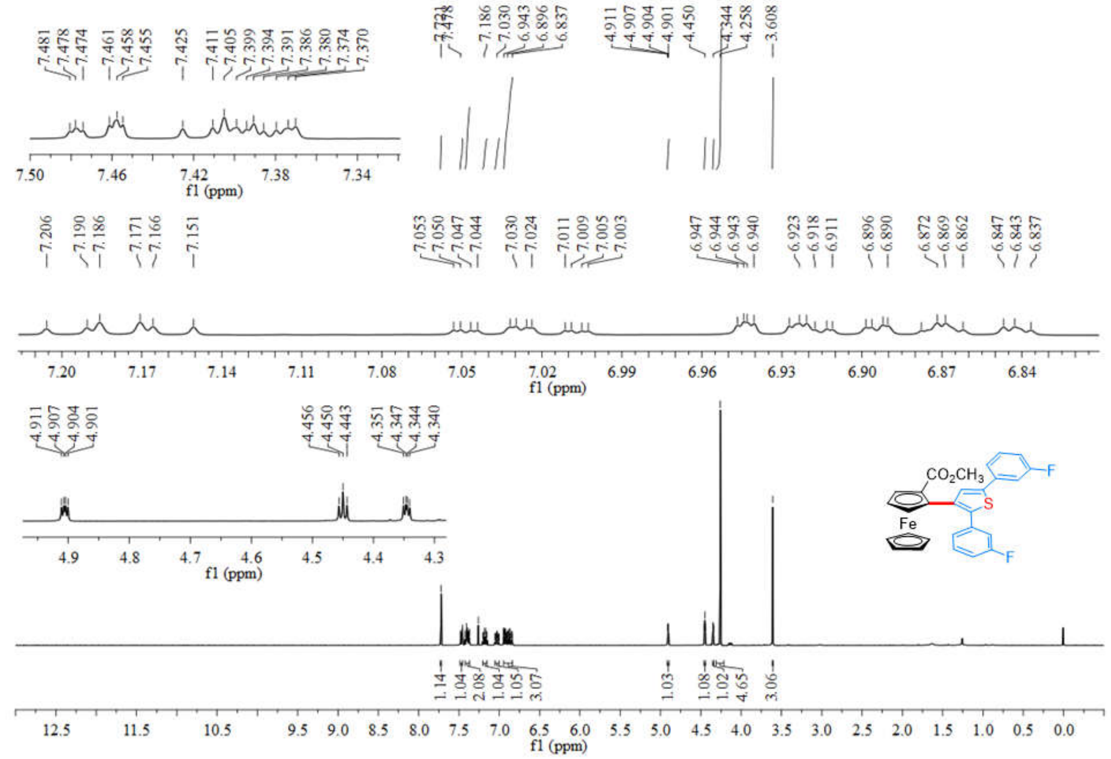

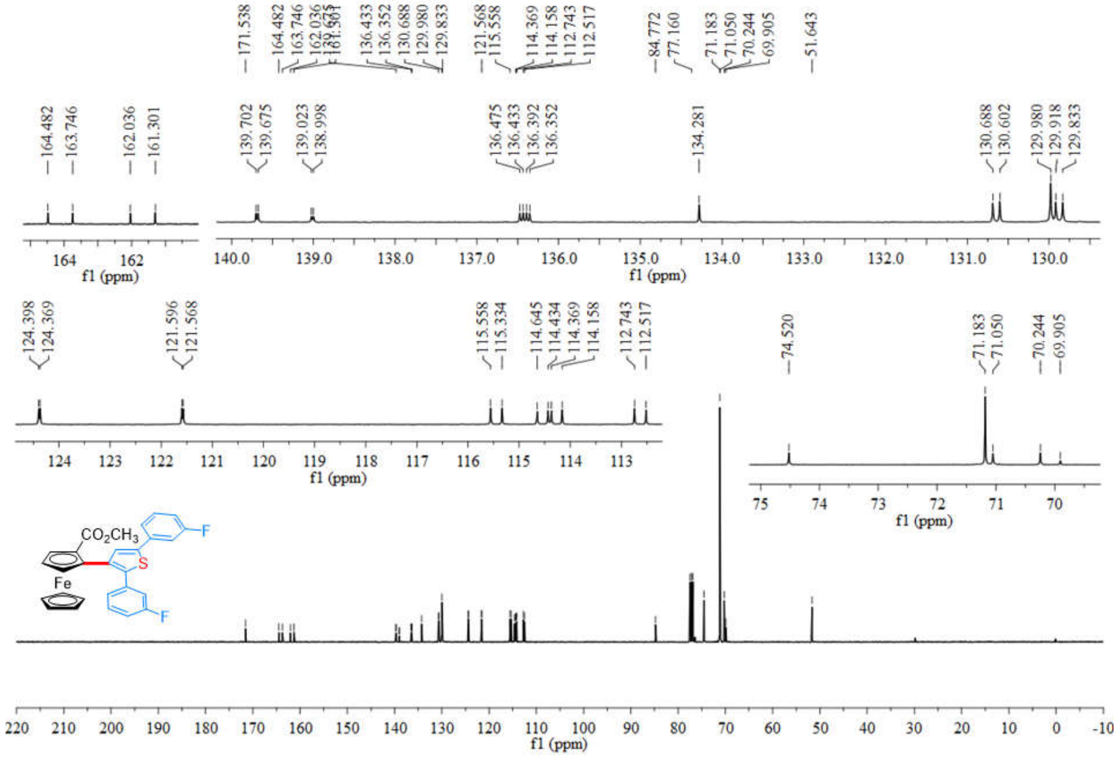

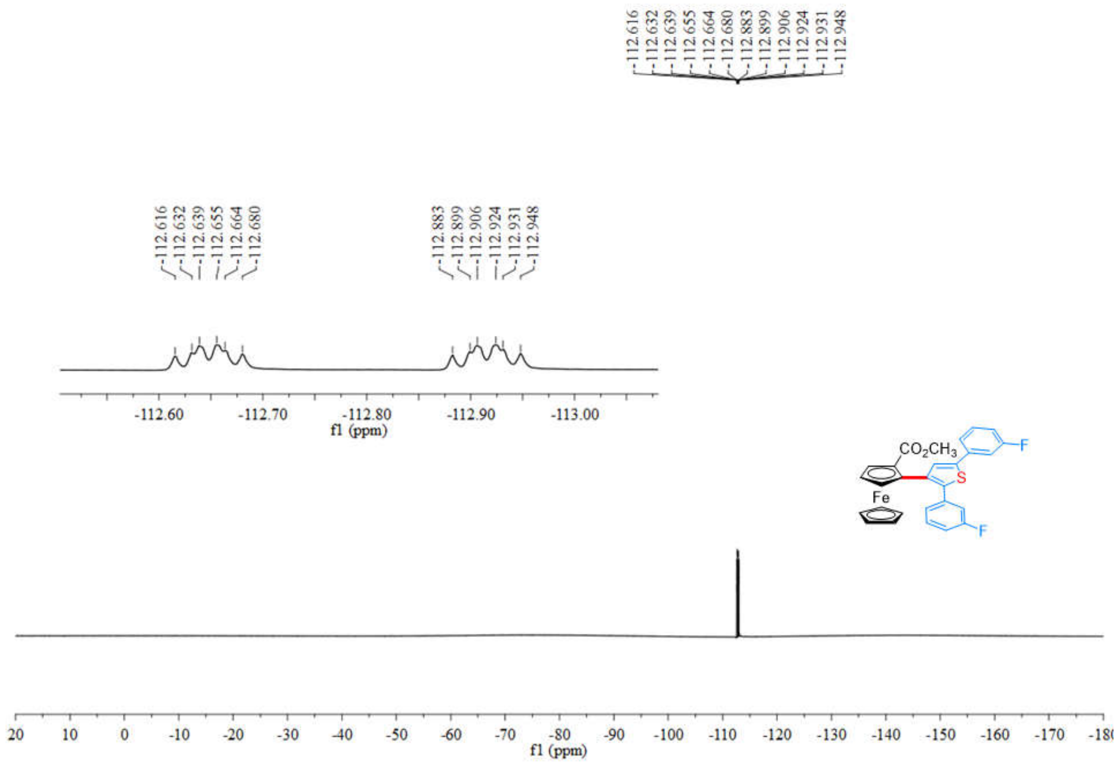
-112.616
-112.632
-112.639
-112.655
-112.664
-112.680
-112.883
-112.899
-112.906
-112.924
-112.931
-112.948
-112.60
-112.70
-112.80
-112.90
-113.00
f1 (ppm)
CO2CH3
F
S
Fe
F
20
10
0
-10
-20
-30
-40
-50
-60
-70
-80
-90
-100
-110
-120
-130
-140
-150
-160
-170
f1 (ppm)

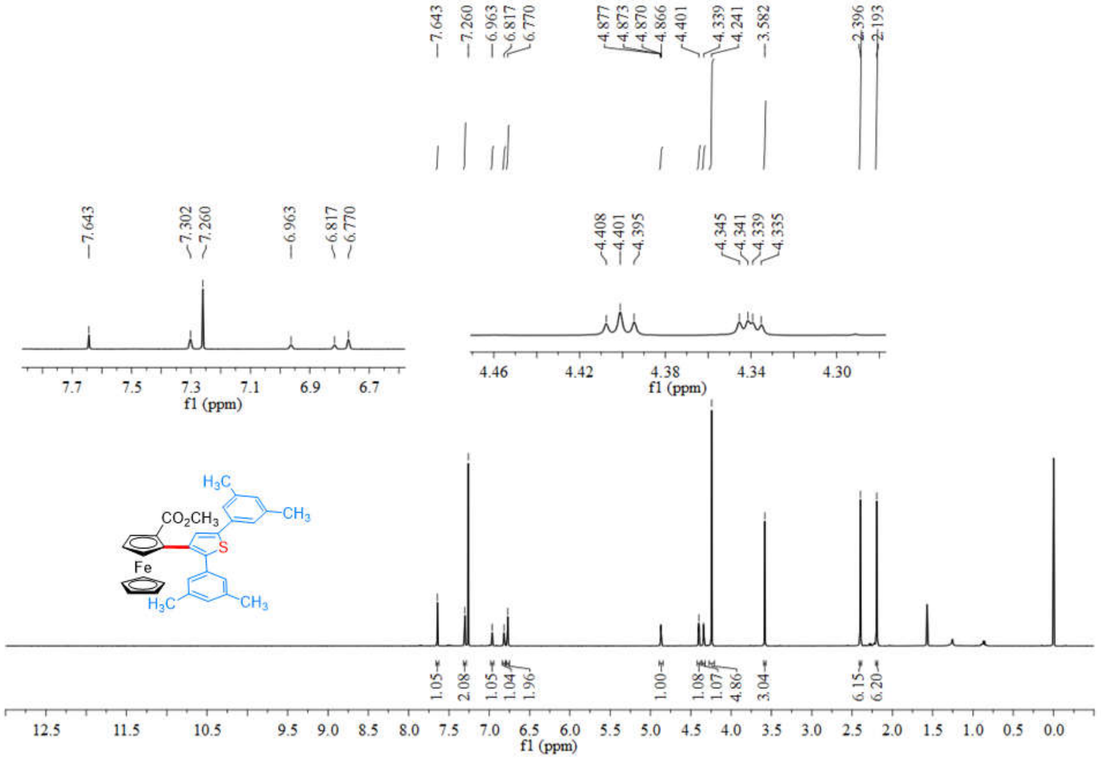
7.643 7.260 6.963 6.817 6.770 4.877 4.873 4.870 4.866 4.401 4.339 4.241 3.582 2.396 2.193
7.643 7.302 7.260 6.963 6.817 6.770
4.408 4.401 4.395 4.345 4.341 4.339 4.335
f1 (ppm)
CO2CH3
Fe
S
H3C
CH3
1.05 2.08 1.05 1.04 1.96 1.00 1.08 1.07 4.86 3.04 6.15 6.20
f1 (ppm)

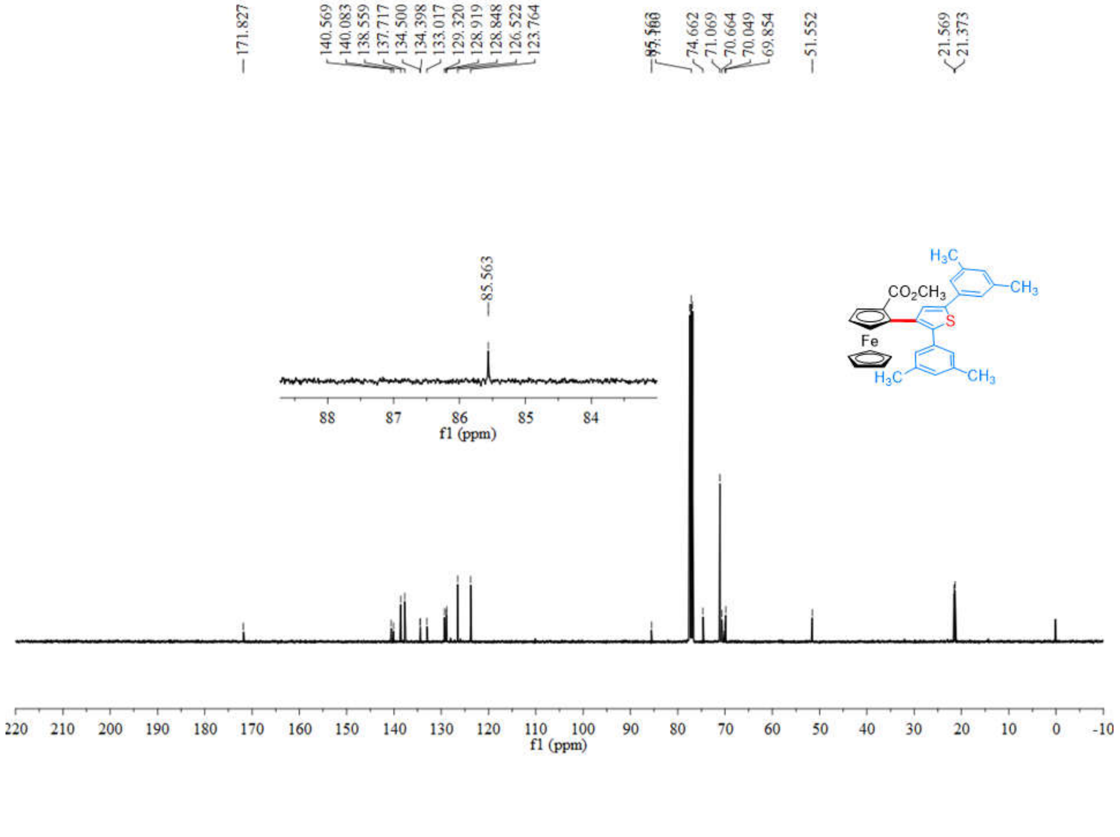
171.827 140.569 140.083 138.559 137.717 134.500 134.398 133.017 129.320 128.919 128.848 126.522 123.764 74.662 71.069 70.664 70.049 69.854 51.552 21.569 21.373
85.563
f1 (ppm)
CO2CH3
Fe
S
H3C
CH3
f1 (ppm)

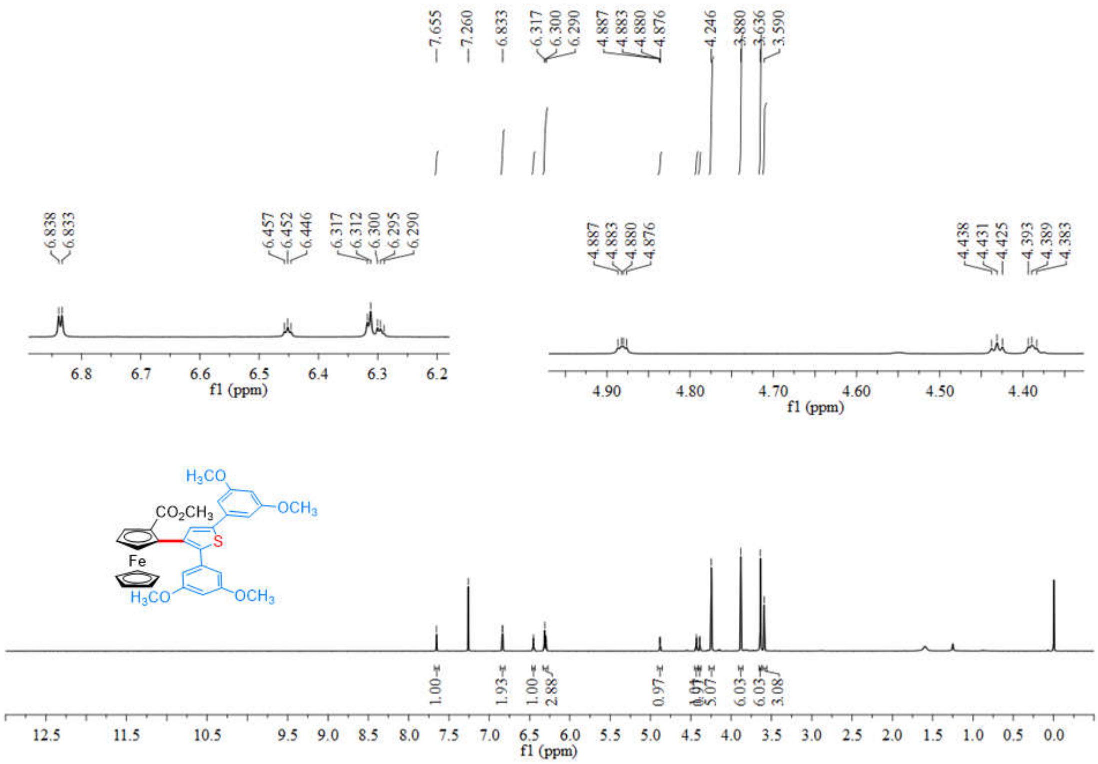

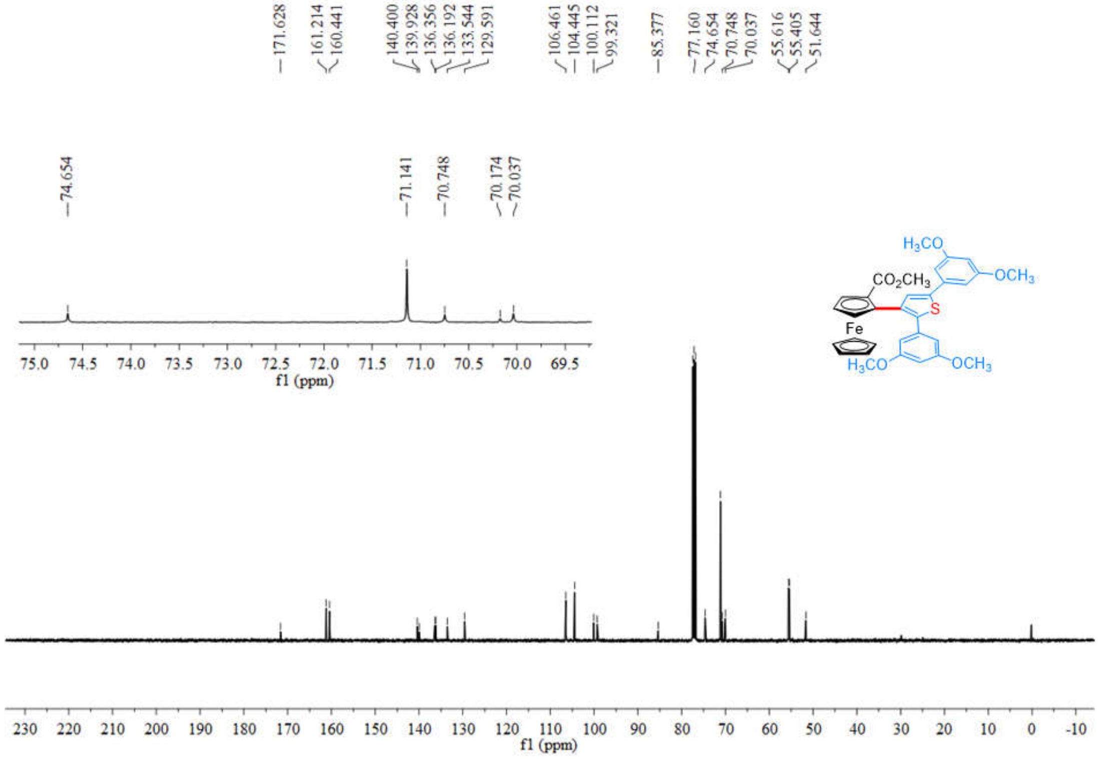

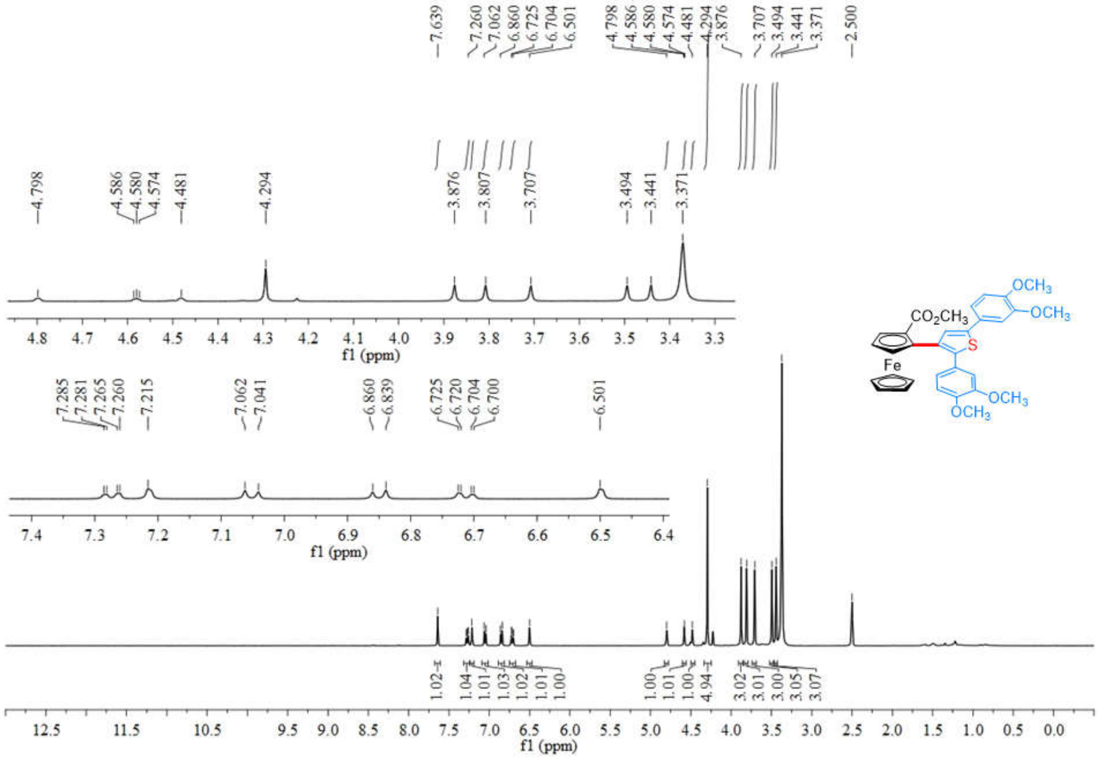
7.639
7.260
7.062
6.860
6.725
6.704
6.501
4.798
4.586
4.580
4.574
4.481
4.294
3.876
3.707
3.494
3.441
3.371
2.500
4.798
4.586
4.580
4.574
4.481
4.294
3.876
3.807
3.707
3.494
3.441
3.371
f1 (ppm)
7.285
7.281
7.265
7.260
7.215
7.062
7.041
6.860
6.839
6.725
6.720
6.704
6.700
6.501
f1 (ppm)
CO2CH3
Fe
S
OCH3
OCH3
OCH3
OCH3
1.02
1.04
1.01
1.01
1.03
1.02
1.01
1.00
1.00
1.01
1.00
4.94
3.02
3.01
3.00
3.05
3.07
f1 (ppm)

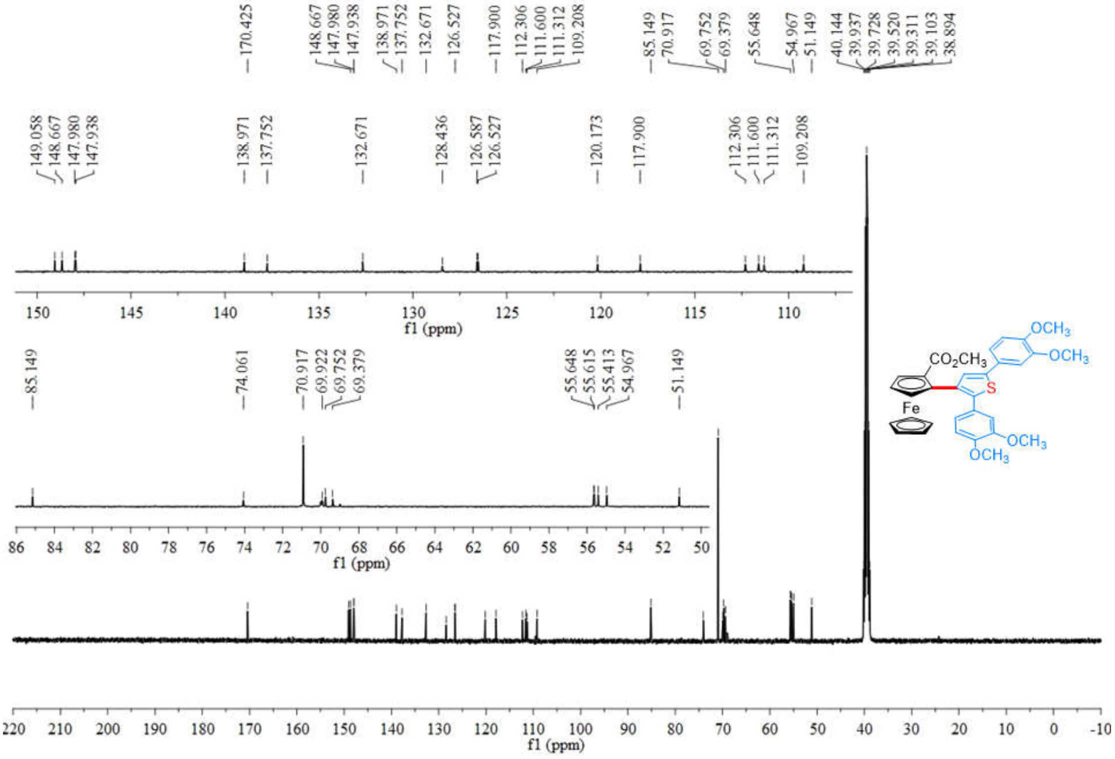
170.425
148.667
147.980
147.938
138.971
137.752
132.671
126.527
117.900
112.306
111.600
111.312
109.208
85.149
70.917
69.752
69.379
55.648
54.967
51.149
40.144
39.937
39.728
39.520
39.311
39.103
38.894
149.058
148.667
147.980
147.938
138.971
137.752
132.671
128.436
126.587
126.527
120.173
117.900
112.306
111.600
111.312
109.208
f1 (ppm)
85.149
74.061
70.917
69.922
69.752
69.379
55.648
55.615
55.413
54.967
51.149
f1 (ppm)
CO2CH3
Fe
S
OCH3
OCH3
OCH3
OCH3
f1 (ppm)

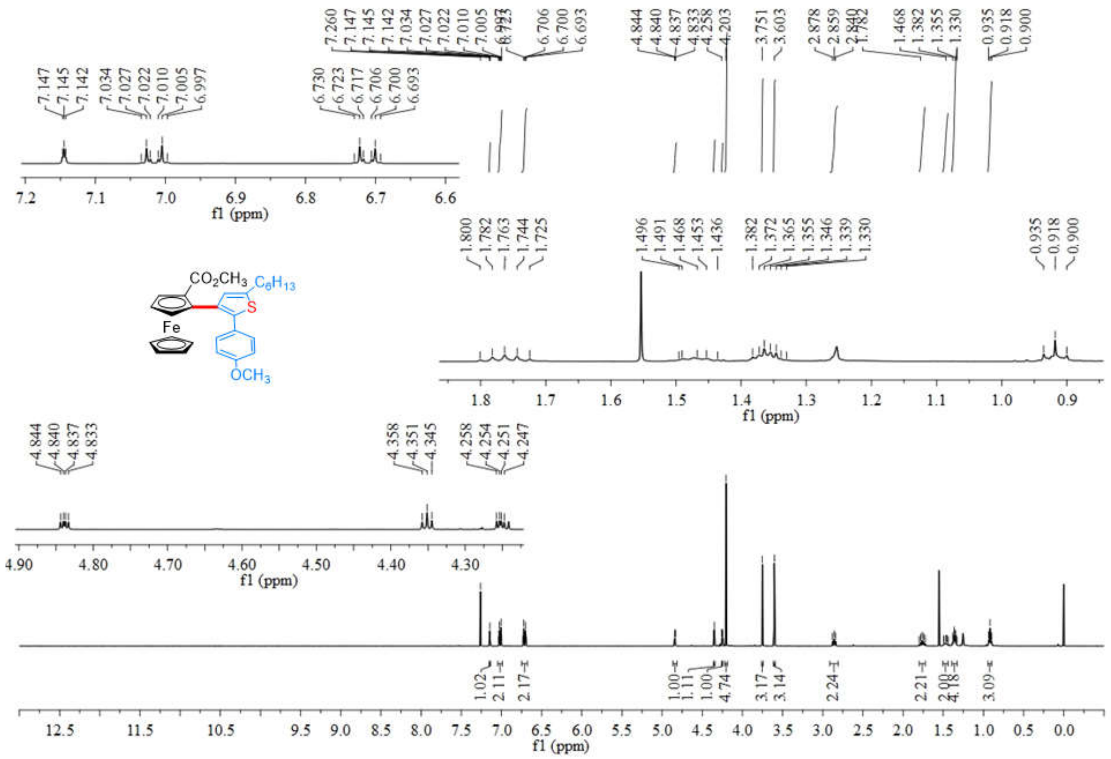

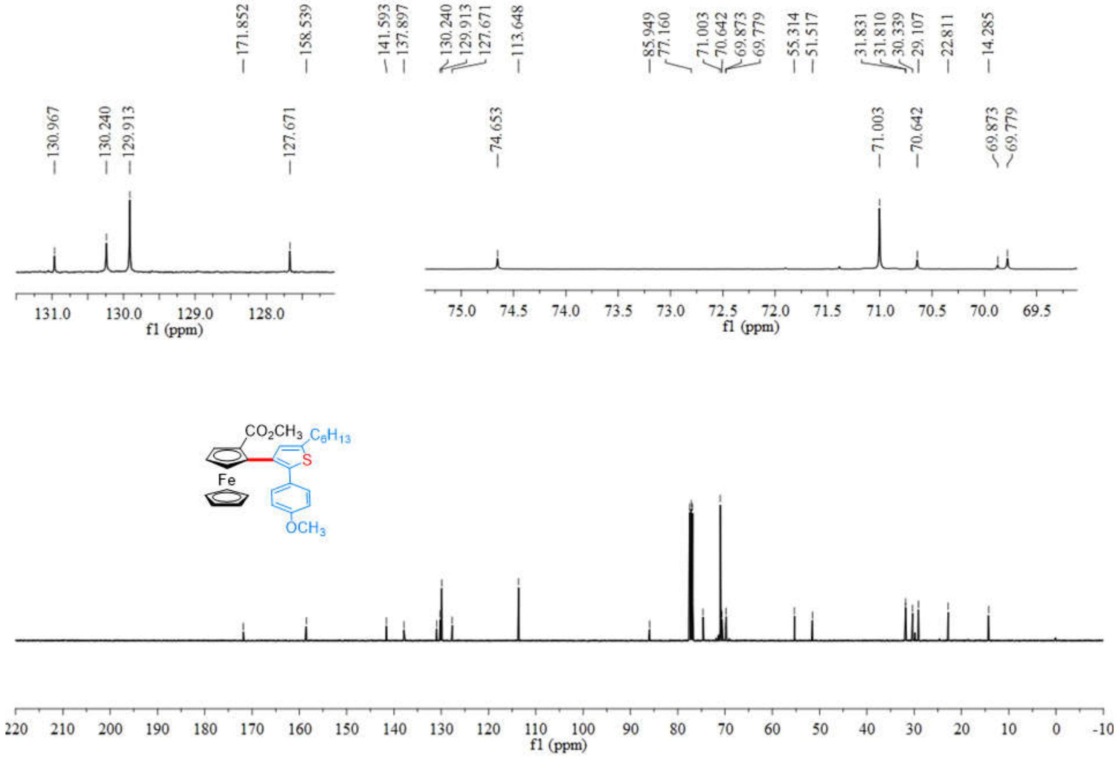

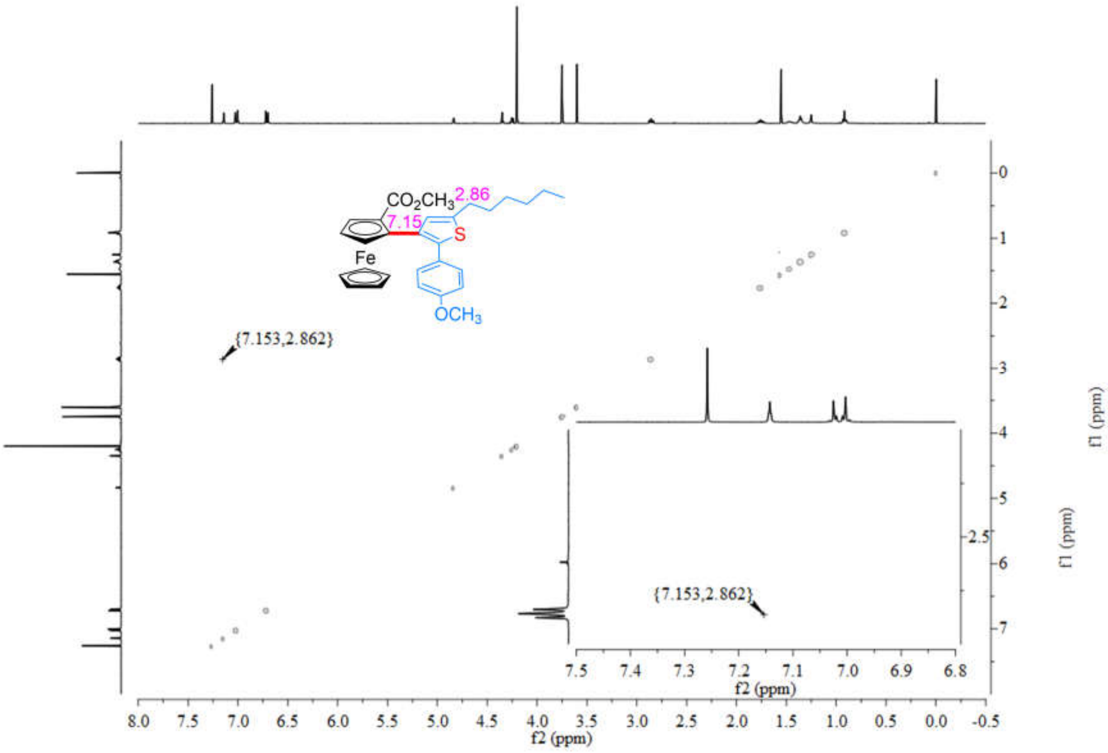

CO2CH3
2.86
7.15
Fe
S
OCH3
{7.153,2.862}
f2 (ppm)
f1 (ppm)

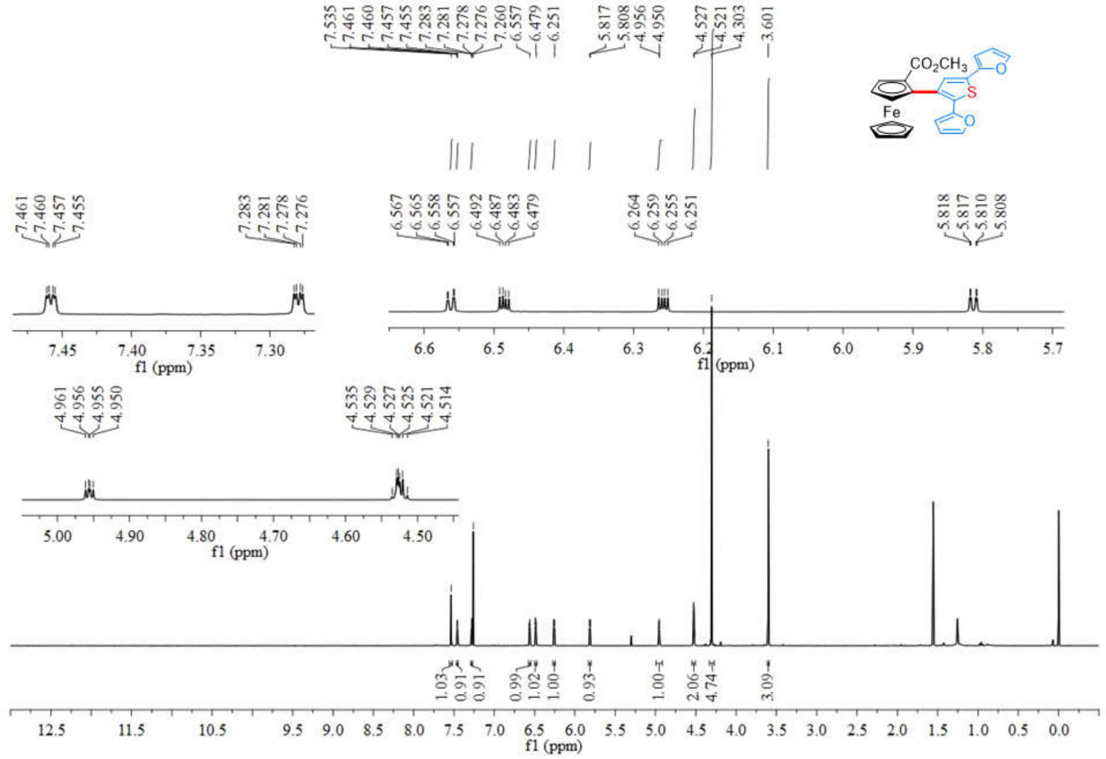

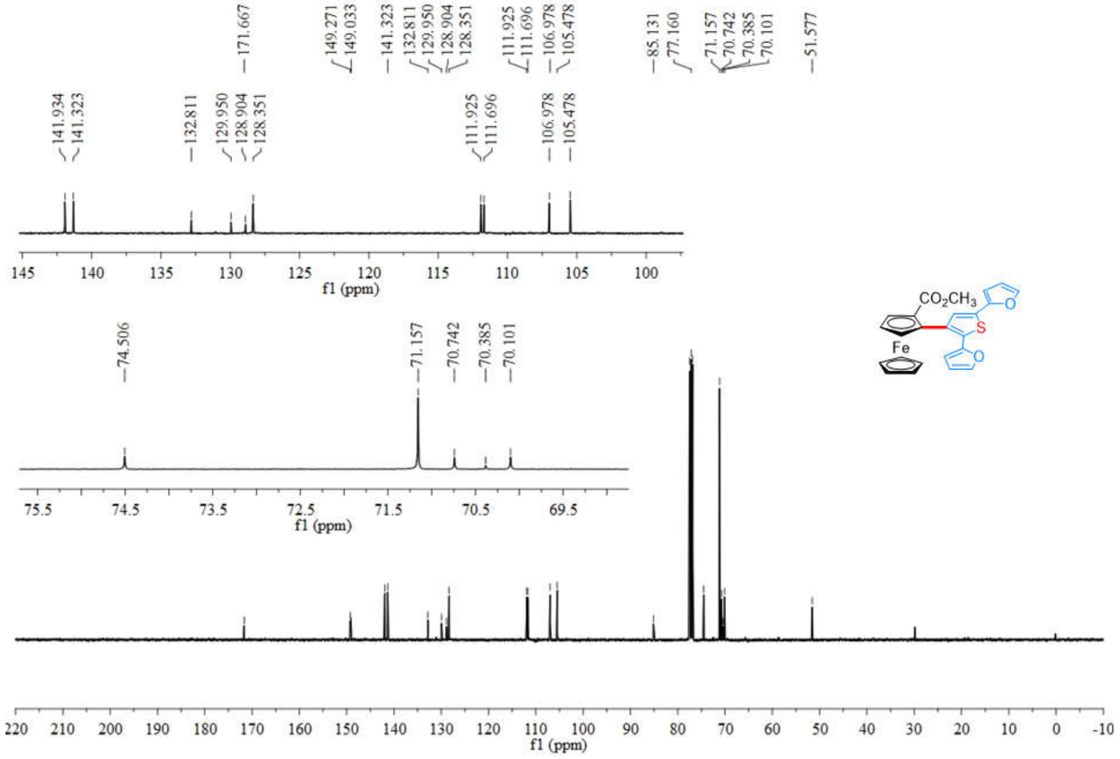

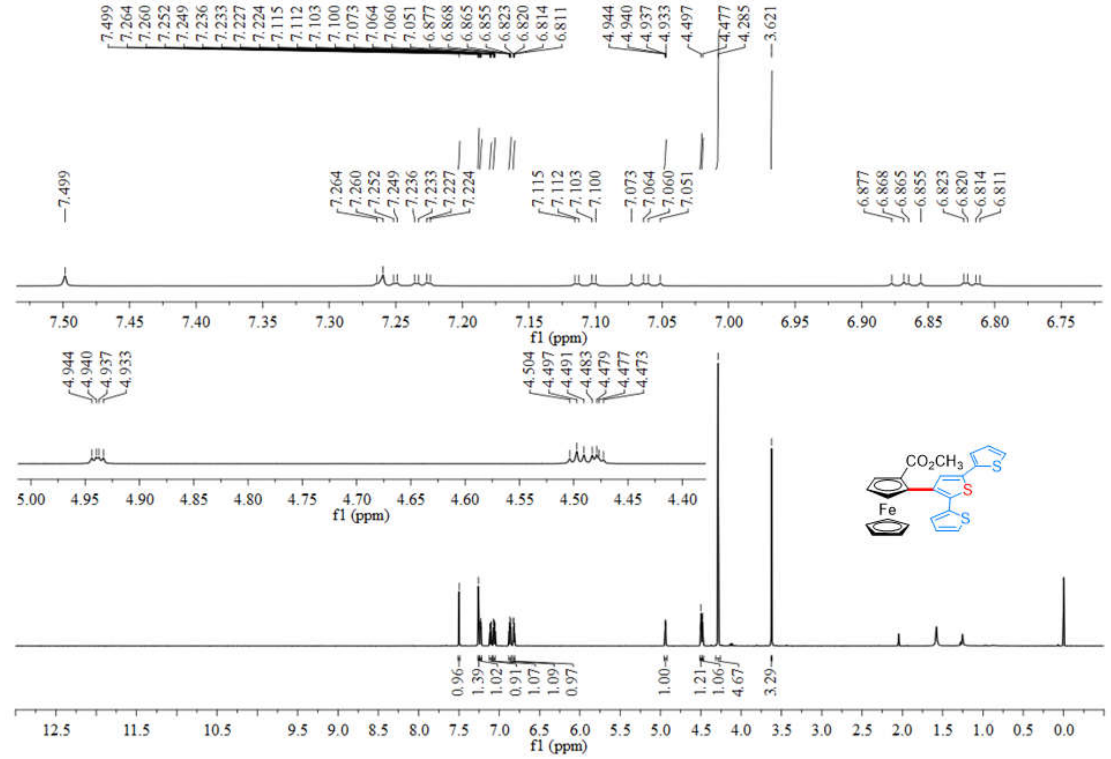

f1 (ppm)
CO2CH3
Fe
S


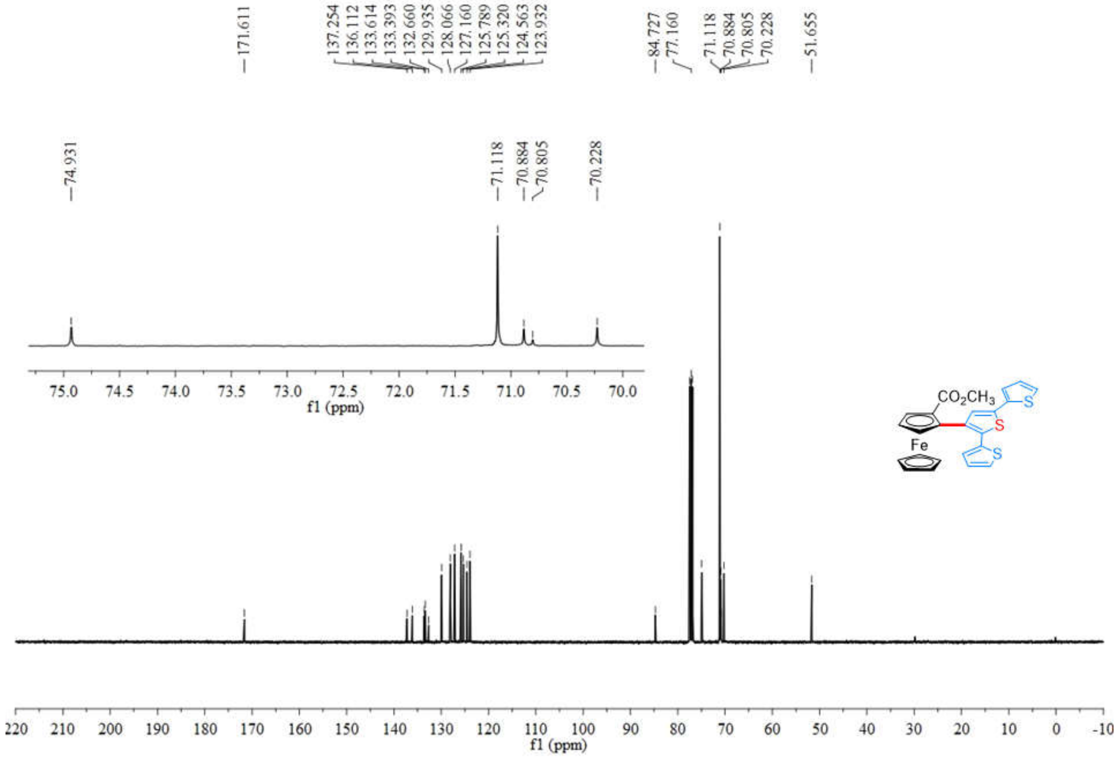

f1 (ppm)
CO2CH3
Fe
S

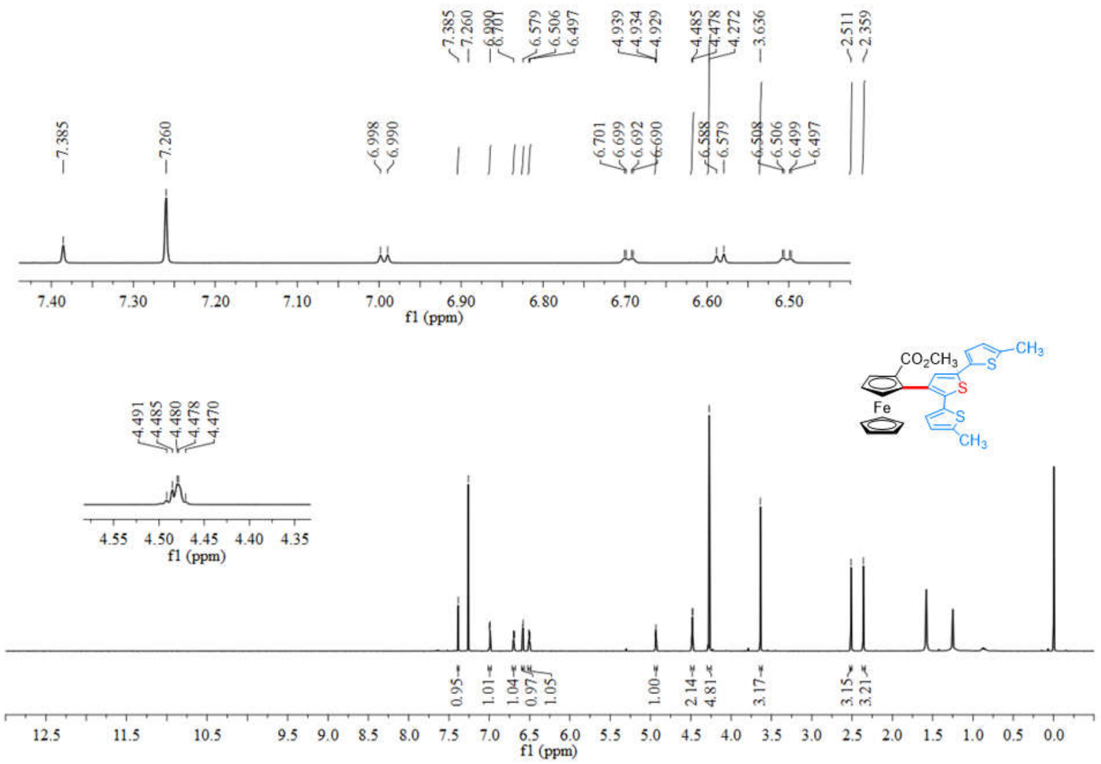

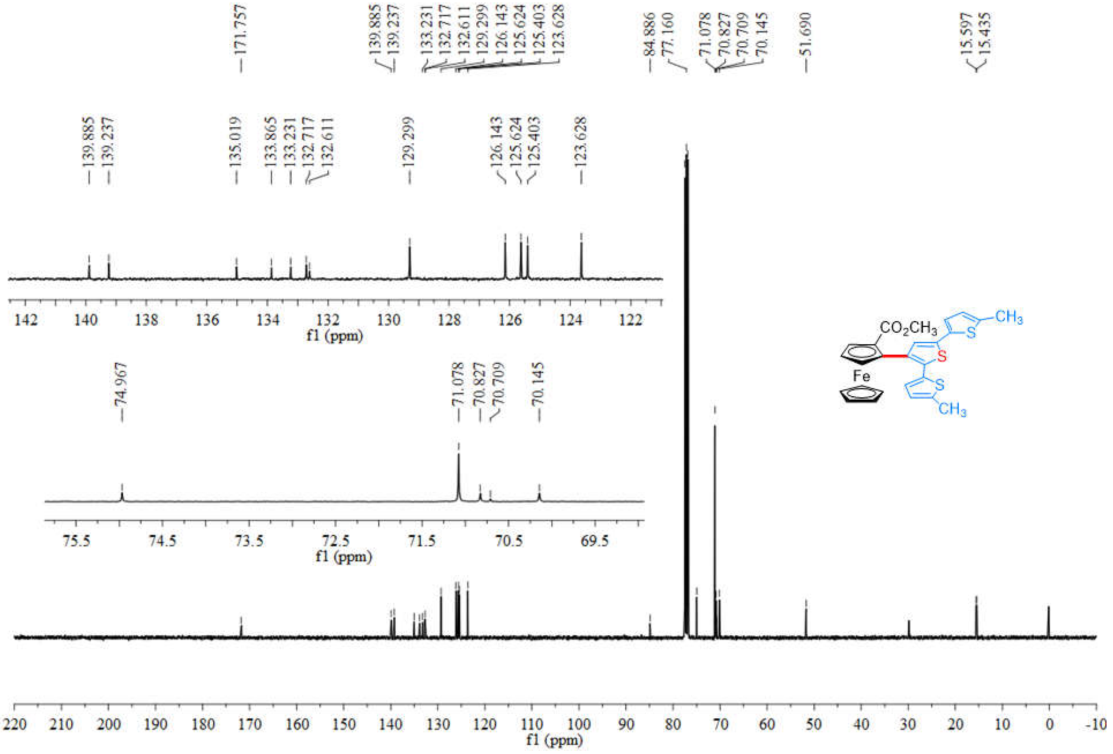

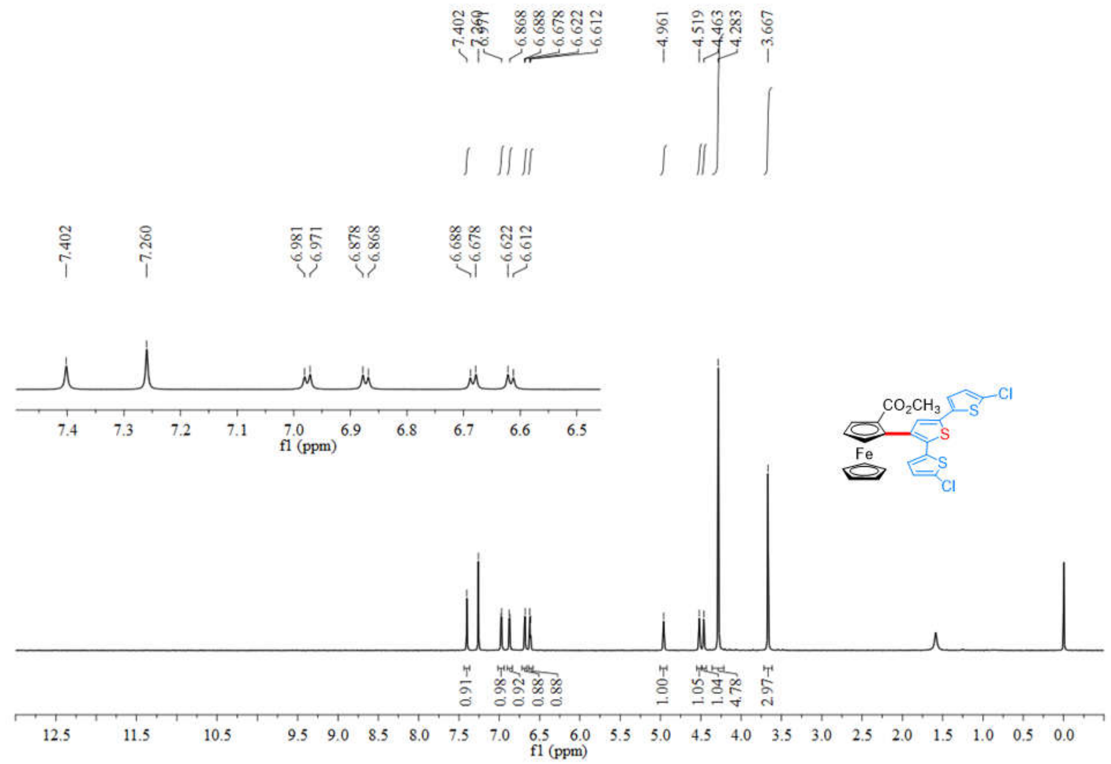
7.402
7.260
6.971
6.868
6.688
6.678
6.622
6.612
4.961
4.519
4.463
4.283
3.667
7.402
7.260
6.981
6.971
6.878
6.868
6.688
6.678
6.622
6.612
f1 (ppm)
CO2CH3
Cl
S
Fe
S
Cl
0.91
0.98
0.92
0.88
0.88
1.00
1.05
1.04
4.78
2.97
f1 (ppm)

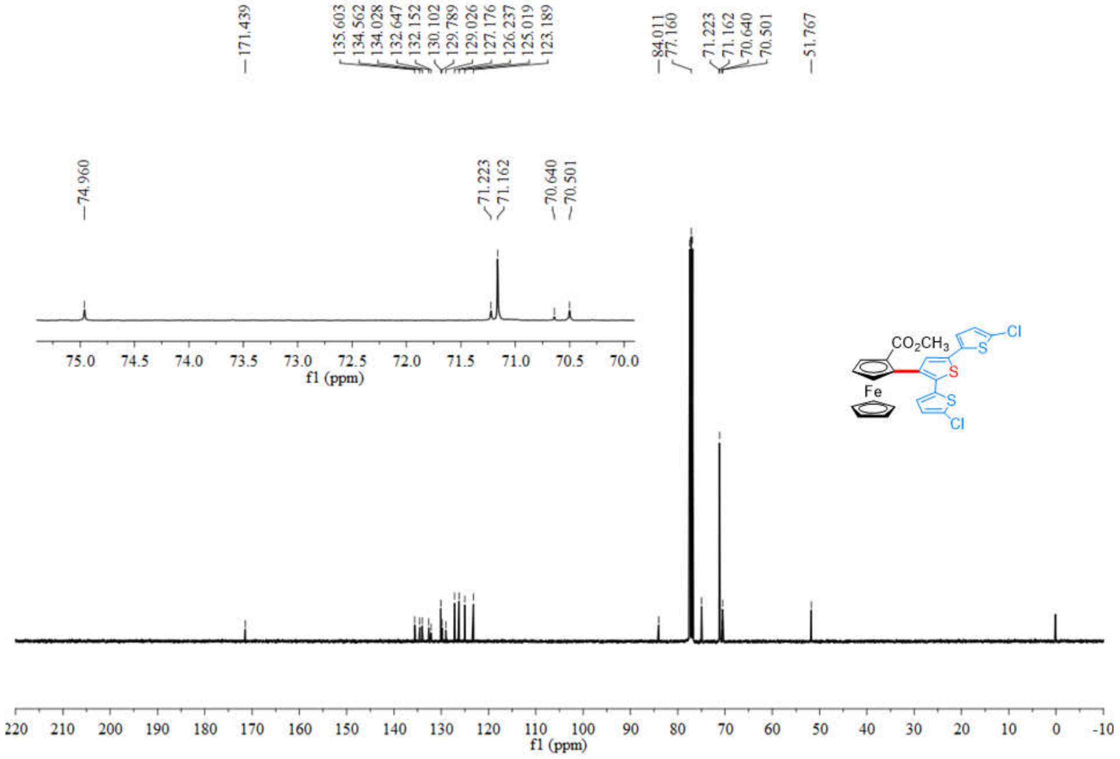
171.439
135.603
134.562
134.028
132.647
132.152
130.102
129.789
129.026
127.176
126.237
125.019
123.189
84.011
77.160
71.223
71.162
70.640
70.501
51.767
74.960
71.223
71.162
70.640
70.501
f1 (ppm)
CO2CH3
Cl
S
Fe
S
Cl
f1 (ppm)

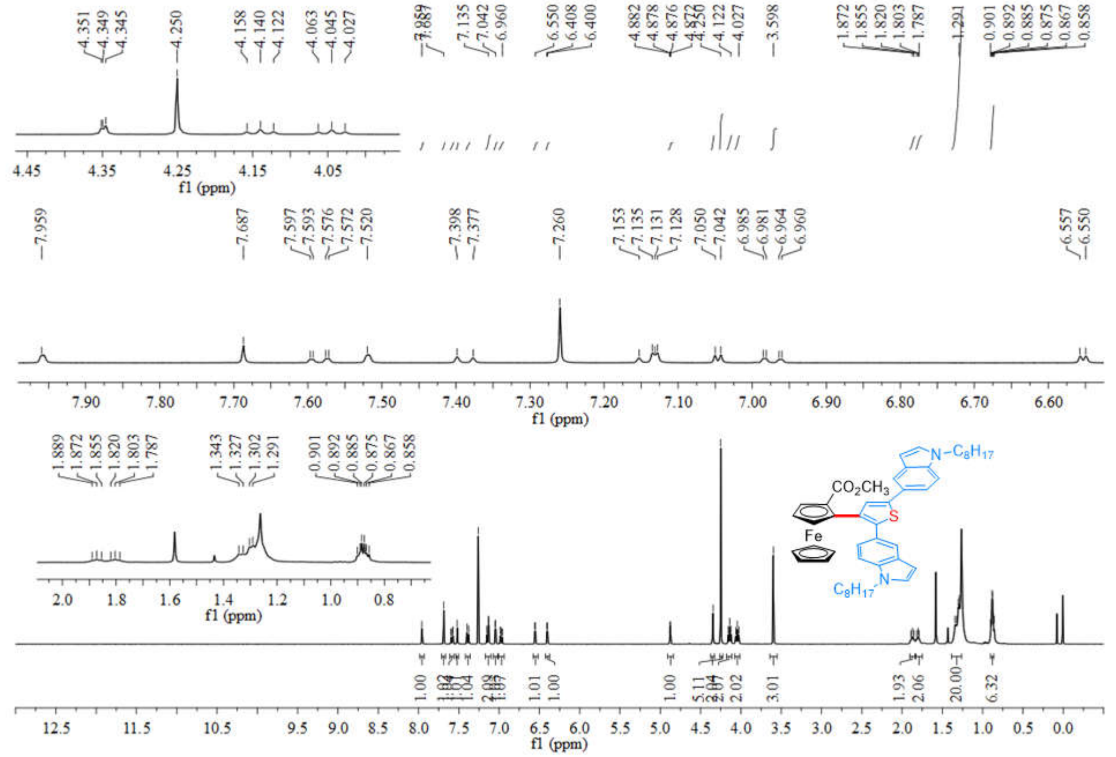

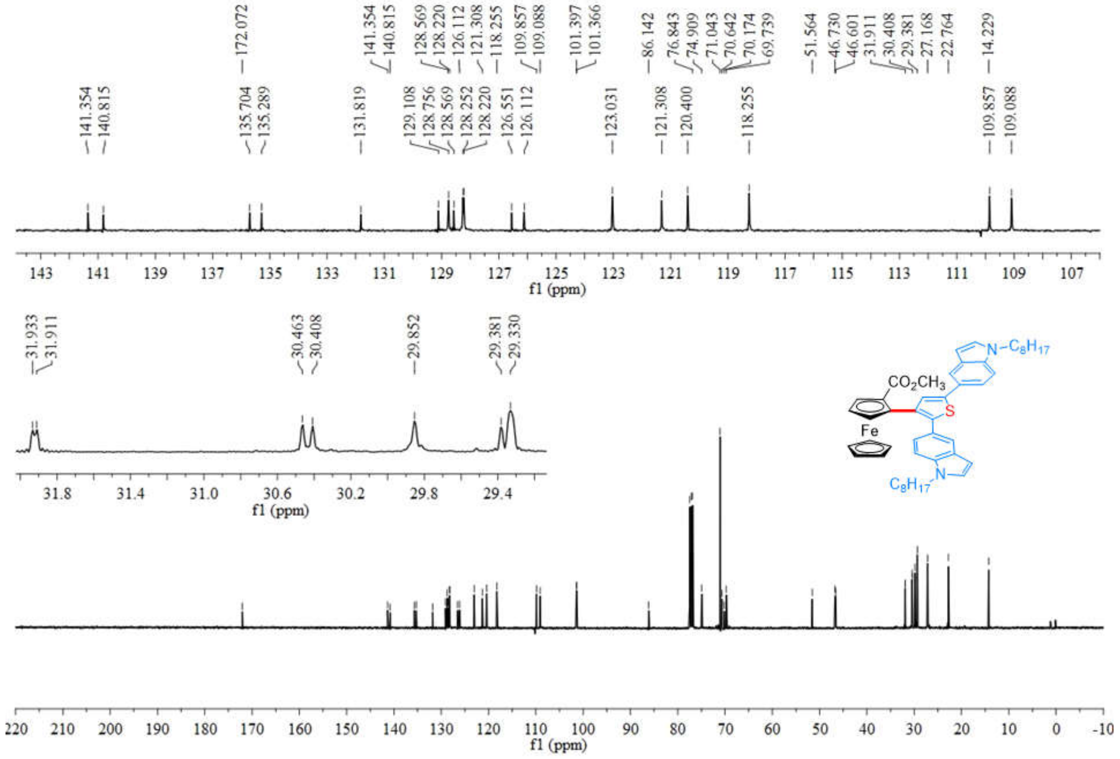

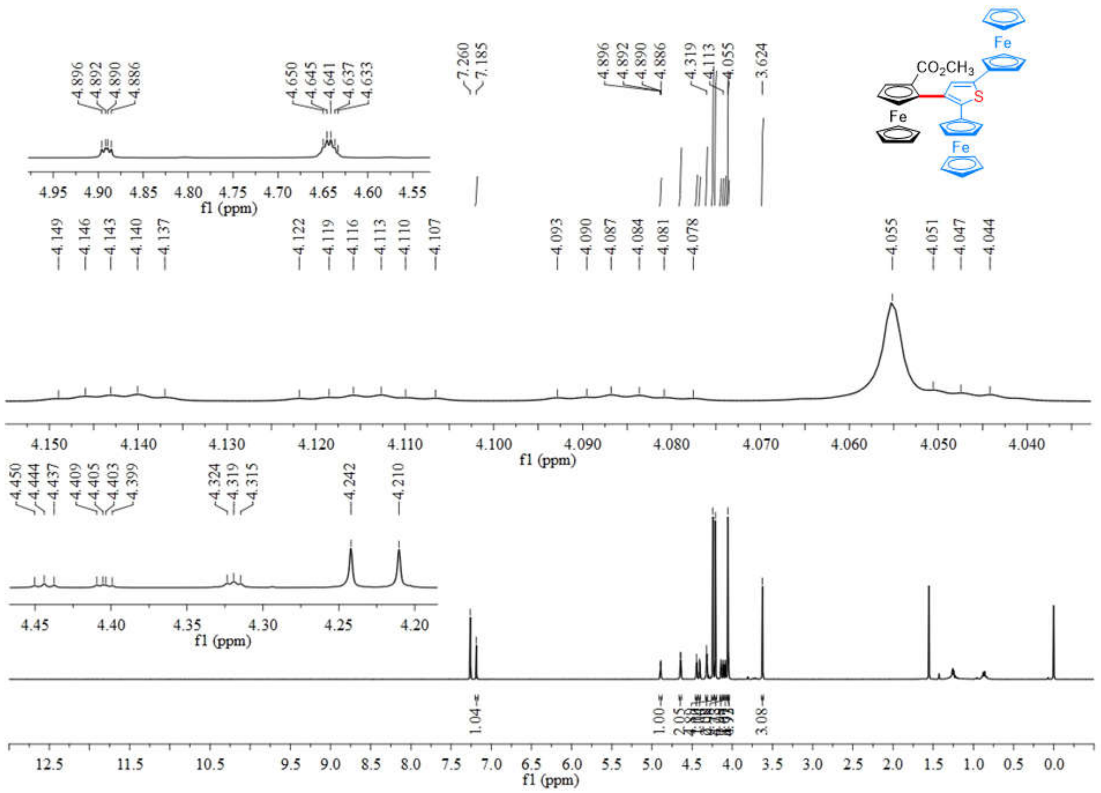

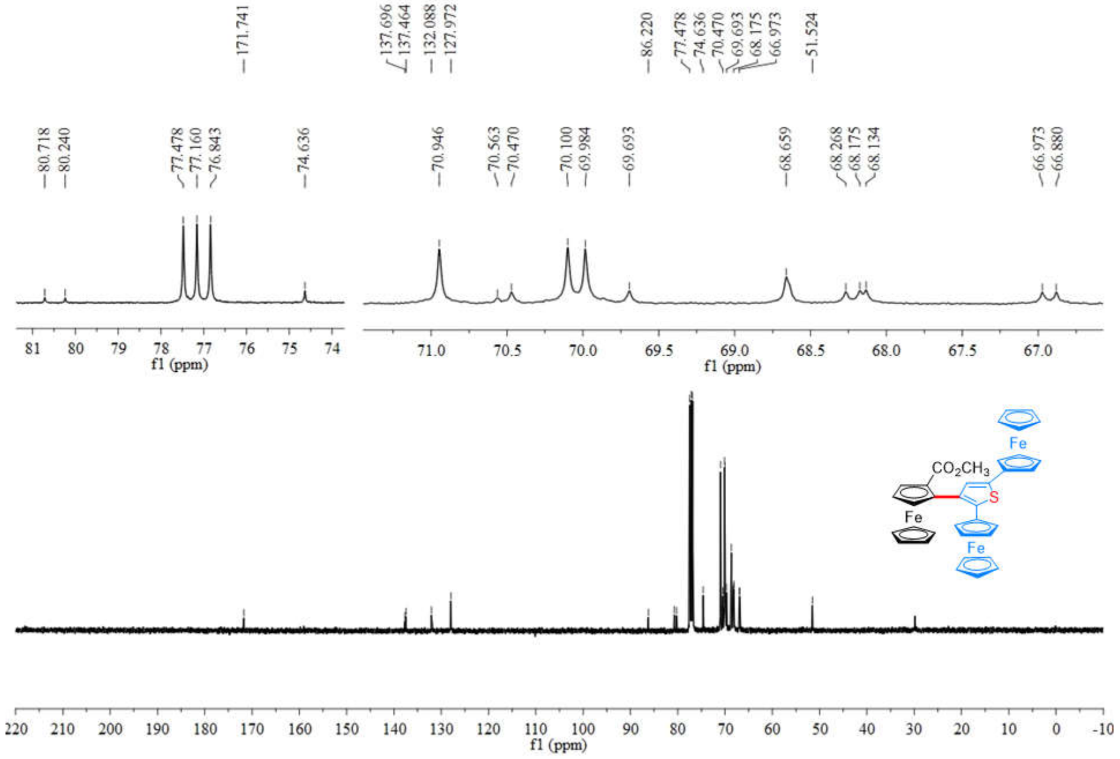

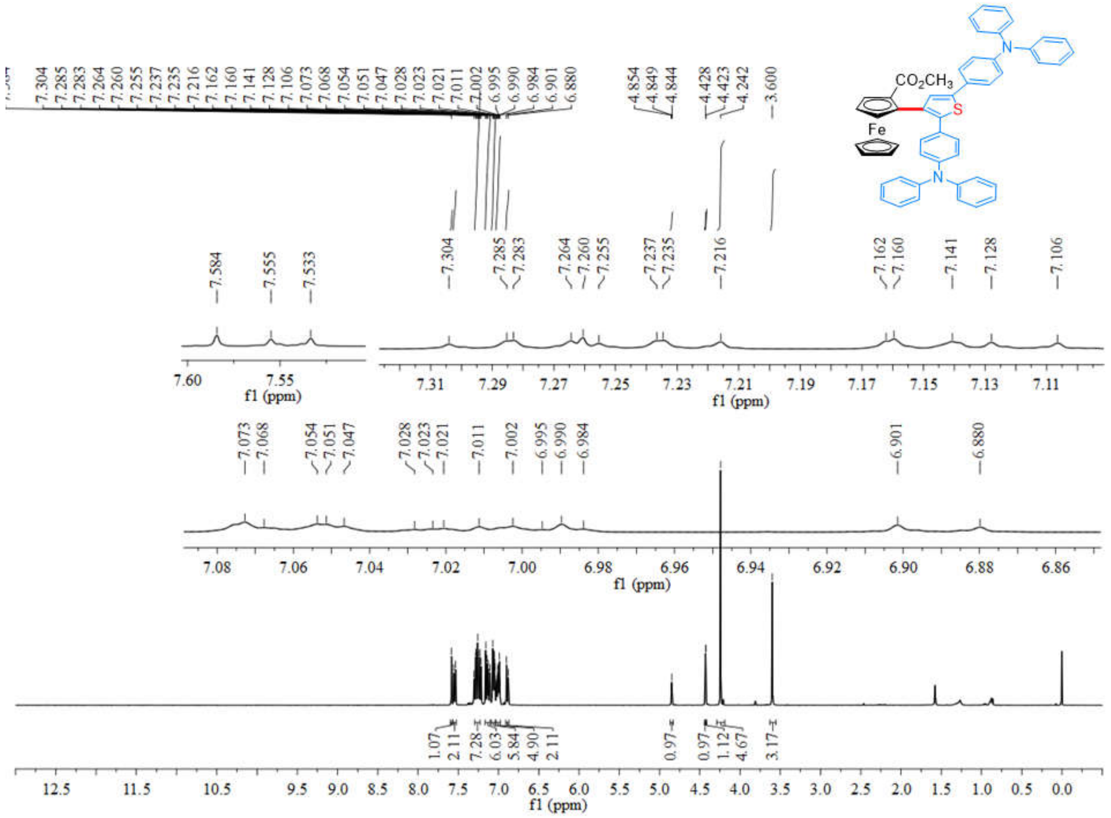

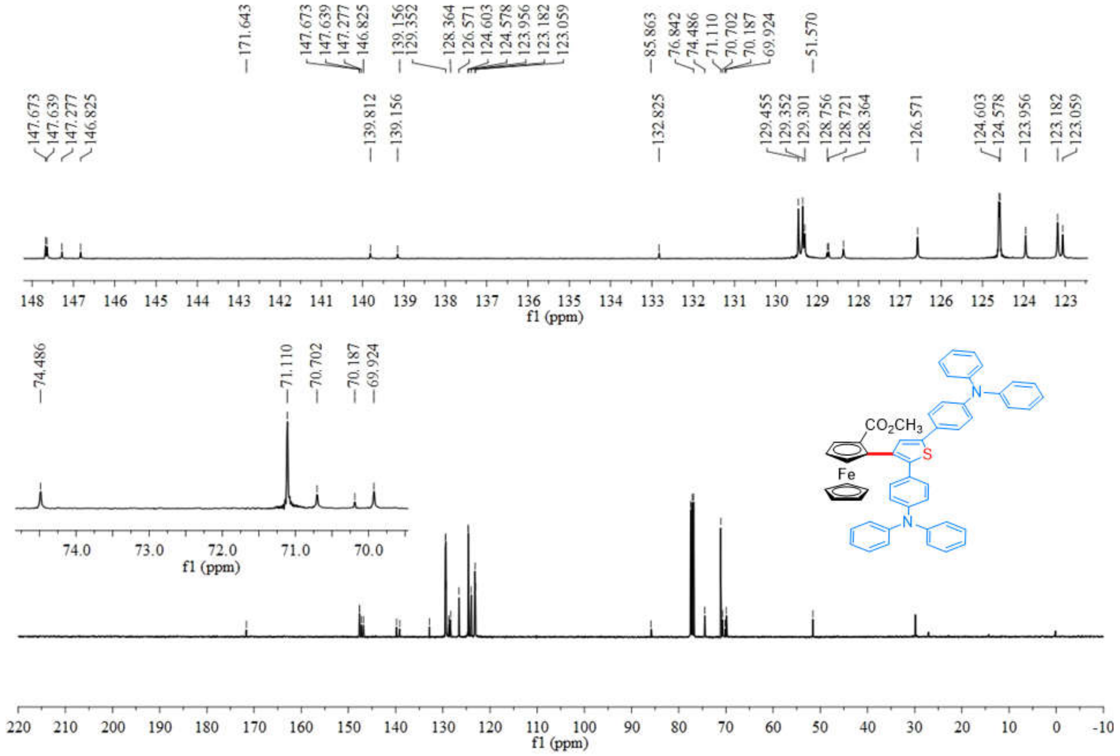

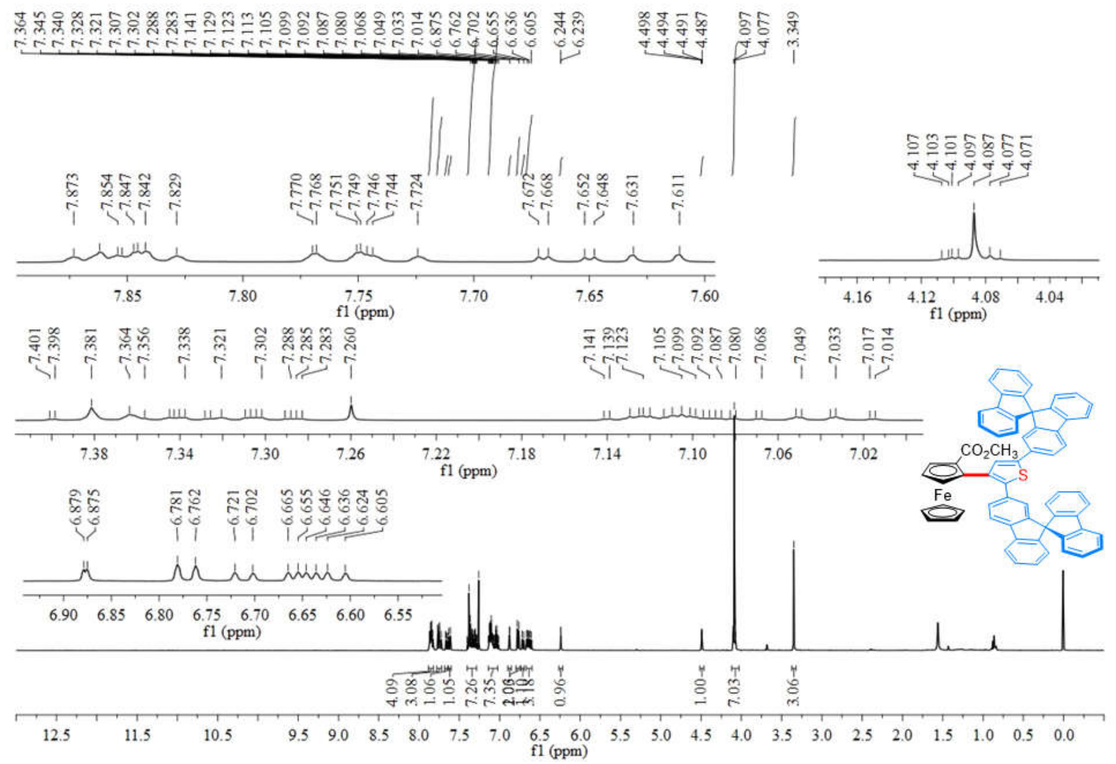

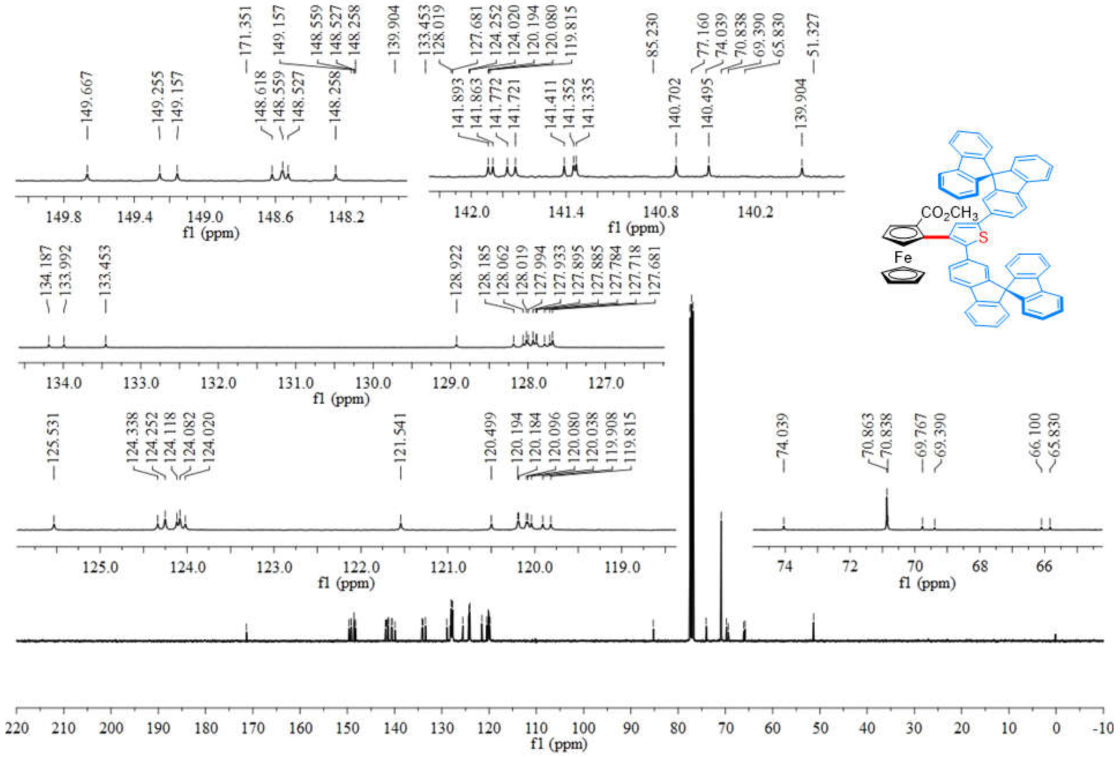

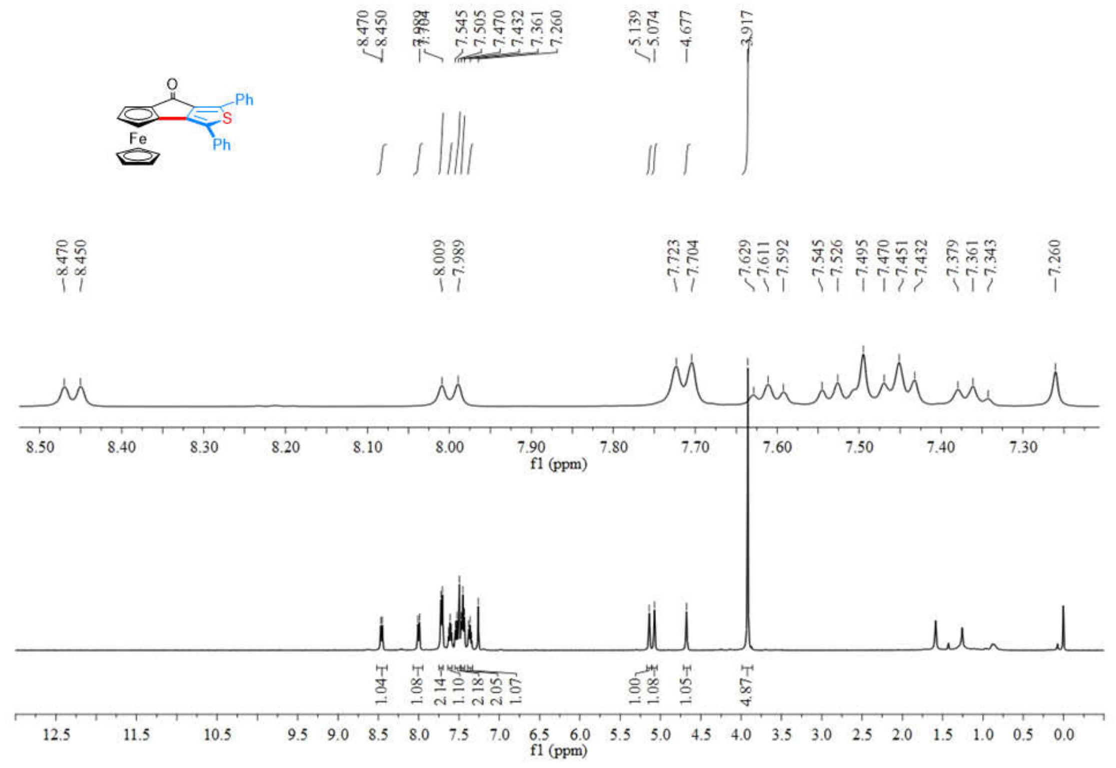
O
Ph
S
Fe
Ph
8.470
8.450
7.989
7.704
7.545
7.505
7.470
7.432
7.361
7.260
5.139
5.074
4.677
3.917
8.470
8.450
8.009
7.989
7.723
7.704
7.629
7.611
7.592
7.545
7.526
7.495
7.470
7.451
7.432
7.379
7.361
7.343
7.260
f1 (ppm)
1.04
1.08
2.14
1.10
2.18
2.05
1.07
1.00
1.08
1.05
4.87
f1 (ppm)

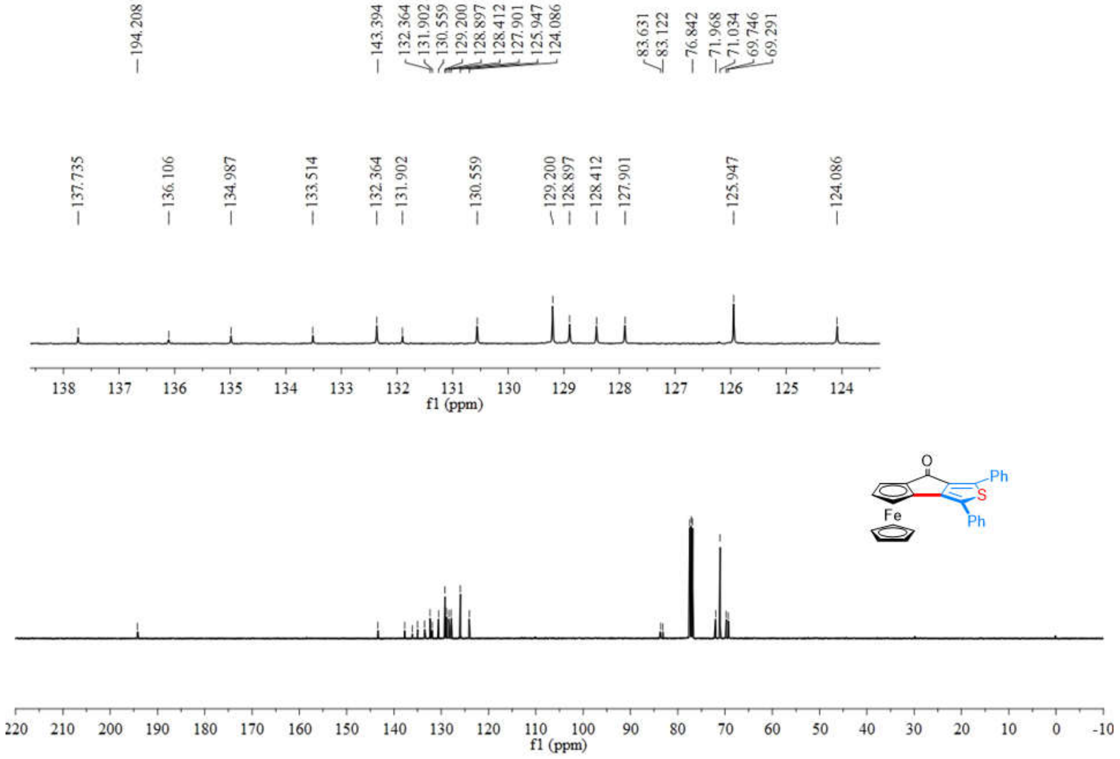
194.208
143.394
132.364
131.902
130.559
129.200
128.897
128.412
127.901
125.947
124.086
83.631
83.122
76.842
71.968
71.034
69.746
69.291
137.735
136.106
134.987
133.514
132.364
131.902
130.559
129.200
128.897
128.412
127.901
125.947
124.086
f1 (ppm)
O
Ph
S
Fe
Ph
f1 (ppm)

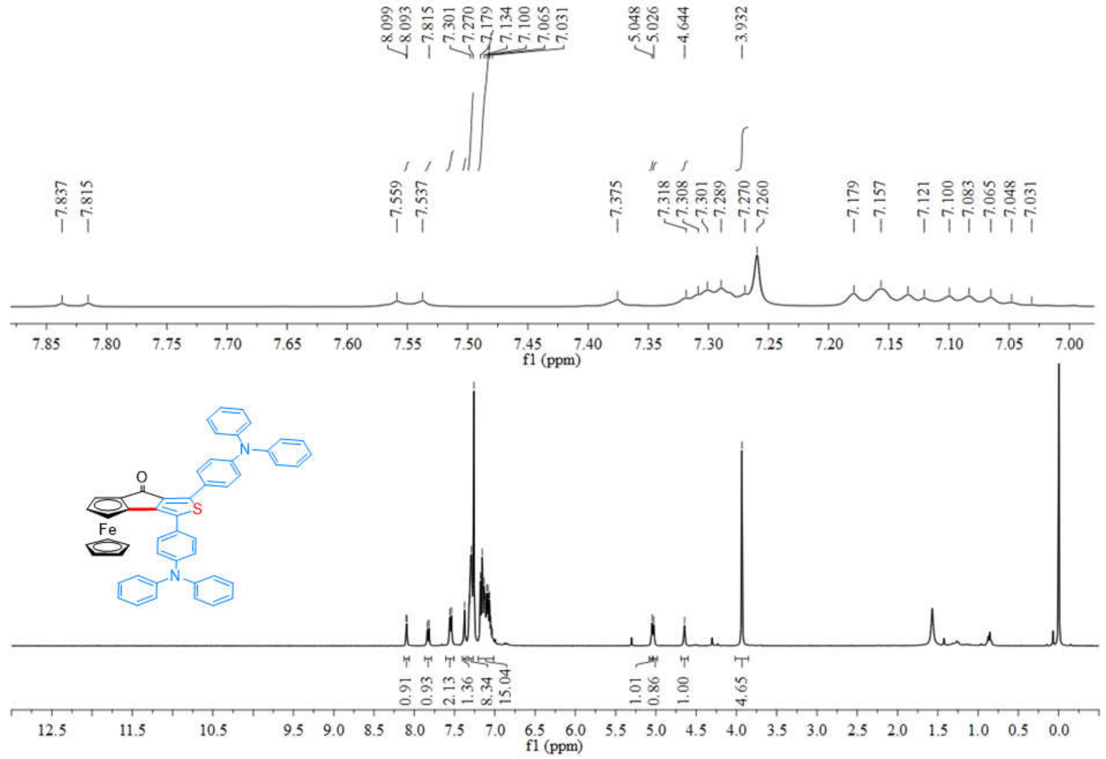

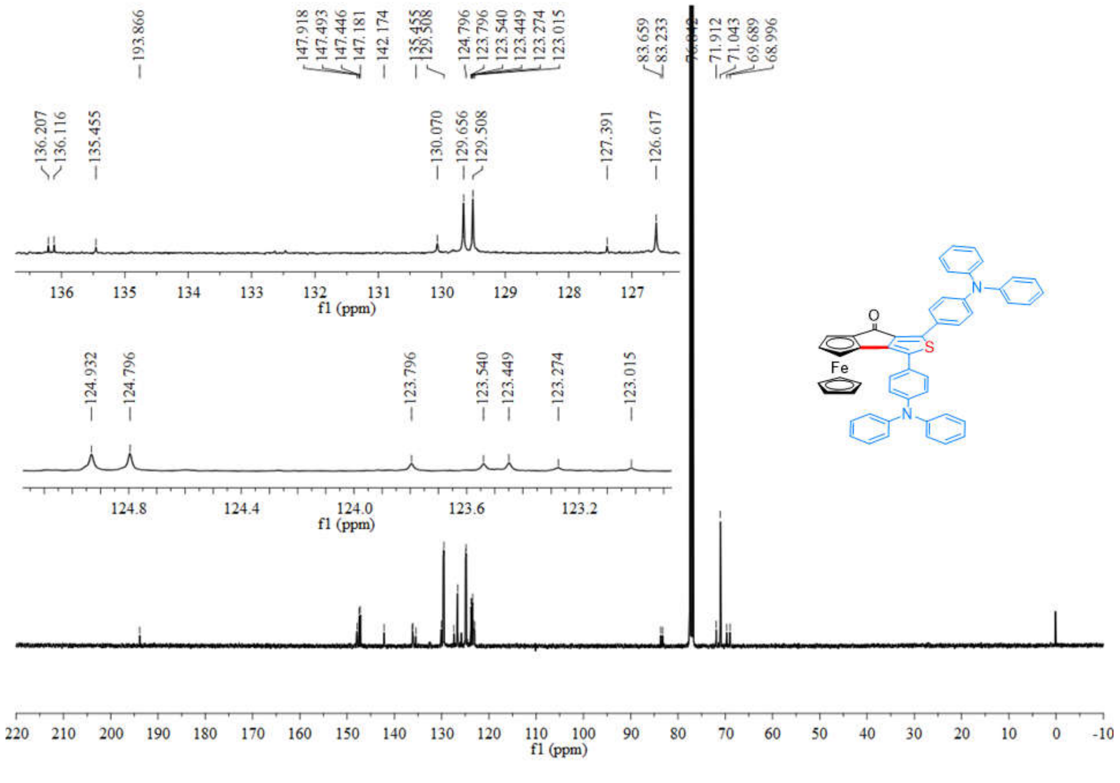

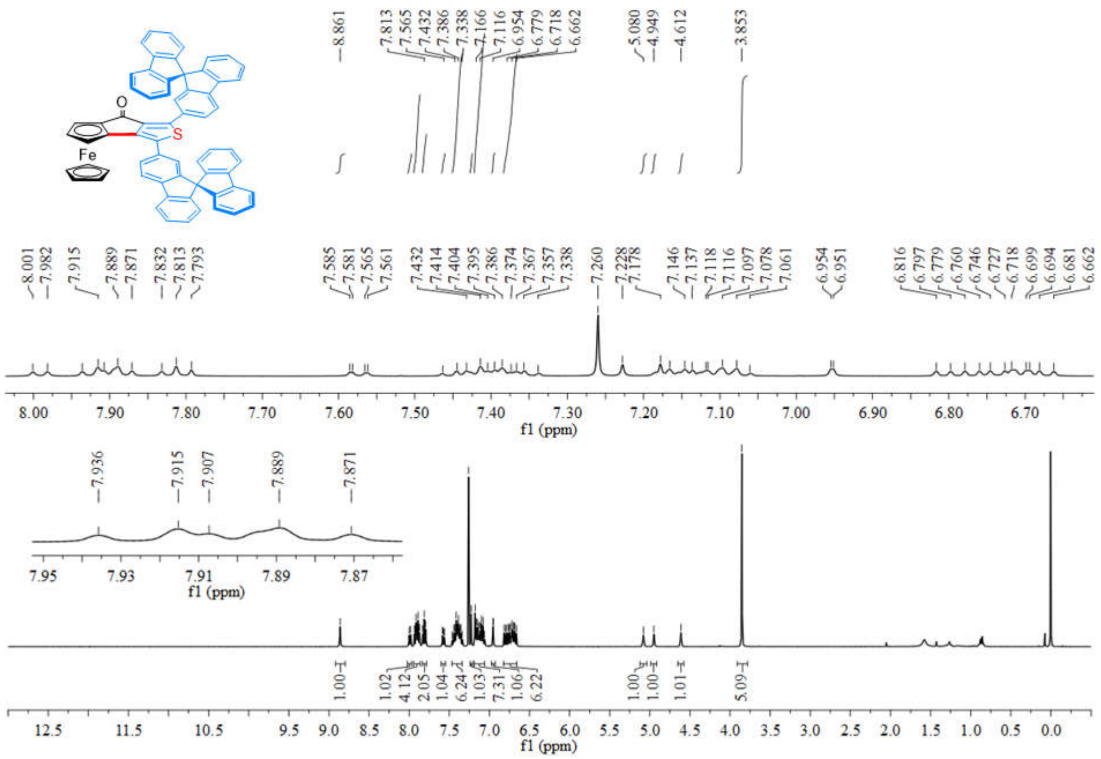
O
Fe
S
8.861
7.813 7.565 7.432 7.386 7.338 7.166 7.116 6.954 6.779 6.718 6.662
5.080 4.949
4.612
3.853
8.001 7.982 7.915 7.889 7.871 7.832 7.813 7.793
7.585 7.581 7.565 7.561
7.432 7.414 7.404 7.395 7.386 7.374 7.367 7.357 7.338
7.260 7.228 7.178
7.146 7.137 7.118 7.116 7.097 7.078 7.061
6.954 6.951
6.816 6.797 6.779 6.760 6.746 6.727 6.718 6.699 6.694 6.681 6.662
f1 (ppm)
7.936 7.915 7.907 7.889 7.871
f1 (ppm)
1.00 1.02 4.12 2.05 1.04 6.24 1.03 7.31 1.06 6.22 1.00 1.00 1.01 5.09
f1 (ppm)

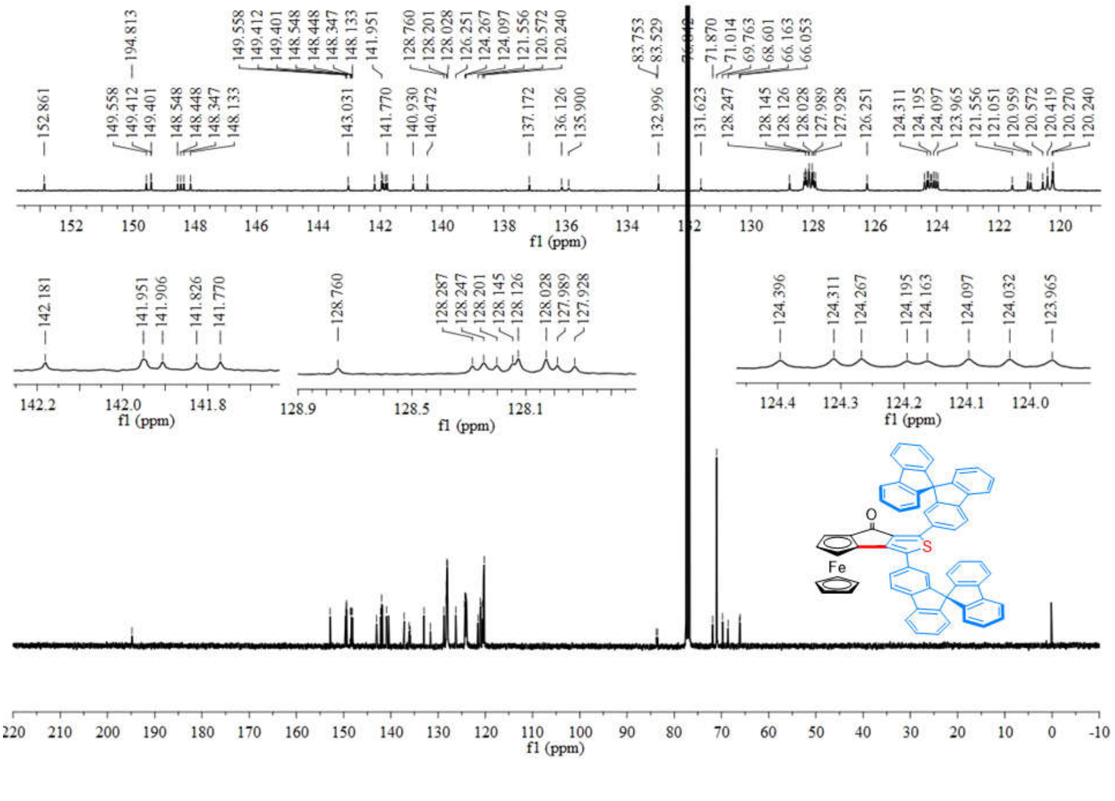
194.813
149.558 149.412 149.401 148.548 148.448 148.347 148.133 141.951 128.760 128.201 128.028 126.251 124.267 124.097 121.556 120.572 120.240
83.753 83.529
[illegible]
71.870 71.014 69.763 68.601 66.163 66.053
152.861
149.558 149.412 149.401
148.548 148.448 148.347 148.133
143.031 141.770 140.930 140.472
137.172 136.126 135.900
132.996 131.623
128.247 128.145 128.126 128.028 127.989 127.928
126.251
124.311 124.195 124.097 123.965
121.556 121.051 120.959 120.572 120.419 120.270 120.240
f1 (ppm)
142.181 141.951 141.906 141.826 141.770
f1 (ppm)
128.760 128.287 128.247 128.201 128.145 128.126 128.028 127.989 127.928
f1 (ppm)
124.396 124.311 124.267 124.195 124.163 124.097 124.032 123.965
f1 (ppm)
O
Fe
S
f1 (ppm)

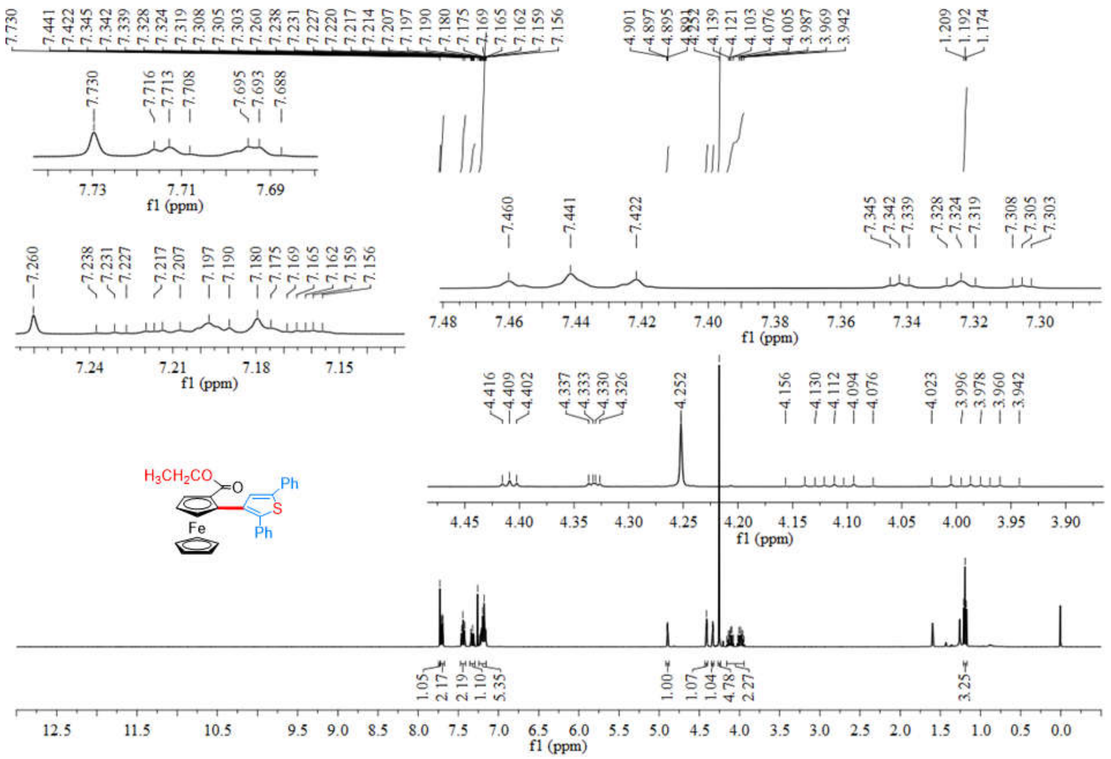

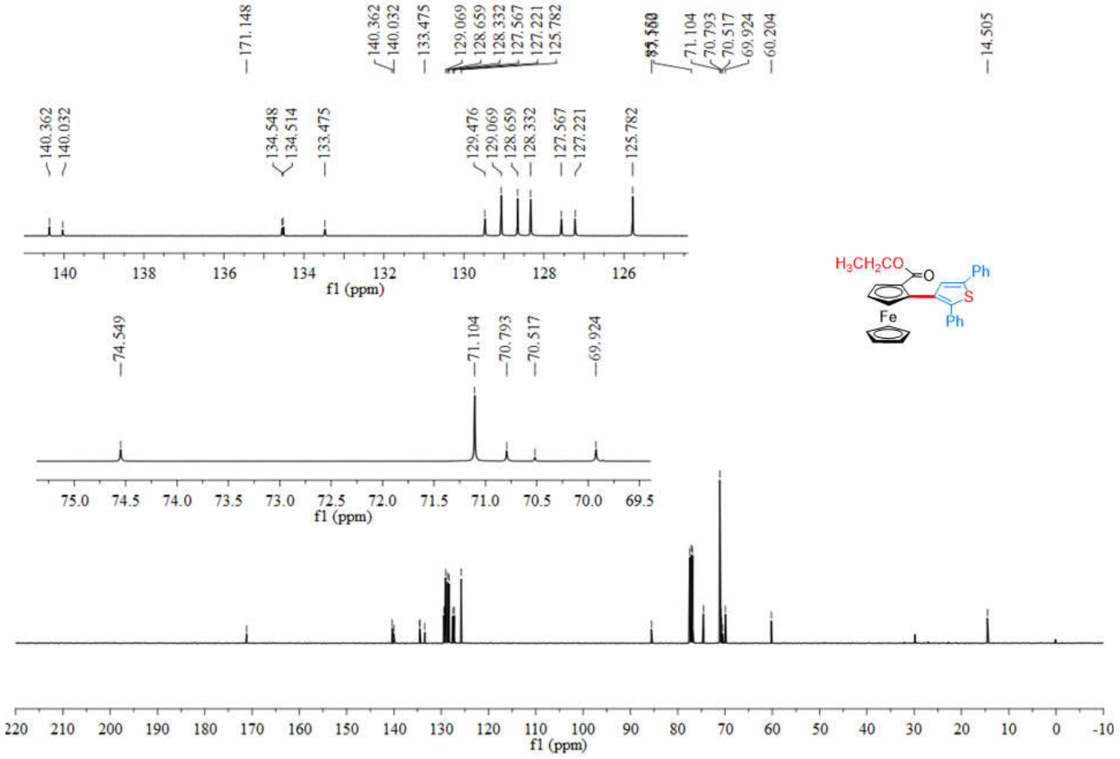

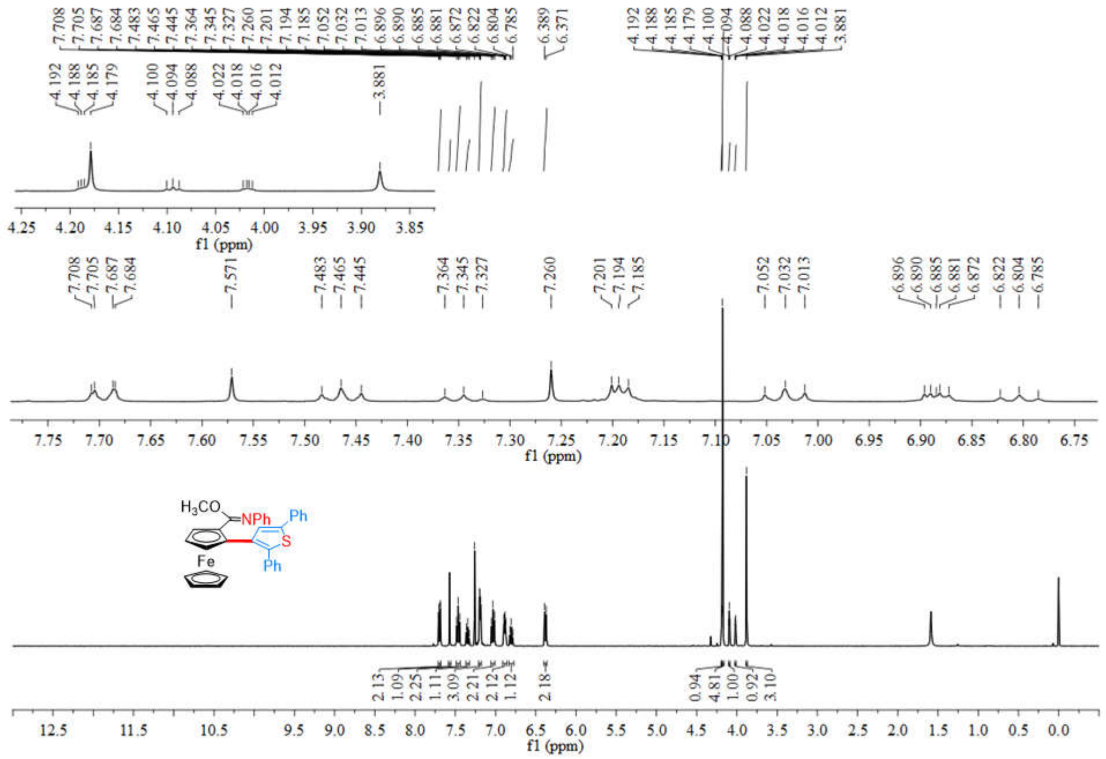
H3CO
NPh
Ph
S
Ph
Fe
f1 (ppm)

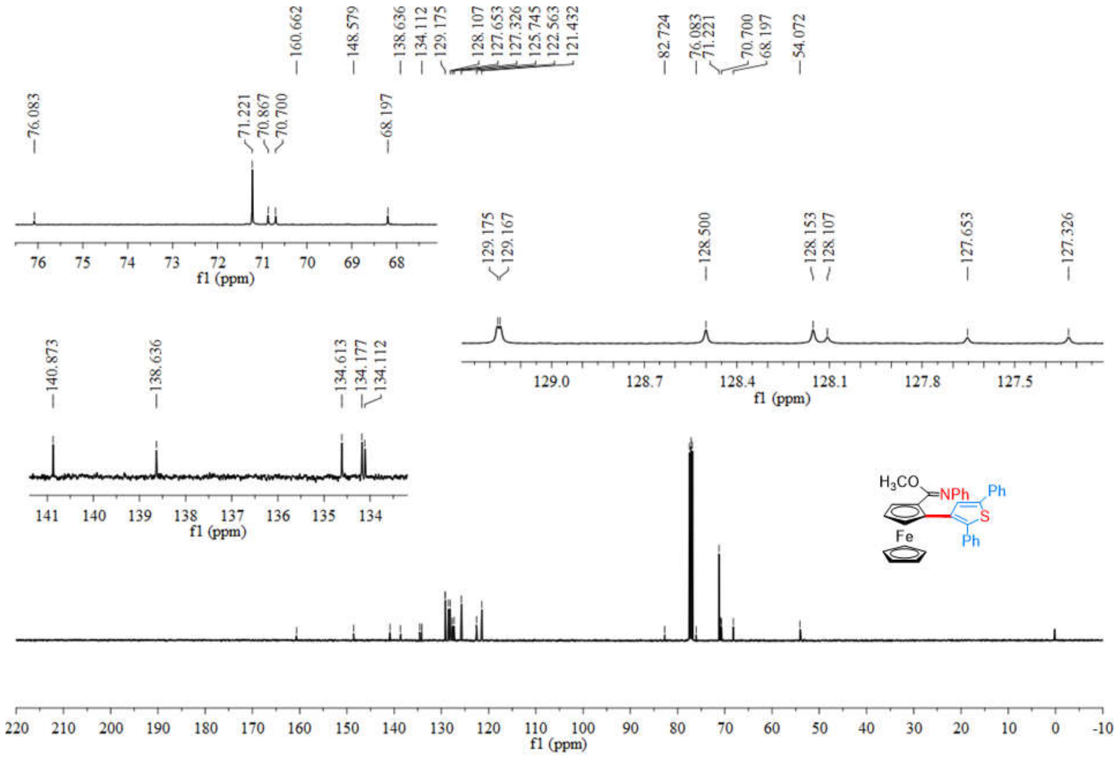
H3CO
NPh
Ph
S
Ph
Fe
f1 (ppm)
